# Interactome Rewiring Following Pharmacological Targeting of BET Bromodomains

**DOI:** 10.1016/j.molcel.2018.11.006

**Published:** 2019-02-07

**Authors:** Jean-Philippe Lambert, Sarah Picaud, Takao Fujisawa, Huayun Hou, Pavel Savitsky, Liis Uusküla-Reimand, Gagan D. Gupta, Hala Abdouni, Zhen-Yuan Lin, Monika Tucholska, James D.R. Knight, Beatriz Gonzalez-Badillo, Nicole St-Denis, Joseph A. Newman, Manuel Stucki, Laurence Pelletier, Nuno Bandeira, Michael D. Wilson, Panagis Filippakopoulos, Anne-Claude Gingras

**Affiliations:** 1 Lunenfeld-Tanenbaum Research Institute at Mount Sinai Hospital, Toronto, ON M5G 1X5, Canada; 2 Structural Genomics Consortium, Nuffield Department of Clinical Medicine, University of Oxford, Oxford OX3 7DQ, UK; 3 Ludwig Institute for Cancer Research, Nuffield Department of Clinical Medicine, University of Oxford, Oxford OX3 7DQ, UK; 4 Department of Molecular Genetics, University of Toronto, Toronto, ON, Canada; 5 Genetics and Genome Biology Program, SickKids Research Institute, Toronto, ON, Canada; 6 Department of Gene Technology, Tallinn University of Technology, Tallinn, Estonia; 7 Department of Gynecology, University of Zurich, Wagistrasse 14, 8952 Schlieren, Switzerland; 8 Center for Computational Mass Spectrometry, University of California, San Diego, La Jolla, CA 92093, USA; 9 Department of Computer Science and Engineering, University of California, San Diego, La Jolla, CA 92093, USA; 10 Skaggs School of Pharmacy and Pharmaceutical Sciences, University of California, San Diego, La Jolla, CA 92093, USA; 11 Heart & Stroke Richard Lewar Centre of Excellence in Cardiovascular Research, Toronto, ON, Canada

**Keywords:** BET, bromodomain, proteomic network, JQ1, rewiring, rRNA, nucleolus, KacY, AP-MS, protein crystallography

## Abstract

Targeting bromodomains (BRDs) of the bromo-and-extra-terminal (BET) family offers opportunities for therapeutic intervention in cancer and other diseases. Here, we profile the interactomes of BRD2, BRD3, BRD4, and BRDT following treatment with the pan-BET BRD inhibitor JQ1, revealing broad rewiring of the interaction landscape, with three distinct classes of behavior for the 603 unique interactors identified. A group of proteins associate in a JQ1-sensitive manner with BET BRDs through canonical and new binding modes, while two classes of extra-terminal (ET)-domain binding motifs mediate acetylation-independent interactions. Last, we identify an unexpected increase in several interactions following JQ1 treatment that define negative functions for BRD3 in the regulation of rRNA synthesis and potentially RNAPII-dependent gene expression that result in decreased cell proliferation. Together, our data highlight the contributions of BET protein modules to their interactomes allowing for a better understanding of pharmacological rewiring in response to JQ1.

## Highlights

• Treatment with JQ1 induces an extensive BET proteins interactome rewiring
• Structural and biophysical studies expand the target space for BET bromodomains
• Two distinct short linear motifs mediate BET ET domain interactions
• BRD3 negatively regulates proliferation through Pol I and II mechanisms

## Graphical Abstract

**Figure:**
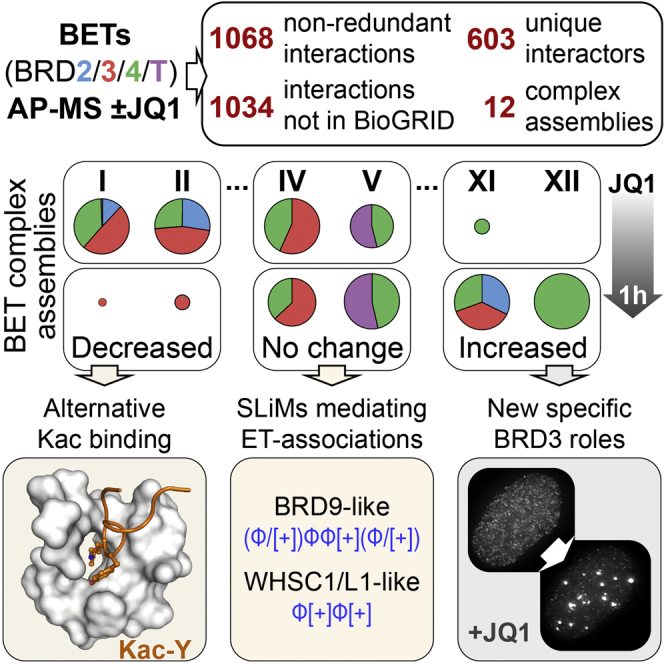

## Teaser

Lambert, Picaud, et al. report that pharmacological bromodomain inhibition rewires the interactome of the Bromo and Extra-Terminal (BET) proteins, resulting in loss (e.g., histones), maintenance, or gain of interactions. They reveal new binding modalities and an unsuspected negative role for BRD3 in proliferation.

## Introduction

Eukaryotic transcription is a tightly controlled process that depends on the formation of protein complexes regulated by post-translational modifications (Bernstein et al., 2007). Bromo and extra-terminal (BET) proteins provide a recruitment platform initiated by the recognition of acetylated lysines (Kac) by their tandem bromodomains (BRD, Dhalluin et al., 1999, Jacobson et al., 2000, Owen et al., 2000). BETs, like all of the 42 human BRD-containing proteins, contain additional interaction domains that recruit other proteins to the acetylated protein target, forming complex assemblies and contributing to processes such as chromatin remodeling and transcription (reviewed in Fujisawa and Filippakopoulos, 2017).

**Figure 1:**
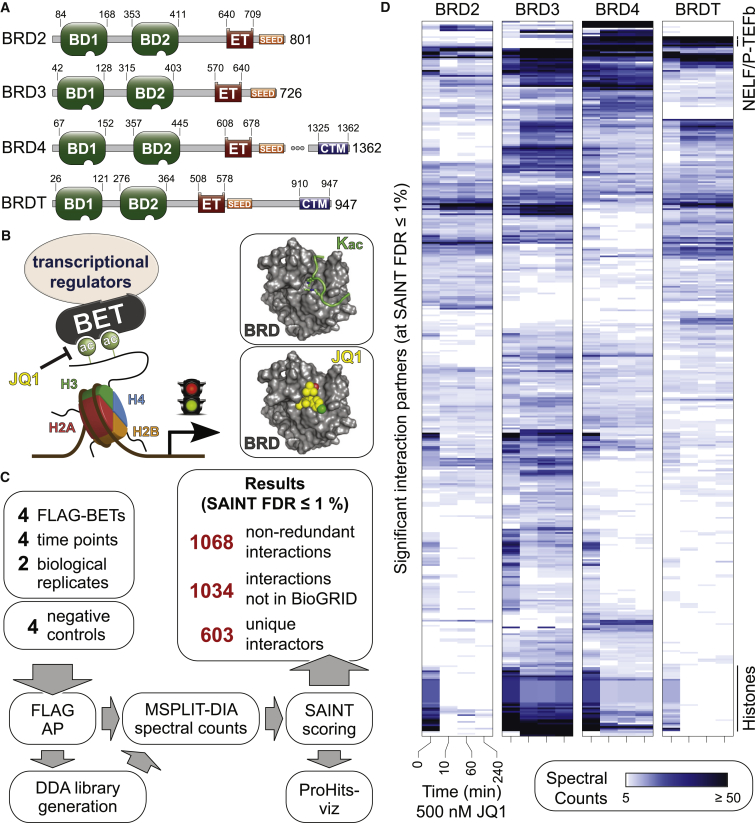
BET Proteins Are Molecular Scaffolds Interacting with Distinct Proteins (A) Modular organization of BET proteins (domain boundaries in amino acids). (B) BETs scaffold transcriptional regulators to acetylated histones. Inset: JQ1 competes with Kac-containing peptides for BRD association. (C) Overview of experimental setup used to quantify the BET interaction network upon JQ1 treatment. (D) Heatmap of BET high-confidence interaction partners identified by AP-MS in the JQ1 time course. See also Figure S1 and Tables S1 and S2.

The BET sub-family comprises four proteins in humans (BRD2, BRD3, BRD4, and the testis-specific BRDT) that harbor at their amino-termini two BRD modules with distinct specificity for Kac on histones and on a growing list of non-histone targets, reviewed in Fujisawa and Filippakopoulos, 2017), followed by an extra-terminal (ET) domain that mediates protein-protein interactions (Rahman et al., 2011; Figure 1A). BRD4 and BRDT also contain a C-terminal motif (CTM) that facilitates the recruitment of transcriptional regulators, including the positive transcription elongation factor b (P-TEFb; Figure 1B). BETs, and in particular BRD4, have been implicated in human disease, especially cancer. Translocations of *BRD4* (and more rarely *BRD3*) to the *NUTM1* (NUT midline carcinoma family member 1) gene cause a rare but aggressive form of squamous cell carcinoma (French et al., 2004). Furthermore, BRD4 levels are upregulated in a variety of tumors, leading to aberrant expression of growth-promoting genes, including the MYC oncogene (Delmore et al., 2011, Mertz et al., 2011, Zuber et al., 2011) and other transcription factors such as ERG, c-Myb, E2F1, and nuclear factor κB (NF-κB) (reviewed in Fujisawa and Filippakopoulos, 2017).

The importance of BET proteins in cancer, together with the recognition that BRD-Kac interactions are druggable, has made them attractive targets for pharmaceutical intervention (Filippakopoulos et al., 2010, Nicodeme et al., 2010). Direct targeting of BET-BRDs by small-molecule inhibitors such as the high-affinity and pan-BET specificity thienodiazepine (+)-JQ1 (hereafter referred to as JQ1) enables their displacement from Kac (Figure 1B). JQ1 displays anticancer activity in cell-culture models, patient-derived xenograft models of NUT midline carcinoma, and in several Myc-driven cancers (reviewed in Bradner et al., 2017). More than 20 clinical trials have been recently initiated to investigate the efficacy of BET-BRD inhibitors in an array of cancers (clinicaltrials.gov), with overall responses being limited and short lived. Yet, preclinical data suggest that, in combination with existing therapies, BET-BRD inhibitors can potentiate the effects of cell cycle, immune checkpoint, and DNA damage repair inhibitors (Doroshow et al., 2017). An improved understanding of BET protein biochemistry is essential to facilitate the successful progression of BET-BRD inhibitors into the clinic.

Here, we establish the interactome of each BET protein, revealing a rich network of interactions that are modulated following treatment with JQ1. By analyzing the quantitative behavior of 603 interactors, we define three classes of proteins: those for which interaction decreases following JQ1 treatment, those whose association remains relatively unchanged, and those that are unexpectedly increased following BRD inhibition. Multiple decreased interactors harbor sequences that can directly associate with BET-BRDs in canonical or new BRD-mediated structural binding modes, and we propose that the tandem BRDs present in each BET protein may be capable of simultaneously recruiting both a histone and a second interactor. Consistent with previous reports, we define two distinct sequence motifs that bind to the BET ET domain in a Kac-independent manner. Last, by examining gained interactors, we identify an unsuspected function for BRD3 in ribosome biogenesis, and a negative role in cell proliferation that is supported by mining genome-wide CRISPR-Cas9 datasets. Our findings suggest that pan-BET inhibitors may have the unintended consequence of inhibiting the growth repressive functions of BRD3, in parallel to inhibiting the desired BRD4 positive functions. Taken together, our systematic proteomics, biophysical, structural, and cell biological studies provide a framework to better understand BET biochemistry and promote the rational development of new inhibitors.

## Results

### Interactome Profiling Reveals Shared and Distinct BET Protein Interaction Partners

To establish an interaction network for the BETs, we performed affinity purification coupled with mass spectrometry (AP-MS) on 3×FLAG-tagged BET proteins using optimized protocols enabling recovery of interactors for both DNA-bound and unbound proteins (Lambert et al., 2014). Samples analyzed by data-independent acquisition MS across two biological replicates were scored against negative controls with Significance Analysis of INTeractome (SAINT, Teo et al., 2014). We identified 650 high-confidence BET interactions (FDR ≤1% and R^2^ of 0.93; Table S2A) involving 357 unique preys, 329 (∼92%) of which have not been previously reported in the BioGRID repository (Figure S1A), though 106 of the interactors were detected with at least one BET bait in a previous study (Dawson et al., 2011; Table S2B). The 357 interactors were highly enriched for expected Gene Ontology terms “nuclear lumen” (Cellular Component), “nucleic acid binding” (Molecular Function), and for the “gene expression” REACTOME pathway (Table S2C).

Our proteomic screens revealed multiple interactions for each BET protein (Figure S1A), with 26 interactors shared across all BETs and 177 detected with a single BET bait (Figure S1B). Several interactors shared across all BETs (e.g., CHD8, PWWP2B, and WHSC1L1) were previously identified only as BRD4 interactors (Rahman et al., 2011, Shen et al., 2015), highlighting the importance of performing a systematic family-wide assessment. BET proteins formed an interconnected interaction network, notably through shared interactions with histones that included association with other BRD-containing proteins, suggesting an intricate physical interplay between Kac readers on chromatin, and reinforcing the scaffolding roles of BET proteins in regulating transcriptional programs (Table S2D).

Consistent with the existence of a CTM (Figure 1A), BRDT and BRD4 associated with P-TEFb, as previously shown (Gaucher et al., 2012, Jang et al., 2005), and with the negative elongation factor (NELF) complex, as was suggested for BRD4 (Patel et al., 2013). Components of negative transcriptional regulators such as the nucleosome remodeling and deacetylase (NuRD) complex preferentially associated with BRD3 and BRD4 while the RNAPII subunits POLR2A and POLR2L, as well as most Mediator subunits, were only identified as high-confidence partners for BRD4 (Table S2D). Our survey therefore revealed rich interactomes for each BET protein linking them to both activating and repressive functions.

### JQ1 Rewires the BET Protein Interaction Landscape

To evaluate the impact of BRD inhibition on the BET interaction landscape, we performed AP-MS on each BET after treating cells with 500 nM JQ1 for 10, 60, or 240 min (untreated samples, t = 0, are described above). We identified 2,278 protein-protein interactions (1,068 of which are non-redundant, including 1,034 not previously reported in BioGRID) involving 603 unique significant interaction partners (FDR ≤1%) across all conditions tested. Treatment with JQ1 elicited rapid (i.e., within 10 min) changes of the interactions established by each BET, which were sustained, and in some cases enhanced, at 60 and 240 min (Figures 1C and 1D).

**Figure 2:**
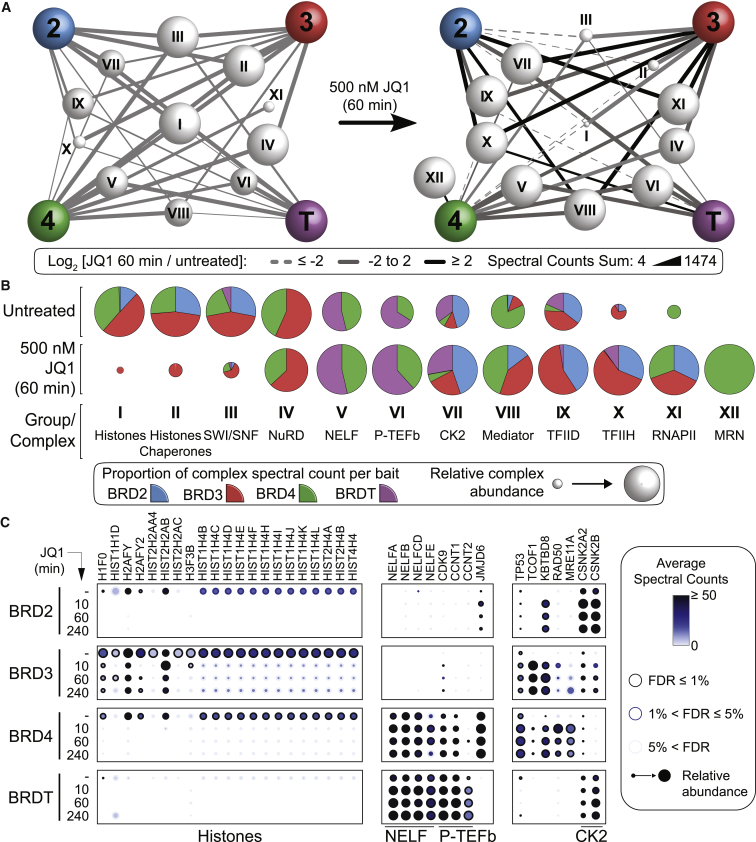
Pharmacological BRD Inhibition Modulates the BET Interactome (A) Network view of selected protein complexes or families (groups I–XII; names in B, details in Table S2D) associated with BETs. Node size displays the relative abundance in cells untreated or treated with JQ1 for 1 hr. (B) Relative spectral count contributions of individual BET proteins to selected groups or complexes. (C) Dot plots of selected interaction partners associated with individual BETs after JQ1 treatment. See also Figure S1 and Tables S1 and S2.

Collapsing the transcription-related BET interactors to 12 functional groups or protein complexes (encompassing 149 unique interactors; Tables S2E and S2F) enabled us to capture a network view of the transcriptional interactome changes caused by 1 hr of JQ1 treatment (Figure 2A). While association with histones (group I) was pronounced in untreated cells, JQ1 treatment resulted in an expected and dramatic decrease in recovery, validating our experimental system (Figures 2A–2C and S1C; data in Table S2A). JQ1 treatment also led to a marked reduction in the recovery of histone chaperones (group II), including the FACT and CAF1 complexes, as well as DAXX and DEK. Recovery of the switch/sucrose non-fermentable (SWI/SNF) nucleosome remodeling complex components (group III) with all BETs was also strongly reduced by JQ1 treatment (Figures 2A, 2B, S1C, and S1D). Altogether, JQ1 treatment for 1 hr resulted in a global decrease (log_2_ fold change [LFC] ≤−2) of 367 interactions (Figures 1D and 2A; Table S2E). Some functional groups displayed sustained interactions (LFC within ±2; 262 interactions) with BETs following JQ1 treatment (Figures 2A and 2B), including CHD4 and JMJD6 (Figures 2C, S1C, and S1D), suggesting that they are recruited in a Kac-independent manner. Intriguingly, JQ1 treatment also enhanced (LFC ≥2) 248 interactions, including the association between BRD4 and the MRN complex (group XII in Figures 2A–2C and S1E), and that between BRD4 and TP53 (Figures 2C and S1E). New prominent interactions between BRD2/3/4 and KBTBD8, and between BRD3 and TCOF1 were also observed (Figure 2C).

To further validate these interactions and quantitative behaviors following JQ1 treatment, we performed immunoprecipitation followed by MS using antibodies to endogenous BRD2, BRD3, and BRD4 (using immunoglobulin G [IgG] as a negative control) in the presence or absence of JQ1, in both HEK293 cells, and in the chronic myeloid leukemia K562 cells, where we previously examined the transcriptional outcome of BET targeting with JQ1 (Picaud et al., 2016). Despite issues of cross-reactivity and binding site masking (see STAR Methods), we were able to validate 239 of the 425 interactors of BRD2/3/4 detected across the 0 and 1-hr time points (56.2% validation; Tables S2G and S2H). 106 of the 319 interactors detected in the non-treated condition (31%) were also previously identified in the AML HL-60 cells (Dawson et al., 2011). Collectively, the endogenous datasets provided validation to 67% of the FLAG interactions detected in the absence of JQ1, confirming the validity of our dataset. Importantly, in HEK293s—and to a lesser extent in K652 cells—the quantitative trends observed following JQ1 treatment were also recapitulated (Figure S1C; Table S2I). In particular, components of complexes such as Mediator, TFIID, TFIIH, and RNAPII increased in abundance following JQ1 treatment, while histones and histone chaperones decreased.

Taken together, our data highlight the complex role of JQ1 treatment in the remodeling of the BET interactome, as recently suggested (Bhagwat et al., 2016), with both losses and gains in associations, as well as multiple interactions that are Kac independent. To gain additional insight, we further explored these three different classes of binding behavior.

### Di-Kac Motifs on Histones and Non-histone Proteins Are Recognized by BET BRDs

The large number of BET interactions lost following JQ treatment in our proteomic screen prompted us to re-examine Kac-dependent contributions to BET interactions with both histone and non-histone proteins. Following up on initial findings for BRDT (Morinière et al., 2009), we previously found preferential association of histone H4 di-Kac motifs with an optimal linker of two amino acids (Kac-XX-Kac; preference for glycine, G, at X1) and identified a common structural template whereby both Kac insert within the BET BRD cavity (Filippakopoulos et al., 2012). Here, we applied a peptide SPOT binding approach to all histones, confirming their preferred association to Kac-XX-Kac sites where XX are GG, GS, DG, AA, AP, AV, AQ, AR, SA, VL, LN, TA, and TP, though we also detected several instances of binding via longer linkers (e.g., Kac-X_3-4_-Kac; Tables S3A and S3B).

**Figure 3:**
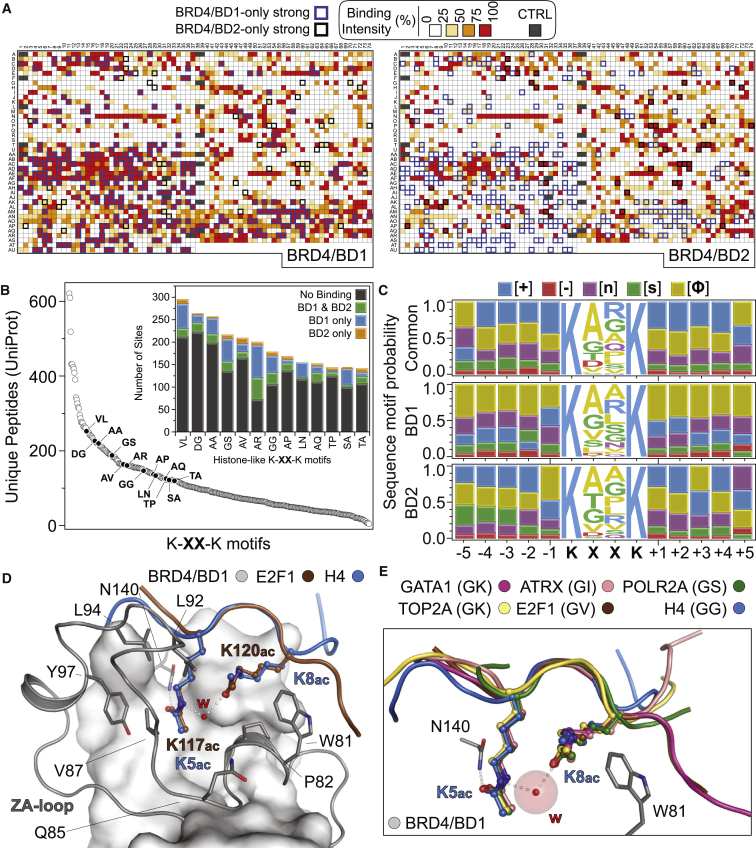
BET BRDs Initiate Interactions with Non-histone Kac-XX-Kac Peptides (A) Peptide SPOT validation of histone-like peptides containing a Kac-XX-Kac motif. The heatmap shows binding intensities against the first (BD1) and second (BD2) BRDs of BRD4. Peptides exhibiting strong (≥75% of maximum) intensity toward one domain, with a ≥2-fold lower intensity toward the other domain are highlighted. (B) Unique peptides containing K-XX-K motifs found in the human proteome. The inset highlights the binding results from (A) toward BRD4 BRDs. (C) Peptide LOGOs derived from very strong (≥85% of maximum intensity) binding in the SPOT arrays shown in (A). (D) Crystal structure of BRD4/BD1 bound to an E2F1 di-Kac peptide (K117ac-XX-K120ac motif) or the previously published histone H4 K5ac/K8ac peptide (PDB: 3UVW). (E) Structural overlay of BRD4/BD1 complexes with Kac-GX-Kac-bearing peptides shown in cartoon, highlighting the topology of the BRD cavity with respect to the conserved asparagine (N140) and the bulky tryptophan of the WPF shelf (W81). See also Figure S2 and Tables S1, S3, S4, and S5.

We previously proposed (but did not test; Filippakopoulos et al., 2012) that the electrostatic potential of residues surrounding the BRD Kac-binding cavity contributes to binding specificity by selecting for favorable sequences outside the Kac-XX-Kac motif. The human proteome contains over 43,000 unique K-XX-K motifs, ∼2,100 (∼4.8%) harboring “histone-like” XX sequences. To determine whether flanking sequences influence binding to BET BRDs, we analyzed di-Kac histone-like peptides by SPOT arrays with the two isolated BRDs of BRD4 (BD1 and BD2, Tables S3C and S3D). Strikingly, we observed at least moderate (≥50%) binding intensity (compared to maximum arising from multiple hexa-His-controls) toward 41.7% of all peptides tested (928 and 549 peptides for BD1 and BD2 respectively), with 28.9% of all peptides binding strongly (≥75% of maximum) to either BD1 or BD2, and binding to BD1 systematically more prominent (9.6% bound to both domains; 15.5% only to BD1 and 3.8% only to BD2; Figures 3A and 3B). Diverse flanking sequences drove binding to either domain, with basic sequences recognized by both, while leucine-rich sequences were enriched primarily by BD1 (Figure 3C), consistent with the charge dispersion on the surface surrounding the binding cavities and the more hydrophobic character of BD1 (Figure S2A).

Based on our earlier work defining the flexible G at the X1 position as a binding preference for histone H4 (Filippakopoulos et al., 2012), we fixed this residue to screen a subset (91) of all 2521 unique K-GX-K motifs found in the human proteome, focusing on nuclear proteins with Kac sites annotated in the PhosphoSitePlus database. Again, both domains exhibited some degree of binding, with BD1 systematically exhibiting stronger binding (Figure S2B; Tables S3C and S3D). Isothermal titration calorimetry (ITC) confirmed interactions of selected peptides with both domains, albeit with different affinities (Table S4A; Figures S2C and S2D). To better understand the binding behavior of BET BRDs toward these non-histone peptides, we structurally characterized interactions with a di-Kac peptide found in E2F1 (K117ac/K120ac; Figures 3D and S2E). Both lysines inserted within the Kac-binding cavity of BRD4/BD1, with K117ac directly engaging the conserved asparagine and K120ac initiating a water-mediated interaction with K117ac, similar to histone H4 peptides (Filippakopoulos et al., 2012). Additional peptides carrying a Kac-GX-Kac motif, including those from GATA1, ATRX, POLR2A, and TOP2A, adopted the same histone H4-like structural association with BRD4/BD1 (Figures 3E and S2F–S2I). Our data therefore suggest that Kac-XX-Kac motifs beyond those found in histones can be recognized by BET BRDs.

### Di-Kac Motifs Separated by Long (>2) Linkers Are Recognized by BET BRDs

**Figure 4:**
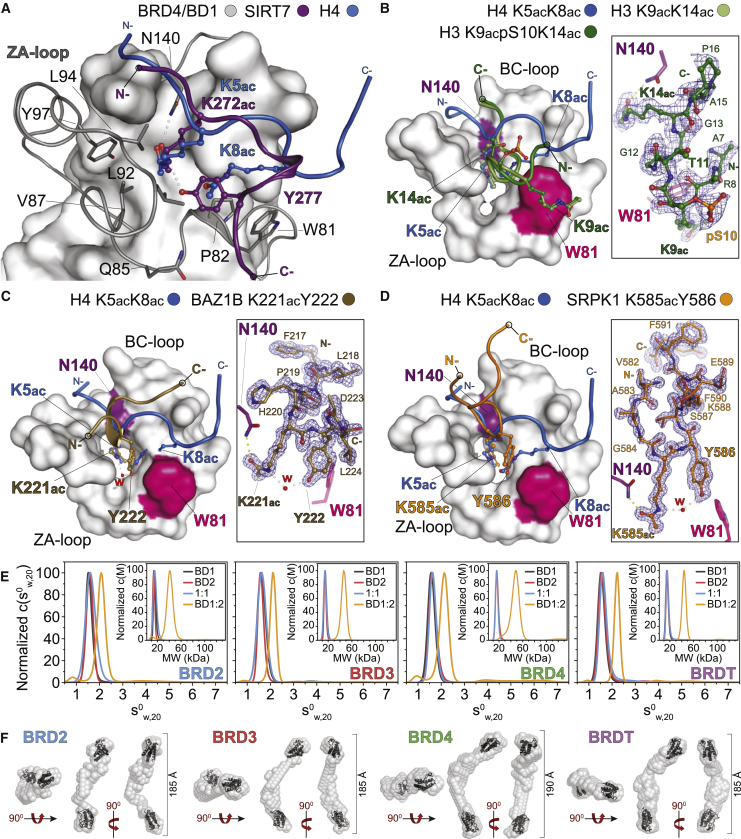
Different Modes of BET BRD Recognition of Kac (A–D) Crystal structures of BRD4/BD1 bound to histone H4 (PDB: 3UVW) and indicated peptides derived from SIRT7 (A), di-Kac H3 (B), BAZ1B (C), and SRPK1 (D). The peptide 2Fc-Fo maps contoured at 2σ are shown in the insets. (E) Sedimentation velocity experiments of BET BRDs individually (BD1, BD2), in equimolar mixtures (1:1) or tandem constructs (BD1:2) demonstrating lack of self- or hetero-association. (F) *Ab initio* shapes of BET tandem BRD constructs restored from SAXS data; mean distances (n = 100) are shown next to the models, in agreement with the hydrodynamic shape calculated using prolate ellipsoid models in (E). See also Figure S3 and Tables S1, S3, S4, and S5.

Our structural analysis identified multiple cases of non-histone proteins binding to BRD4 BRDs through histone-like motifs in the same structural template we initially described for H4. However, when crystallizing a Kac-XX-Kac SIRT7 peptide (K272ac/K275ac) with BRD4/BD1, we surprisingly found only the first Kac within the binding cavity. Strikingly, a bulky tyrosine residue located 5 residues downstream of the first lysine (Y277) instead inserted into the BRD cavity, stabilizing K272ac via a hydrogen bond (Figures 4A and S3A). This, together with our finding that several histone peptides with longer linkers readily bind BRD4 BRDs (Kac-X_3–4_-Kac; Tables S3A and S3B), suggested that associations could be mediated via more diverse protein sequences and structural templates than initially suspected. To understand the mode of engagement of a single BET BRD toward adjacent Kac marks separated by longer linkers, we crystallized BRD4/BD1 with a histone H3 peptide carrying K9ac and K14ac (Kac-X_4_-Kac motif) identified in our SPOT data (Tables S3A and S3B). Interestingly, only K14ac bound within the BRD cavity, while K9ac packed outside of the cavity, next to W81 (Figure 4B). This resulted in an inverted peptide orientation compared to Kac-XX-Kac peptides. While K14ac superimposed well with K5ac from a H4 K5ac/K8ac peptide, the longer backbone linking it to K9ac superimposed with K8ac, sterically filling the remaining volume of the BRD cavity, and resulting in a small helical turn (Figure S3B). Importantly, the conformations of S10 and T11, and their contributions to the stabilization of this helical topology, also suggested a functional role for their post-translational modification during binding. Indeed, while both BRD4 BRDs bind in solution to H3 K9ac/K14ac (Figure S3C; Table S4A), phosphorylation of S10 had no effect on BD1 binding but abolished binding to BD2, and phosphorylation of T11 abolished binding to both BRD4 BRDs (Table S4A). These observations suggest that adjacent sites separated by longer linkers can be recognized by single BET BRDs (though with weaker affinities than XX linkers), with linker sequences offering the potential for regulating interactions.

### Tyrosine at +1 Can Substitute for a Second Kac in BET BRD Binding

Intrigued by these unprecedented modes of binding, we next analyzed the 181 unique proteins that decreased below our limit of detection after 1-hr JQ1 treatment in the AP-MS dataset: these contained 903 unique annotated Kac sites in PhosphoSitePlus (Tables S3F–S3I). Focusing on BRD4, which associated with the largest number of annotated sites (456), we examined the relative enrichment of amino acids with respect to each central Kac (Figure S3D). Weak enrichment of lysine at +3 suggested that it is unlikely that Kac-XX-Kac sequences drive these interactions. By contrast, tyrosine was clearly enriched at the +1 position for several regulated preys, including histone H1 and BAZ1B, and peptide SPOT arrays confirmed binding of BRD4 to these sites (Table S3J). We crystallized a K221ac BAZ1B peptide with BRD4/BD1 and observed typical Kac-engagement by N140 in the high-resolution structure, while the adjacent tyrosine (BAZ1B Y222) also inserted into the binding cavity, linking to K221ac via a water-mediated bridge (Figure 4C). Although K221ac superimposed well with K5ac in the H4 K5ac/K8ac complex with BRD4/BD1, the peptide backbone followed a different path, which induced structural changes on the surface of the BRD4/BD1 module via side-chain re-alignment of D144, I146, and L148, while inserting BAZ1B Y222 in the same space as H4 K8ac (Figure S3E).

We asked therefore whether this mode of binding would be conserved in the presence of a second Kac within a Kac-YX-Kac motif; of the 1,091 K-YX-K sequences in the human proteome, 84 have been found acetylated (PhosphoSitePlus) on the first lysine (Kac-YX-K), and 39 are di-acetylated (Kac-YX-Kac). SRPK1 contains a Kac-YS-K motif, and its paralog, SRPK2, has been reported as a BRD3 interactor in the BioGRID database. We crystallized an SRPK1 K585ac/K588ac peptide with BRD4/BD1 and found K585ac inserted within the cavity together with Y586, while the K588ac remained outside the cavity (Figure 4D). Importantly, validation of binding by ITC revealed similar affinities for Kac-Y epitopes compared to other Kac-XX-Kac motifs (BRD4/BD1 versus SRPK1 or H4, K_D_ = ∼9.9 μM; Table S4A; Figure S3F). In addition, both H4 and SRPK1 peptides were competitively displaced by JQ1 in ALPHAScreen assays, supporting that binding occurs within the BRD Kac cavity in solution (Figure S3G). Further evaluation of binding by ITC suggested, however, that these interactions with BRD4 have different thermodynamic properties, with H4 association driven by enthalpic contributions (consistent with multiple electrostatic interactions present in the structural model, a larger surface presented to the protein and a negative change in heat capacity, ΔC_p_), while SRPK1 association was also driven by hydrophobic and entropic contributions (consistent with a positive change in ΔC_p_; Figure S3H; Table S4B). Taken together, our data suggest that Kac-Y motifs can compensate for the absence of a second Kac, resulting in a similar structural template that fits within the volume of BET BRD sites.

### N-Terminal Tandem BET BRDs Adopt Extended Conformations in Solution

Given this considerable expansion in the possible BET BRDs sites, we next examined whether it would be possible for BET proteins to engage independent target sites by employing both N-terminal BRDs, which are linked by long (155–205 aa) flexible regions. Analytical ultracentrifugation revealed that the individual domains do not self- or hetero-associate, while constructs containing both domains adopt more extended linear conformations (Figure 4E; Table S4C). These observations were further supported by in-solution small-angle X-ray scattering measurements of tandem BET BRD constructs, which were monomeric and flexible, while *ab initio* and ensemble optimizations supported extended conformations (Figures 4F and S3I). Taken together, our data suggest that tandem BET BRDs adopt extended conformations in solution, possibly allowing for the targeting of distinct sites by recognizing different Kac epitopes *in trans*, and thus contributing to the assembly of large BET complexes on chromatin.

### The BET ET Domain Provides a Protein Recruitment Platform

Despite the large number of Kac-dependent interactions identified in our proteomic screen, several of the interactors were relatively insensitive to treatment with JQ1. BET family proteins contain an ET domain consisting of three helices and an acidic surface shaped in a continuous ridge (Lin et al., 2008; Figure S4A) previously reported to mediate diverse protein interactions (Konuma et al., 2017, Rahman et al., 2011, Sansam et al., 2018, Zhang et al., 2016) and to associate with viral peptides (Crowe et al., 2016). We hypothesized that the conserved ET domain may recruit a fraction of the identified Kac-independent interactions.

**Figure 5:**
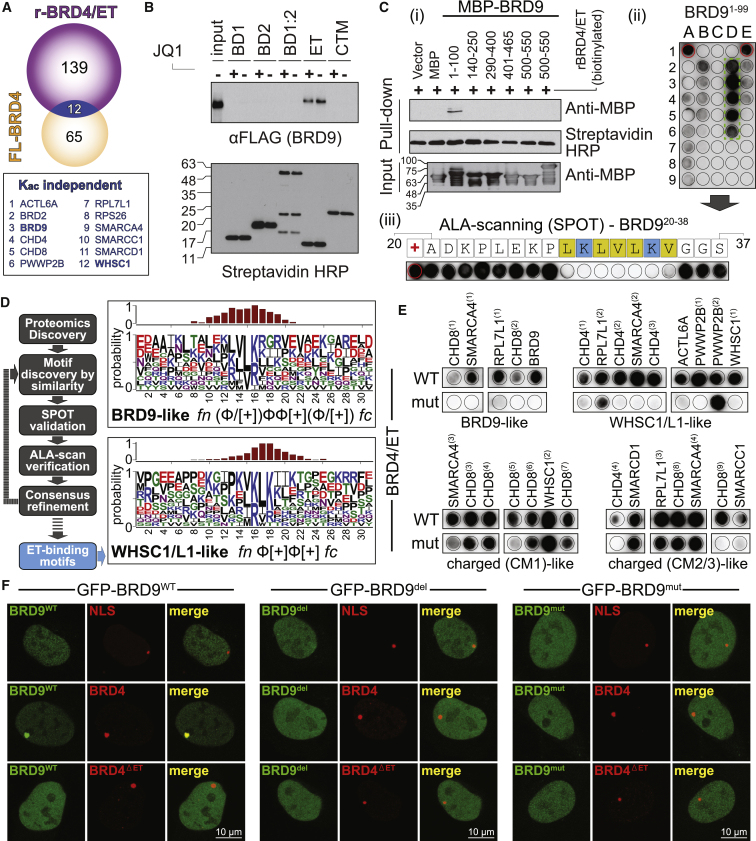
Contributions of the ET Domain to the BET Interactome (A) Overlap of full-length (FL)-BRD4 interactors (within ∼ ± 2 LFC in spectral count ratio following JQ1 treatment) and BRD4/ET domain highlighting 12 common proteins. (B) Recovery of FLAG-tagged BRD9 from pull-downs with indicated recombinant BRD4 domains. (C) Identification of the BRD9 binding site mediating interactions with BRD4/ET: (i) recovery of MBP-tagged BRD9 fragments with recombinant BRD4/ET domain; (ii) peptide SPOT array of BRD^91–99^ blotted against the BRD4/ET domain; (iii) SPOT alanine-scanning of BRD9^20–38^ against BRD4/ET. (D) Schematic of BET ET-motif discovery employing AP-MS, SPOT arrays, and alanine scanning. Refined LOGO motifs are shown on the right. The bar charts on the top of each LOGO represent the relative residue contribution to the overall peptide binding following SPOT-ALA scanned array quantifications. (E) Assessment of the behavior in SPOT assays of the indicated motif classes upon polarity reversal of the ET surface (BRD4/ET wt vs mut). (F) Cellular validation of ET-specific interactions using LacO/LacR chromatin immobilization. U-2 OS cells with a stably integrated LacO array were transfected with FL-mCherry-BRD4 (WT or ΔET) and FL-GFP-BRD9 (WT or mutant). See also Figures S4 and S5 and Tables S1, S2, and S3.

We tested this hypothesis for BRD4, which associated with 67 proteins whose abundance was relatively unaffected by JQ1 (i.e., within ± 2 LFC). AP-MS with a recombinant BRD4-ET domain identified 151 high-confidence interactors (Tables S2E and S2J), 12 of which overlapped with proteins insensitive to JQ1 in AP-MS (Figure 5A). We further explored the interaction between BRD4 and the poorly characterized BRD-containing protein BRD9 by performing pull-downs with biotinylated recombinant BRD4 domains against 3×FLAG-BRD9 expressed in HEK293 cells. Only the ET domain was able to pull down BRD9 and, as expected, this interaction was not affected by JQ1 treatment (Figure 5B). Reciprocal AP-MS with 3×FLAG-BRD9 identified endogenous BET proteins as interactors for both the full-length protein and a BRD9 1–100 construct (Figure S4B; Table S2K). Streptavidin pull-down of recombinant BRD9 proteins with biotinylated recombinant BRD4/ET (Figure 5C/i) confirmed a direct interaction with the N terminus of BRD9, which was further validated by analytical ultracentrifugation (Figure S4C). Peptides spanning BRD9 1–100 were synthesized on cellulose SPOT arrays and probed with the recombinant BRD4/ET domain, further narrowing down the interaction interface to amino acids 20–38 of BRD9 (Figure 5C/ii; Table S3K). Alanine scanning of this region revealed a short linear motif (SLiM) combining basic and hydrophobic residues (LKLVLKV) essential in initiating the interaction with BRD4/ET (Figure 5C/iii; Table S3L), in a region of the protein predicted to be disordered (Figure S4D).

To test whether similar SLiMs are present in the other proteins in Figure 5A, we next examined the interaction with the methyltransferases WHSC1 and WHSC1L1 (also known as NSD2 and NSD3). WHSC1L1 was previously found to interact with the ET domain of BRD4 (Rahman et al., 2011), and more recently the interaction interface was mapped to a region between amino acids 98–265 (Shen et al., 2015). Peptide SPOT arrays within this region identified a peptide (amino acids 147–162; Figure S4E; Table S3N) that was further profiled by alanine scanning to reveal a short basic and hydrophobic SLiM distinct from that in BRD9 that was responsible for interaction with BRD4/ET (IKLKI; Figure S4E; Table S3O).

Based on the BRD9 and WHSC1/L1 SLiMs defined above, we identified 135 similar peptide regions in our 12 candidate ET-interacting proteins. Of those, 29 motifs in 11 independent proteins bound in SPOT assays (Figure S4F; Tables S3P–S3R). We iteratively expanded our analysis to the remaining 55 proteins relatively unaffected by JQ1 treatment (Tables S3S–S3Z and S2E) and defined BRD9-like (*fn*-(Φ/[+])ΦΦ[+](Φ/[+])-*fc*) and WHSC1/L1-like (*fn*-Φ[+]Φ[+]-*fc*) ET-binding motifs (Figure 5D; Tables S3AA and S3AB, where Φ is one of M, L, V, I, F and [+] is K or R), as well as several positively charged motifs (Figure S5A). To rule out non-specific interactions, we reversed the polarity of the acidic interface of the BRD4/ET domain and evaluated binding to the different SLiMs. While BRD4/ET^WT^ produced strong SPOTs in peptide arrays for all identified SLiMs, alanine scanning against wild-type (WT) or mutant ET domains showed similar patterns for charged peptide SLiMs (Figure S5B), suggesting that these are non-specific. By contrast, binding to BRD9-like and WHSC1/L1-like SLiMs was lost when tested against the BRD4/ET^mut^, indicating that these SLiMs initiate BRD4-specific interactions (Figure 5E).

To assess whether the identified SLiMs are responsible for association in cells, we immobilized mCherry-BRD4-LacR onto chromatin through a LacO array in U-2 OS cells. While GFP-BRD9^WT^ co-localized with mCherry-BRD4-LacR, deletion of the ET domain as well as deletion or mutation of the BRD9 SLiM resulted in loss of co-localization (Figure 5F). We observed the same behavior with other BRD4 interacting partners, including WHSC1L1 (Figure S5C) and proteins whose recovery was not affected by JQ1 in AP-MS with full-length BRD4 and contained BRD9- or WHSC1L1-like ET interaction motifs, such as ZNF592 (Figure S5D). Taken together, our data demonstrate that the BRD4 ET domain acts as a recruitment platform recognizing distinct SLiMs on target proteins (Figure S5E). While this interaction surface is mutated in cancer (Figure S5F), its precise role in cell proliferation and survival remains to be established.

### BRD3 Localizes to Ribosomal DNA

In the previous sections, we characterized interactions that were either reduced following JQ1 treatment or relatively unaffected and mediated by the ET domain. However, we were puzzled by interactions that were enhanced following targeting of Kac-dependent functions with JQ1. One of the most striking of those was the recovery of TCOF1 with BRD3 (Figure 2C; 0 to 436 spectral count sum). TCOF1 is a critical regulator of ribosome biogenesis localized to the nucleolus (Valdez et al., 2004), a cell compartment not reported to contain BET proteins. Consistent with a possible re-localization to this compartment, several nucleolar proteins also increased in abundance in 3×FLAG-BRD3 immunoprecipitates following JQ1 treatment (Figures 2B and 2C; Table S2A). Altogether, the BRD3 interactors that were increased >2 LFC (102 proteins) were enriched for the GO biological process “ribosome biogenesis” (Table S2L), prompting us to further investigate this observation.

**Figure 6:**
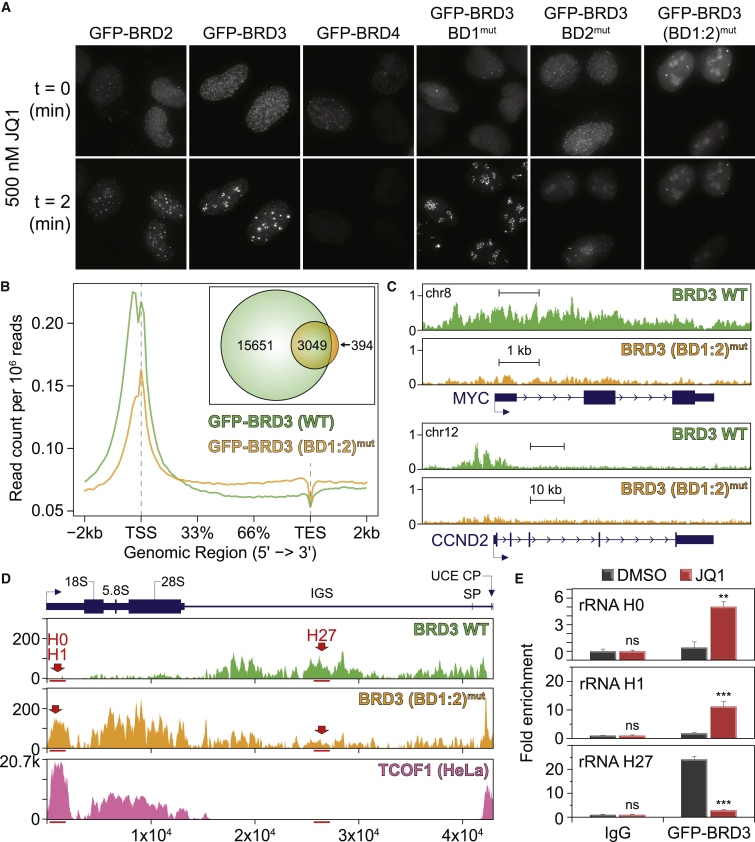
BRD Inhibition Modulates BRD3 Localization (A) Still images of indicated GFP-tagged BET constructs in live U-2 OS cells. (B) Average BRD3 WT or (BD1:2)^mut^ ChIP-seq read counts plotted over genes. TSS, transcription start site; TES, transcription end site. Inset: binding sites detected with each construct. (C) Genome browser tracks showing BRD3 occupancy across the *MYC* and *CCND2* gene loci. y axis: normalized read counts in reads per million per basepair. (D) Schematic representation of a single human rDNA repeat relative to the transcription start site (TSS) of the rDNA repeat (x axis; based on GenBank U13369; SP: spacer promoter; UCE, upstream control element; IGS, intergenic spacer; CP, core promoter). y axis: normalized read counts of BRD3 WT, (BD1:2)^mut^, and TCOF1 (from HeLa cells; Calo et al., 2018). (E) GFP-BRD3 ChIP-qPCR to rDNA H0, H1, and H27 (see D) with and without JQ1 for 1 hr. x axis: signal fold enrichment against rabbit IgG isotype control purifications. Data represent the mean ± SEM (n = 3) of two biological replicates. p values were calculated using Student’s t test and are represented by ^∗∗∗^p < 0.001; ^∗∗^p < 0.01; ns, not significant. See also Figure S6 and Tables S1 and S2.

We first performed live-cell imaging to determine whether BRD inhibition resulted in localization of BET proteins to the nucleolus. Upon JQ1 treatment, BRD3, and to a lesser extent BRD2, rapidly (within ∼20 s) assembled into dense foci (Figure 6A) that persisted for more than 12 hr after inhibitor washout (note that the apparent loss of signal for BRD4 is likely due to its displacement from chromatin, as the protein levels were not markedly found affected by immunoblot). These BRD3 foci co-localized with TCOF1 in the nucleolus, and their formation was prevented by small interfering RNA (siRNA) depletion of TCOF1 (Figure S6A). No noticeable change in the localization of the nucleolar proteins PARP1, POLR1E, and TCOF1 was observed following JQ1 treatment, suggesting that the overall organization of the nucleolus is not altered by JQ1 (data not shown). To better understand the response of BRD3 to JQ1, we employed constructs with point mutations ablating Kac binding in the first (BD1^mut^), second (BD2^mut^), or both ((BD1:2)^mut^) BRDs. BD1^mut^ resulted in a diffuse nuclear localization in asynchronously growing cells, though the mutant BRD3 still accumulated into foci upon JQ1 treatment (Figure 6A). BD2^mut^ prevented dense foci formation upon JQ1 treatment, yet the protein still accumulated in the nucleolus upon JQ1 addition. (BD1:2)^mut^ abolished the response to JQ1 treatment, and generated a nucleolar-enriched signal in both treated and untreated cells. Deletions of regions surrounding BRD3 BRDs (1–42; 128–315 or 403–570) did not impact BRD3 capacity to form dense nucleoli foci (data not shown), further supporting involvement of BRD3 BRDs in this response.

We recently demonstrated that the proximity-dependent biotinylation approach BioID is ideally suited to define the organization of membraneless organelles (similar to the nucleolus, e.g., Youn et al., 2018). BioID permits the identification of proximity partners in the context of a living cell and negates the maintenance of interactions during lysis and purification steps required for AP-MS. Here, we performed BioID experiments in the presence or absence of 500 nM JQ1 for 24 hr (Figure S6B; Table S2M). As expected, prolonged JQ1 treatment resulted in the reduced association of BRD3 with >50 of its AP-MS-identified partners (Table S2A) with a concurrent increase in nucleolar protein partners, such as RNAPI and UBTF, a key transcription factor required for ribosomal RNA, rRNA) production and TCOF1 (Figure S6C). BD2^mut^ lost the association with RNAPI components and associated proteins such as UBTF upon JQ1 treatment, while (BD1:2)^mut^ recovered a higher level of nucleolar proteins, including TCOF1, in the absence of JQ1 treatment (Figures S6B and S6C).

Since BRD4 actively participates in numerous facets of transcription by RNAPII (reviewed in Bradner et al., 2017), we speculated that BRD3 may participate analogously in rRNA transcription in the nucleolus. To investigate this, we expressed GFP-tagged BRD3 WT or (BD1:2)^mut^ in Flp-In T-REx U-2 OS cells and interrogated their genome-wide distribution using chromatin immunoprecipitation sequencing (ChIP-seq). (BD1:2)^mut^ resulted in genome-wide loss of signal (Figure 6B), and in agreement with previous studies (Anders et al., 2014) we found BRD3 WT at promoters and enhancers, as determined by analysis of ChIP-seq signals and chromatin states from published U-2 OS cell experiments (Walz et al., 2014; Figures 6C, S6D, and S6E). (BD1:2)^mut^ exhibited a loss of ∼84% of the peaks with no robust binding site gains over BRD3 WT (Figures S6D and S6E). This loss could be clearly seen at many BRD3-occupied loci, including BRD4-target loci such as the MYC oncogene that is downregulated by JQ1 treatment (Delmore et al., 2011), as well as CCND2, a G1/S oncogenic cyclin (Dooley et al., 2016; Figure 6C).

In contrast, by comparing BRD3 WT and (BD1:2)^mut^ occupancy at the rDNA gene locus by aligning ChIP-seq reads to a single rDNA repeat, we observed BRD3 WT localization throughout the rDNA intergenic spacer (IGS), while BRD mutations redirected the protein to the transcribed rDNA regions (Figure 6D), displaying high similarity to published TCOF1 ChIP-seq data from HeLa cells (Calo et al., 2018; Figures S6F and S6G). This behavior was phenocopied by JQ1 treatment, which displaced BRD3 from the rDNA IGS region toward the transcribed regions in U-2 OS cells (Figure 6E), consistent with an increased association with TCOF1, UBTF, and RNAPI (Figures S6B and S6C). Taken together, our data suggest that BRD3 may play an unsuspected role in the regulation of rRNA expression.

### BRD3 Impacts rRNA Production and Cell Proliferation

**Figure 7:**
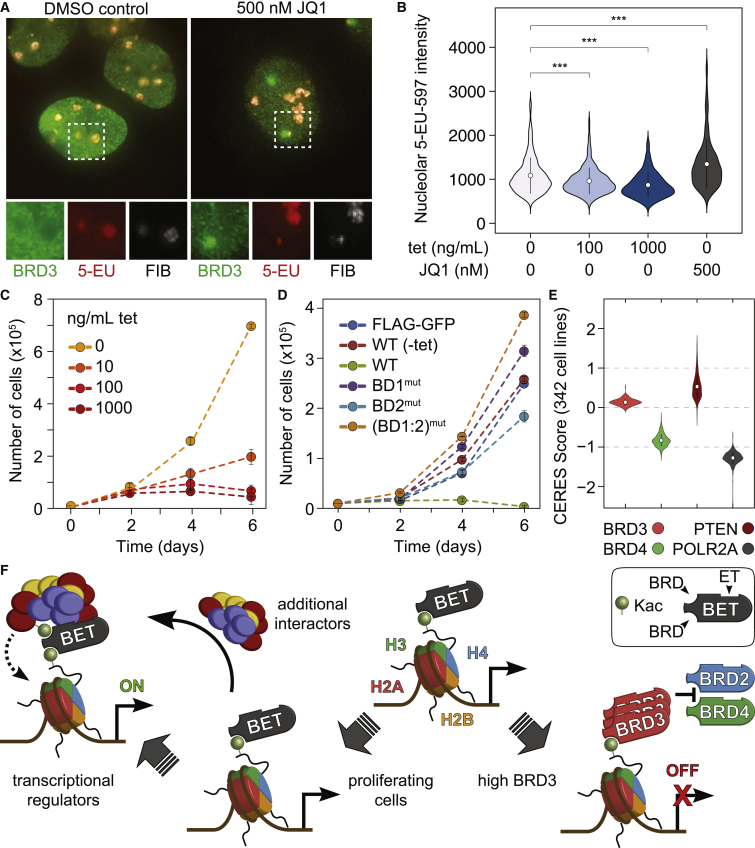
BRD3 Impacts rRNA Production and Cell Proliferation (A) U-2 OS cells were treated with JQ1 or DMSO for 1 hr and then fed 5-EU for 1 hr prior to staining for BRD3, fibrillarin (FIB), and click chemistry to 5-EU-labeled RNA. (B) Quantitative immunofluorescence of U-2 OS cells treated with various concentrations of tetracycline (to titrate BRD3 levels) or JQ1. 5-EU signal overlapping with fibrillarin signal (i.e., nucleolar RNA) was quantified for >400 cells for each experimental condition. ^∗∗∗^p value <0.001, by two-tailed Student’s t test. (C) Cell proliferation assay for U-2 OS treated with tetracycline over 6 days (n = 3). (D) Cell proliferation assay for U-2 OS cells induced with 1 μg/mL tetracycline over 6 days (n = 3). (E) Violin plots of CERES scores showing gene depletion effects from CRISPR-Cas9 loss-of-function screens in 342 cancer cell lines (Meyers et al., 2017). (F) Model of BET protein recruitment functions and the impact of BRD3 overexpression. In proliferating cells, BETs bind to acetylated proteins, including histones through their tandem BRDs, and recruit to chromatin additional transcriptional regulators through other modular domains. High levels of BRD3 antagonize this by decreasing the levels of other BETs, and competing for binding to common loci, in addition to reducing rRNA levels, with a net result of decreasing proliferation. See also Figure S7 and Tables S1 and S2.

To assess BRD3 impact on rRNA expression, we employed a nascent RNA imaging-based 5-ethyl uridine (5-EU) incorporation assay (Larsen et al., 2014). BRD3 speckles co-localizing with the nucleolar marker fibrillarin displayed reduced 5-EU incorporation, compared to regions devoid of BRD3, suggesting that these speckles are refractory to transcription (Figure 7A). Furthermore, BRD3 overexpression reduced 5-EU incorporation in nascent rRNA in a dose-dependent manner (Figure 7B), while prolonged BRD3 overexpression drastically reduced cell proliferation (Figure 7C). Overexpressing BRD3 (BD1:2)^mut^ instead resulted in modest increases in both rRNA production (Figure S7A) and cell proliferation (Figure 7D), in support of the notion that BRD3 repressive functions at the rDNA IGS occur through BRD-dependent interactions. Mining a large dataset of pooled CRISPR screens in cancer cell lines (Meyers et al., 2017) further supported an anti-proliferative role for BRD3. While deletion of BRD4 impedes a cell’s competitive advantage across 342 cell lines profiled, BRD3 depletion caused instead a modest but clear positive gene effect (Figure 7E), suggesting that the anti-proliferative function of BRD3 is not cell type specific.

While it is tempting to speculate that the role of BRD3 in rRNA production may underlie its effects on cell proliferation, our ChIP-seq analysis of WT and (BD1:2)^mut^ cells also suggest that BRD3 may have additional roles on Pol II transcripts, perhaps through competition with BRD4 for common binding sites. We titrated increasing amounts of BRD3 and analyzed the impact on BRD4 recruitment to the *MYC* TSS by qRT-PCR. BRD3 expression reduced BRD4 occupancy at the *MYC* TSS, similarly to JQ1 treatment (Figure S7B), suggesting that competition is possible, at least in an overexpression system. Interpretation of these data is, however, further complicated by the fact that BRD3 expression may also affect the levels of other BETs. Indeed, we find significant occupancy of BRD3 WT at the TSSs of *BRD2* and *BRD4* (Figure S7C). Furthermore, we observed a reduction in BRD2 and BRD4 protein levels following ectopic expression of 3×FLAG-BRD3 in HEK293 cells, while overexpression of any BET reduces the protein levels of the other family members, suggesting a previously unsuspected functional interplay between BET proteins at RNAPII sites (Figures S7D and S7E) that will, however, need to be further substantiated in contexts outside of forced overexpression.

In summary, we uncovered an unsuspected modulation of the BRD3 interactome toward ribosomal biosynthesis genes upon JQ1 treatment, revealing a role for BRD3 in ribosomal biogenesis. We also report an anti-proliferative role for BRD3 that can be attributed in part to its regulation of rRNA expression but may also be associated with regulation of the other BET family proteins and competition for their RNAPII targets.

## Discussion

In this manuscript, we first defined classes of BET interactors with differing quantitative behaviors following pharmacological treatment (decreased, inhibitor independent, and increased), though it remains to be determined where these interactions occur (i.e., on chromatin or in the nucleoplasm).

We show that Kac-XX-Kac sites on non-histone proteins insert both Kac in the BRD cavity as in the previously characterized histone H4 peptide (Filippakopoulos et al., 2012, Morinière et al., 2009), while longer linkers can insert bulky residues in place of the second Kac (e.g., SIRT7 Kac-X_4_-Y motif). Phosphorylation on the longer linker on histone H3 (Kac-X_4_-Kac; the second Kac being outside of the BRD) affects BET binding, potentially linking cellular signaling events to epigenetic circuits. Together with the discovery of binding to a new Kac-Y motif (>16,000 KY motifs are encoded in the human proteome), we suggest that the BET BRDs can contact a larger target space than previously appreciated. While our findings still require testing in a physiological context, they are consistent with a growing list of documented direct associations between BET BRDs and non-histone proteins (reviewed in Fujisawa and Filippakopoulos, 2017). Since we also found that all BET tandem BRDs adopt extended and flexible conformations in solution, it is possible that remote sites within the same or different proteins can be recognized, allowing one BRD to engage a histone while the other simultaneously recruits a non-histone protein through Kac-dependent interactions.

We also systematically characterized two distinct specific ET-interaction motifs for BRD4 (corresponding to motifs mapped to BRD9 and WHSC1/WHSC1L1) that lead to binary interactions for a large portion of the JQ1-insensitive interactome. BRD2 and BRD3 also associated with BRD9 and WHSC1/WHSC1L1 by AP-MS, suggesting that they share association with these SLiMs (Table S2A), and this is supported by analysis by AP-MS of BRD3 WT and ΔET protein (Table S2N). Inspection of published ChIP-seq data further revealed overlap between BRD4 and BRD9 peaks in mouse AML cells (Hohmann et al., 2016, Shi et al., 2013), and between BRD4 and NuRD component CHD4 in mouse embryonic stem cells (ESCs) (Flynn et al., 2016, Luo et al., 2015), suggesting that these proteins may occupy many common loci genome-wide (Figure S7F). Together, this suggests that the ET-recruitment platform may play a role in maintaining transcriptional complexes on chromatin. Since several cancer patient mutations are expected to perturb this fold (Figure S5F), it is possible that loss of ET-mediated interactions contributes to tumor proliferation. Virus proteins have also evolved to hijack this recruitment platform (reviewed in Aydin and Schelhaas, 2016) leading to tight association with the ET domain employing similar motifs to those described here (Crowe et al., 2016).

An initially surprising finding from our data in light of the well-documented role of BRD4 in transcriptional activation is the recovery of negative transcriptional regulators (including NuRD and NELF). While JQ1 treatment generally results in transcriptional repression, we and others have previously reported that subsets of genes are instead upregulated (Lovén et al., 2013, Muhar et al., 2018, Picaud et al., 2013, Picaud et al., 2016, Zuber et al., 2011), suggesting a locus-specific de-repression of transcription by BET inhibition, the mechanisms of which remain to be studied. Our studies also revealed an unsuspected reorganization of BET interactomes following BRD inhibition, which included re-localization of BRD3 to nucleolar speckles where it can dampen rRNA transcription but only in the presence of functional BRDs. We also report a negative role for BRD3 in cell proliferation in our model system that depends on its BRDs, which was corroborated by large-scale genome-wide CRISPR datasets (Meyers et al., 2017). Besides its negative regulation of rRNA transcription, which should contribute to decreased growth, the roles of BRD3 in controlling proliferation are likely more complex, and we present initial evidence for competition with BRD4 for shared targets, as well as an unexpected role of BRD3 levels in regulating the levels of BRD4, and vice versa, at least in the context of overexpressed proteins (schematics in Figure 7F). This has potential implications for cancer etiology, and there are at least some instances in cancer datasets, such as in lung cancer (Szász et al., 2016), where BRD3 and BRD4 levels have opposite associations with patient survival, with both high BRD3 levels and low BRD4 levels showing protective effects (Figure S7G).

Together, our results suggest that a potentially important parameter that should be assessed when evaluating therapeutic inhibition with pan-BET compounds (such as JQ1) is the level of BRD3 expression in relation to other BET proteins. For instance, osteosarcoma HOS cells resistant to JQ1 display increased levels of BRD2 and BRD4 concurrent with a reduction of BRD3 when compared to matched JQ1-sensitive control cells, highlighting that increased BRD3 function does not promote cell growth in this system and that its inhibition may have undesirable consequences (Lamoureux et al., 2014). Finally, it is worth noting that diverse types of cancer cells (Shu et al., 2016) are “addicted” to high BRD4 levels to maintain a pro-proliferative transcriptional program, which could negatively impact the expression levels of BRD2 and BRD3, which in the case of BRD3 could contribute to increased proliferation. We propose that inhibitors that would specifically target, e.g., BRD4 but not BRD3, should be actively pursued, and some initial reports suggest that this may be feasible (Nowak et al., 2018). In this regard, while it has so far proved difficult to develop BRD inhibitors that exhibit high specificity for a single BET, perhaps targeting additional protein-protein interaction domains, such as the ET, may provide alternative ways to modulate BET association and recruitment with transcriptional complexes, thus improving the current therapeutic window.

## STAR★Methods

### Key Resources Table

**Table:** 

REAGENT or RESOURCE	SOURCE	IDENTIFIER
**Antibodies**		

Rabbit anti-BRD2	Sigma	HPA042816; RRID: AB_10794766
Rabbit anti-BRD2	Bethyl	A302-583A; RRID: AB_2034829
Mouse anti-BRD3	Abcam	Ab50818; RRID: AB_868478
Rabbit anti-BRD3	Bethyl	A310-859A
Rabbit anti-BRD4	Bethyl	A301-985; RRID: AB_1576498
Rabbit anti-BRD9	Bethyl	A303-781A; RRID: AB_11218396
Mouse anti-FLAG epitope	Sigma	F3165; RRID: AB_259529
Rabbit anti-GFP	Abcam	Ab290; RRID: AB_303395
Rabbit anti-TCOF1	Sigma	HPA038237; RRID: AB_10670660
Mouse anti-Tubulin	DSHB at University of Iowa	E7; RRID: AB_528499
Rabbit anti-Fibrillarin	Cell Signaling Technologies	2639; RRID: AB_2278087
Rabbit anti-cMyc	Cell Signaling Technologies	5605; RRID: AB_1903938
Mouse anti-HSP90a/b	Santa Cruz Biotechnology	Sc-13119; RRID: AB_675659
Rabbit anti-p21 Waf1/Cip1	Cell Signaling Technologies	2946; RRID: AB_2260325
Mouse anti-SMARCC1 (BAF155)	Santa Cruz Biotechnology	Sc-10756; RRID: AB_2191997
Rabbit anti-MBP epitope	New England BioLabs	E8032S
Rabbit anti-Histone H3	Abcam	Ab1791; RRID: AB_302613
Rabbit anti-Histone H4	EMD Millipore	05-858; RRID: AB_390138
Rabbit anti-TP53	Cell Signaling Technologies	2527; RRID: AB_331211
Rabbit anti-RAD50	Cell Signaling Technologies	3427; RRID: AB_2176936
Rabbit anti-CDK9	Cell Signaling Technologies	2316; RRID: AB_2291505
Rabbit anti-CK2a and CK2a’	David Litchfield lab at the University of Western Ontario	N/A
Rabbit anti-CK2b	David Litchfield lab at the University of Western Ontario	N/A
Donkey anti-rabbit coupled to HRP	GE Healthcare Life Science	NA934
Sheep anti-Mouse coupled to HRP	GE Healthcare Life Science	NA931; RRID: AB_772210
Streptavidin coupled to HRP	GE Healthcare Life Science	GERPN1231-2ML
Goat anti-rabbit coupled to Alexa Fluor 488	Invitrogen	A11008; RRID: AB_143165
Goat anti-rabbit coupled to Alexa Fluor 555	Invitrogen	A-21428; RRID: AB_141784
Goat anti-mouse coupled to Alexa Fluor 488	Invitrogen	A11001
Goat anti-mouse coupled to Alexa Fluor 555	Invitrogen	A21422; RRID: AB_2535844
His-tag Antibody HPR conjugated	Novagen, distributed by Merck-Millipore	71841

**Bacterial and Virus Strains**		

E.coli BL21(DE3)R3-pRARE2	Opher Gileadi	Savitsky et al., 2010
Mach1 cells	Invitrogen	C862003

**Chemicals, Peptides, and Recombinant Proteins**		

magnetic anti-FLAG M2 beads	Sigma-Aldrich	M8823; RRID:AB_2637089
streptavidin-Sepharose bead	GE Healthcare	17-5113-01
MyOne Streptavidin C1 Dynabeads	Invitrogen	65002
Protease Inhibitor Cocktail	Sigma-Aldrich	P8340
Benzonase	EMD	CA80601-766
Trypsin	Sigma-Aldrich	T6567
(+) JQ1	ChemPartner, Shanghai China, http://www.chempartner.com/	Filippakopoulos et al., 2010
(-) JQ1	ChemPartner, Shanghai China, http://www.chempartner.com/	Filippakopoulos et al., 2010
Dynabeads Protein A for Immunoprecipitation	Invitrogen	10002D
Peptides used for biophysical studies	This study	Table S1E

**Critical Commercial Assays**		

Click-iT RNA Alexa Fluor 594 imaging kit	Molecular Probes	C10330

**Deposited Data**		

Original imaging data (SPOT arrays, microscopy, and western blots) presented in this study	This paper; Mendeley Data	https://doi.org/10.17632/xtb4mkvf8f.1
Original imaging data (western blots)	This paper; Mendeley Data	https://doi.org/10.17632/jb4jjxsbb7.1
Original imaging data (Live-cell imaging)	This paper; Mendeley Data	https://dx.doi.org/10.17632/fzvwgpjx88.1
MS data of BET JQ1 AP-MS time-course	https://massive.ucsd.edu/ProteoSAFe/static/massive.jsp	MSV000081006
MS data of BRD3 mutant BioID	https://massive.ucsd.edu/ProteoSAFe/static/massive.jsp	MSV000081001
MS data of BRD9 fragment AP-MS	https://massive.ucsd.edu/ProteoSAFe/static/massive.jsp	MSV000080981
MS data of rBRD4 domain pull-down dataset	https://massive.ucsd.edu/ProteoSAFe/static/massive.jsp	MSV000080986
MS data of BET ΔET AP-MS dataset	https://massive.ucsd.edu/ProteoSAFe/static/massive.jsp	MSV000080988
MS data of endogenous BET IP-MS HEK293	https://massive.ucsd.edu/ProteoSAFe/static/massive.jsp	MSV000082857
MS data of endogenous BET IP-MS K562	https://massive.ucsd.edu/ProteoSAFe/static/massive.jsp	MSV000082859
AP-MS and BioID dataset in searchable format	https://prohits-web.lunenfeld.ca/	Project 40 (BET rewiring)
Crystal structure of BRD4/BD1 with an H3 (K9ac/K14ac) peptide	This paper; http://www.pdb.org	PDB: 5NNC
Crystal structure of BRD4/BD1 with an H3 (K9ac/pS10/K14ac) peptide	This paper; http://www.pdb.org	PDB: 5NND
Crystal structure of BRD4/BD1 with a TOP2A (K1201ac/K1204ac) peptide	This paper; http://www.pdb.org	PDB: 5NNE
Crystal structure of BRD4/BD1 with a BAZ1B (K221ac) peptide	This paper; http://www.pdb.org	PDB: 5NNF
Crystal structure of BRD4/BD1 with an SRPK1 (K585ac) peptide	This paper; http://www.pdb.org	PDB: 5NNG
Crystal structure of BRD4/BD1 with an ATRX (K1030ac/K1033ac) peptide	This paper; http://www.pdb.org	PDB: 6G0O
Crystal structure of BRD4/BD1 with an E2F1 (K117ac/K120ac) peptide	This paper; http://www.pdb.org	PDB: 6G0P
Crystal structure of BRD4/BD1 with a GATA1 (K312ac/K315ac) peptide	This paper; http://www.pdb.org	PDB: 6G0Q
Crystal structure of BRD4/BD1 with a POL2RA (K775ac/K778ac) peptide	This paper; http://www.pdb.org	PDB: 6G0R
Crystal structure of BRD4/BD1 with a SIRT7 (K272ac/K275ac) peptide	This paper; http://www.pdb.org	PDB: 6G0S
Small-angle scattering data and models of tandem BRD2 BD1/BD2	This paper, https://www.sasbdb.org	SASDCT2
Small-angle scattering data and models of tandem BRD3 BD1/BD2	This paper, https://www.sasbdb.org	SASDCS2
Small-angle scattering data and models of tandem BRD4 BD1/BD2	This paper, https://www.sasbdb.org	SASDCR2
Small-angle scattering data and models of tandem BRDT BD1/BD2	This paper, https://www.sasbdb.org	SASDCU2
ChIP-seq data of BRD3 WT and BRD3 BD(1:2)mut	This paper, https://www.ebi.ac.uk/arrayexpress	E-MTAB-5670
ChIP-seq data in U2-OS for H3K27ac, H3K4me3, and H3K4me1	https://www.ncbi.nlm.nih.gov/geo/	GEO: GSE44672
ChIP-seq of TCOF1 in HeLa	https://www.ncbi.nlm.nih.gov/geo/	GEO: GSE89420
BRD4 ChIP-seq in mouse embryonic stem cells (mESCs)	https://www.ncbi.nlm.nih.gov/geo/	GEO: GSE69140
CHD4 ChIP-seq in mouse embryonic stem cells (mESCs)	https://www.ncbi.nlm.nih.gov/geo/	GEO: GSE61188
BRD4 ChIP-seq in mouse leukemic cells	https://www.ncbi.nlm.nih.gov/geo/	GEO: GSE52279
BRD9 ChIP-seq in mouse leukemic cells	https://www.ncbi.nlm.nih.gov/geo/	GEO: GSE79360
DNaseI peaks from Encode	http://hgdownload.soe.ucsc.edu/goldenPath/mm9/encodeDCC/wgEncodePsuDnase/	wgEncodePsuDnaseG1eS129ME0PkRep1.narrowPeak.gz
Encode blacklist regions (hg19)	https://sites.google.com/site/anshulkundaje/projects/blacklists	hg19/GRCh37
CERES dataset	Meyers et al., 2017	Table S3
RefSeq database (v. 57 (01/30/2013))	NCBI	N/A
Common mass spectrometry contaminants	Max Planck Institute	http://141.61.102.106:8080/share.cgi?ssid=0f2gfuB
Global Proteome Machine	Beavis, 2006	https://www.thegpm.org/crap/index.html
BioGRID (version 3.4.157 (01/25/2018))	https://thebiogrid.org/	RRID: SCR_007393
PhosphoSitePlus: Protein Modification Site (June 2016 version)	https://www.phosphosite.org/	RRID: SCR_001837
Uniprot/SwissProt (April 2017 version)	https://www.uniprot.org/	RRID: SCR_002380

**Experimental Models: Cell Lines**		

HEK293 Flp-In T-REx	Invitrogen	R780-07
U-2 OS Flp-In T-REx	Dr. Patrick Meraldi from ETH Zurich	LTRI cell line ID C971
HeLa	ATCC	CCL-2
K562	Mark Minden	CCL-243

**Oligonucleotides**		

ChIP-qPCR primers	This study	Table S1F
ON-TARGETplus siRNA targeting BRD3 (1)	Dharmacon	J-004936-05-0002
ON-TARGETplus siRNA targeting BRD3 (2)	Dharmacon	J-004936-06-0002
ON-TARGETplus siRNA targeting BRD3 (3)	Dharmacon	J-004936-07-0002
ON-TARGETplus siRNA targeting BRD3 (4)	Dharmacon	J-004936-08-0002
ON-TARGETplus siRNA targeting TCOF1 (1)	Dharmacon	J-012550-05-0002
ON-TARGETplus siRNA targeting TCOF1 (2)	Dharmacon	J-012550-06-0002
ON-TARGETplus siRNA targeting TCOF1 (3)	Dharmacon	J-012550-07-0002
ON-TARGETplus siRNA targeting TCOF1 (4)	Dharmacon	J-012550-08-0002
Primers employed for cloning	This study	Table S1D

**Recombinant DNA**		

pDEST 5′ 3x-FLAG-pcDNA5-FRT-TO	Lambert et al., 2015	LTRI vector ID V4978
pDEST 5′ BirA^∗^-FLAG-pcDNA5-FRT-TO	Lambert et al., 2015	LTRI vector ID V8164
pDEST 5′ eGFP-pcDNA5-FRT-TO	Lambert et al., 2015	LTRI vector ID V4874
pDESTpMal_c2x-v2	N/A	LTRI vector ID V8324
Human BRD4 (NP_490597.1) cDNA codon optimized	FivePrime	N/A
Mouse Brd9 (NP_001019679.2) cDNA	MGC (Mammalian Gene Collection)	LTRI vector ID V7333
Human BRD3 (NP_031397.1) cDNA	Picaud et al., 2013	N/A
Human WHSC1L1 (NP _075447.1) cDNA	MGC (Mammalian Gene Collection)	BC101717; IMAGE ID:8069223
Human ZNF592 (NP_055445.2) cDNA	MGC (Mammalian Gene Collection)	BC112232; IMAGE ID:8327700
pNIC28-Bsa4	Savitsky et al., 2010	N/A

**Software and Algorithms**		

MS data storage and analysis: ProHits (v.4.0)	Liu et al., 2016	http://prohitsms.comProhits_download/list.php
ProteoWizard (v3.0.4468)	http://proteowizard.sourceforge.net/	N/A
AB SCIEX MS Data Converter (V1.3 beta)		N/A
Mascot (version 2.3.02)	http://www.matrixscience.com	RRID: SCR_014322
Comet (version 2012.02 rev.0)	Eng et al., 2013	http://comet-ms.sourceforge.net/
MS-GFDB (Beta version 1.0072 (6/30/2014))	Kim et al., 2010	N/A
G:Profiler	https://biit.cs.ut.ee/gprofiler/	RRID: SCR_006809
MS Data: Independent Acquisition analysis: MSPLIT-DIA (v.1.0)	Wang et al., 2015	http://proteomics.ucsd.edu/software-tools/msplit-dia/
MS data: Significance Analysis of INTeractome analysis (SAINT v.3.3)	Choi et al., 2011	http://saint-apms.sourceforge.net/
MS data visualization: ProHits-Viz	Knight et al., 2017	https://prohits-viz.lunenfeld.ca/
Network visualization: Cytoscape (v.3.5.1)	https://cytoscape.org/	RRID: SCR_003032
Mass Hunter WorkStation Qualitative Analysis (v.B.06.00)	Agilent Technologies, Palo Alto, CA	https://www.agilent.com/en/products/software-informatics/masshunter-suite/masshunter-qualitative-analysis-gcms
Image analysis: MATLAB scripts	This study	N/A
WebLogo	http://weblogo.berkeley.edu	RRID: SCR_010236
Harmony analysis software (v.4.1)	PerkinElmer	http://www.perkinelmer.com/product/harmony-4-8-office-hh17000001
ImageJ (FiJi v.1.51w)	https://imagej.net/Fiji/Downloads	RRID: SCR_003070
Kodak 1D Scientific Imaging System (v.3.6.2)	Kodak	N/A
INTAVIS RSi Spotter MultiPep (v.4.0.34)	INTAVIS Bioanalytical Instruments	https://intavis.com/
OriginPro (v.7.5 & v.9.4)	https://www.originlab.com/Origin	RRID: SCR_014212
SEDFIT (v.15.01)	Schuck, 2000	http://www.analyticalultracentrifugation.com/download.htm
Sednterp (v.1.08)	http://www.jphilo.mailway.com/download.htm	RRID: SCR_016253
MACS2 (v.2.1.0.20151222)	Liu Lab	https://github.com/taoliu/MACS/
Ngs.plot (v.2.6.3)	https://github.com/shenlab-sinai/ngsplot	RRID: SCR_011795
seqMINER (v.1.3.3)	https://sourceforge.net/projects/seqminer/	RRID: SCR_013020
softWoRx (v.5.0)	Applied Precision	https://www.bioz.com/search/applied%20precision%20softworx%20imaging%20software
deepTools (v.2.0)	https://deeptools.readthedocs.io/en/develop/	RRID: SCR_016366
GAT	Heger et al., 2013	https://github.com/AndreasHeger/gat
Bedtools (2.26)	Quinlan laboratory	https://bedtools.readthedocs.io/en/latest/
Bowtie 2 (v.2.2.3.4.1)	http://bowtie-bio.sourceforge.net/bowtie2/index.shtml	RRID: SCR_016368
Trimmomatic (v.0.36)	http://www.usadellab.org/cms/?page=trimmomatic	RRID: SCR_011848
Bwa (v.0.7.8)	Li and Durbin, 2009	http://bio-bwa.sourceforge.net/RRID:SCR_010910
Sushi (v.1.16.0)	Phanstiel et al., 2014	https://www.bioconductor.org/packages/release/bioc/html/Sushi.html
SRA toolkit (v.2.9.0)	NCBI	https://www.ncbi.nlm.nih.gov/sra/docs/toolkitsoft/
ChromHMM (v.1.17)	Ernst and Kellis, 2012	http://compbio.mit.edu/ChromHMM/
Expasy ProtParam	https://www.expasy.org/tools/protparam.html	RRID: SCR_012880
R Project for Statistical Computing (v.3.5)	https://www.r-project.org/	RRID: SCR_001905
PyMOL, (v.1.8)	https://pymol.org/2/	RRID: SCR_000305
XDS Program Package (built 20180126)	Kabsch, 2010	http://xds.mpimf-heidelberg.mpg.de/;RRID: SCR_015652
CCP4 Suite (v.6.5 & v.7.0)	Winn et al., 2011	RRID: SCR_007255
ARP/wARP (v.7.0)	Perrakis et al., 1999	http://www.embl-hamburg.de/ARP/
Coot (v.0.8)	https://www2.mrc-lmb.cam.ac.uk/personal/pemsley/coot/	RRID: SCR_014222
MolProbity (v.3.0)	http://molprobity.biochem.duke.edu/	RRID: SCR_014226
Refmac (v.5.1)	http://www.ccp4.ac.uk/html/refmac5/description.html	RRID: SCR_014225
DAWN Suite (v.2.10.0)	Basham et al., 2015	https://dawnsci.org/downloads/
ATSAS Suite (v.2.8)	Franke et al., 2017	https://www.embl-hamburg.de/biosaxs/software.html
ScÅtter (v.3.1)	Robert Rambo at the Diamond Light Source (Didcot, UK)	http://www.bioisis.net/tutorial/9

### Contact for Reagent and Resource Sharing

Further information and requests for resources and reagents should be directed to the Lead Contact, Panagis Filippakopoulos (panagis.filippakopoulos@sgc.ox.ac.uk).

### Experimental Model and Subject Details

#### Cell Lines

Flp-In T-REx HEK293 cells (female; Invitrogen), Flp-In T-REx U-2 OS (female; P. Meraldi) or HeLa (female; ATCC) cells were grown in DMEM + 10% FBS (or 5% FBS and 5% calf serum for Flp-In T-REx HEK293 cells) containing penicillin and streptomycin. K562 cells (a kind gift from Mark Minden) were grown in suspension in RPMI + 10% FBS containing penicillin and streptomycin to a concentration of 500,000 cells/mL (in a 175 cm^2^ flask containing 60 mL of medium). Flp-In T-REx U-2 OS (female; P. Meraldi) for the chromatin localization assay were grown and maintained in DMEM, with GlutaMAX supplement, supplemented with 10% heat-inactivated fetal bovine serum, 100 U/mL penicillin-streptomycin; clones for the LacO/LacR assay were grown in 1 μg/mL puromycin. Cells were grown at 37°C in 5% (HEK293, HeLa, K562) or 10% (U-2 OS) CO_2_; parental stocks are periodically checked for mycoplasma contamination, but have not been independently authenticated. *E. coli* BL21(DE3)-R3-pRARE2 cells (a phage-resistant derivative of the BL21(DE3) strain), with a pRARE plasmid encoding rare codon tRNAs were cultured in 2 × lysogeny broth (LB) supplemented with 50 μg/mL kanamycin and 34 μg/mL chloramphenicol at 37°C. Mach1 cells (Invitrogen, cat# C862003) were cultured in 2 × lysogeny broth (LB) supplemented with 50 μg/mL kanamycin at 37°C.

### Method Details

#### BET Inhibitors

The thienodiazepines (+)-JQ1 and (-)-JQ1 were synthesized as previously described (Filippakopoulos et al., 2010).

#### Cloning

Constructs for the genes of interest were generated via Gateway cloning into pDEST 5′ 3×FLAG-pcDNA5-FRT-TO, pDEST 5′ eGFP-pcDNA5-FRT-TO or pDEST 5′ BirA^∗^-FLAG-pcDNA5-FRT-TO. Details of all entry clones and destination vectors used in this study can be found in Tables S1A and S1B.

cDNAs encoding human BRD4 (National Center for Biotechnology Information (NCBI) accession number NP_490597.1; first bromodomain (BD1): N44-E168; second bromodomain (BD2): D334-E460; and BRD4 extra-terminal domain (ET): A589-R676) (from synthetic codon optimized clone (FivePrime)) and mouse Brd9 (NCBI accession number NP_001019679.2, M1-A242) N-terminal region, were amplified by polymerase chain reaction (PCR) in the presence of Herculase II fusion DNA polymerase (Agilent Technologies). PCR products were purified (QIAquick PCR Purification Kit, QIAGEN UK) and further sub-cloned into a pET28-derived expression vector, pNIC28-Bsa4 using ligation-independent cloning (Savitsky et al., 2010). This vector includes sites for ligation-independent cloning and a Tobacco Etch Virus (TEV)-cleavable N-terminal His6-tag (extension MHHHHHHSSGVDLGTENLYFQ^∗^SM-). After digestion with TEV protease, the protein retains an additional serine and methionine on the N terminus. The constructs were transformed into competent Mach1 cells (Invitrogen, UK) to yield the final plasmid DNA.

#### Mutagenesis

The BRD4/ET mutant (BRD4/ET^mut^, D650K/E651K/E653K/D655K) was cloned using a two-step PCR. First, the C-terminal part was amplified using a long forward primer bearing the mutations and the BRD4/ET reverse primer. The PCR product was then purified from an agarose gel and used as a degenerated primer during the amplification of the full-length PCR fragment, in combination with the BRD4/ET forward primer. Mutations which impede binding to a first (N116F) or second (N391F) bromodomains were introduced into the full-length BRD3 Gateway entry clone using a 15-cycle QuikChange II PCR protocol (Agilent). Mutations or deletions which impede binding to the BRD4 ET domain were introduced into full-length BRD9 (Uniprot: Q9H8M2; BRD9^mut^: K29A/V31A/K33A; BRD9^del^: deletion of D18-G36); WHSC1L1 (Uniprot: Q9BZ95; WHSC1L1^mut^: K154A/L155A/K156A; WHSC1L1^del^: deletion of V143-I161); ZNF592 (Uniprot: Q92610; ZNF592^mut^: K374A/V375A/R376A; ZNF592^del^: deletion of V364-T379) in Gateway entry clones using the same QuikChange II protocol.

#### Cell Line Generation

Bait proteins of interest were stably expressed in Flp-In T-REx HEK293 or Flp-In T-REx U-2 OS cells as described (Lambert et al., 2014). Parental Flp-In T-REx HEK293 cells, and stable cells expressing BirA^∗^-FLAG fused either to a green fluorescent protein (GFP) or to a nuclear localization sequence (NLS) were used as negative controls for the BioID experiments and processed in parallel to the bait proteins. Flp-In T-REx HEK293 cells, expressing NLS-BirA^∗^ fused to a FLAG tag were used as negative controls for AP-MS experiments and were processed in parallel to the bait-expressing cell lines. Stable cell lines were selectively grown in the presence of 200 μg/mL hygromycin up to 80% confluence before expression was induced via 1 μg/mL tetracycline for 24 h (unless otherwise indicated) and the cells were harvested. For BioID experiments, two 150-mm plates were induced with tetracycline and treated with 50 μM biotin for 24 h before harvesting. Cells were pelleted at low speed, washed with ice-cold phosphate-buffered saline (PBS) and frozen at −80°C until purification.

The U-2 OS-LacO cell line was generated using Flp-In T-REx U2-OS cells following a previously described protocol (Roukos et al., 2014). The LacO array (256x repeats) was digested from the Lac-I-SceI-Tet plasmid (Addgene, #17655) with Xhol. The linearized LacO array together with a pSELECT-puro plasmid encoding the puromycin resistance gene (Invivogen, cat. no. psetp-mcs) were co-transfected into U-2 OS cells using FuGENE6 (Promega, cat.# E2692) according to the manufacturer’s protocol. After 48 h of transfection, cells were re-plated onto a 15 cm tissue culture dish and selected with 1 μg/mL puromycin for 10 d. Individual colonies of puromycin-resistant clones were grown and maintained in DMEM, with GlutaMAX supplement (cat.# 10566-016, GIBCO), supplemented with 10% heat-inactivated fetal bovine serum (FBS, Sigma, cat.# F4135), 100 U/mL penicillin-streptomycin (GIBCO, cat.# 15140122), and 1 μg/mL puromycin (Invivogen, cat.# ant-pr-1). Integration of the LacO array was confirmed by exogenously expressing mCherry-LacR-NLS and checking for bright mCherry-dots.

#### FLAG Affinity Purification Using a Chromatin Optimized Protocol

To identify interactions for BET proteins that are either occurring on chromatin, the nucleoplasm or in other locations, we adapted the chromatin-optimized FLAG AP-MS protocol from (Lambert et al., 2014) with slight modifications. Essentially, this protocol incorporates DNA shearing by sonication and nucleases to solubilize protein complexes associated with DNA while largely maintaining protein-protein interactions. This protocol was initially optimized to enable the solubilisation of histones from cell pellets alongside the recovery of their interaction partners, including BRD2 and BRD4 (Lambert et al., 2014). These protocols were also tested on the recovery of interaction partners for FLAG-tagged BRD2 at the onset of the project, revealing a shift from proteins expected to be soluble (in the cytosol or nucleoplasm) to proteins known to be associated to chromatin when employing the optimized protocol instead of a more standard AP-MS protocol (data not shown). Stable cells from two 150-mm plates were pelleted, frozen, and lysed in 1.5 mL ice-cold low salt lysis buffer [50 mM HEPES-NaOH pH 8.0, 100 mM KCl, 2 mM EDTA. 0.1% NP40, and 10% glycerol with 1 mM PMSF, 1 mM DTT and Sigma-Aldrich protease inhibitor cocktail (P8340, 1:500) added immediately prior to processing]. To aid with lysis, the cells were frozen on dry ice, thawed in a 37°C water bath, and returned to ice. The samples were sonicated using a QSONICA 125W sonicator equipped with 1/8” probe at 4°C using three 10 s bursts with 2 s pauses at 35% amplitude. Benzonase (100 units) was added and the lysates were incubated at 4°C for 1 h with rotation. The lysates were centrifuged at 20,817 × *g* for 20 min at 4°C and the supernatant was added to tubes containing 25 μL of a 50% magnetic anti-FLAG M2 beads (Sigma-Aldrich, M8823) slurry prewashed in lysis buffer. FLAG immunoprecipitation was allowed to proceed at 4°C for 2 h with rotation. Beads were pelleted by centrifugation (1000 rpm for 1 min) and magnetized, and the unbound lysate was aspirated and kept for analysis. The beads were demagnetized, washed with 1 mL lysis buffer, and remagnetized to aspirate the wash buffer. The beads were then washed with 1 mL of 20 mM Tris–HCl (pH 8.0) containing 2 mM CaCl_2_ and any excess wash buffer was removed by centrifuging the beads, magnetizing, and pipetting off the remaining liquid. The now-dry magnetic beads were removed from the magnet and resuspended in 7.5 μL of 20 mM Tris–HCl (pH 8.0) containing 750 ng of trypsin (Sigma-Aldrich, T7575) and the mixture was incubated overnight at 37°C with agitation. After the initial incubation, the beads were magnetized and the supernatant was transferred to a fresh tube. Another 250 ng of trypsin was added to the mixture and further digested, without agitation, for 3–4 h. The sample was acidified with formic acid to a final concentration of 2% and the tryptic digests were stored at −40°C until mass spectrometry analysis.

#### Endogenous Immunoprecipitation for Mass Spectrometry

Untransfected Flp-In T-REx HEK293 cells (Invitrogen: R780-07 – passage 6) were grown in DMEM + 10% FBS (or 5% FBS and 5% calf serum) containing penicillin and streptomycin until a confluence of 85% was reached. JQ1 (or DMSO) was added at a final concentration of 500 nM for 1 h, prior to harvest by scraping and centrifugation. Cell pellets were washed once with ice-cold PBS, and were stored dry at −80°C. K562 cells (a kind gift from Mark Minden) were grown in suspension in RPMI + 10% FBS containing penicillin and streptomycin to a concentration of 500,000 cells/mL (in a 175 cm^2^ flask containing 60 mL of medium) prior to treatment with JQ1 as above. Cells were harvested by centrifugation (400 *g* for 5 min at 4°C), rinsed in 1 mL ice-cold PBS, and centrifuged again. The dry pellets were stored at −80°C.

Dry cell pellets were weighed and re-suspended in ice-cold lysis buffer at a 1:4 pellet weight:volume ratio. The lysis buffer contains 50 mM HEPES-NaOH pH 8.0, 100 mM KCl, 2 mM EDTA. 0.1% NP40, and 10% glycerol with 1 mM PMSF, 1 mM DTT and Sigma-Aldrich protease inhibitor cocktail (P8340, 1:500) added immediately prior to processing. To aid with lysis, the cells were frozen on dry ice, thawed in a 37°C water bath, and nutated for 10 min at 4°C before being returned to ice. As for the FLAG AP-MS dataset, the chromatin optimized protocol from (Lambert et al., 2014) was adopted with minor modifications to gently solubilize DNA-associated protein complexes. The samples were sonicated using a QSONICA 125W sonicator equipped with 1/8” probe at 4°C using three 10 s bursts with 3 s pauses at 33% amplitude. Benzonase (100 units) was added and the lysates were incubated at 4°C for 30 min with rotation. The lysates were centrifuged at 20,817 × *g* for 20 min at 4°C.

To prepare beads for immunoprecipitation, antibodies to endogenous BRD2, BRD3 and BRD4 (0.5 μg per immunoprecipitation) were coupled to pre-washed magnetic beads (Dynabeads Protein A for Immunoprecipitation, Invitrogen; 10 μL of a 50:50% slurry) for 2 h in PBS on a nutator (at 4°C) [Note that the optimal amount of antibody needed for the depletion of the BET proteins from the cell lysate was assessed by immunoprecipitation coupled to western blot from an HEK293 cell lysate prior to the mass spectrometry experiment]. Beads washed three times in lysis buffer (500 μL) before they were added to the lysate.

The lysate supernatants were added to the prepared beads (equivalent cell pellet weights were used for each immunoprecipitation across each cell line). Immunoprecipitations were allowed to proceed at 4°C for 4 h with rotation. Beads were pelleted by centrifugation (1000 rpm for 1 min) and magnetized. The beads were demagnetized, washed with 1 mL lysis buffer, and re-magnetized to aspirate the wash buffer. The beads were then washed with 1 mL of 50 mM ammonium bicarbonate pH 8. The now-dry magnetic beads were removed from the magnet and re-suspended in 7.5 μL of 20 mM Tris–HCl (pH 8.0) containing 1 μg of trypsin (Sigma-Aldrich, T7575) and the mixture was incubated overnight at 37°C with agitation. After the initial incubation, the beads were magnetized and the supernatant was transferred to a fresh tube. Another 250 ng of trypsin was added to the mixture and further digested, without agitation, for 3–4 h. The sample was acidified with formic acid to a final concentration of 2% and the tryptic digests were set to dry using the speedvac. This was followed by a peptide clean-up using C_18_ Stage Tips (Thermo, SP301) and then stored at −40°C until mass spectrometry analysis.

#### Proximity-Dependent Biotinylation Mass Spectrometry

Cell pellets from two 150-mm plates were pelleted, frozen, and thawed in 1.5 mL ice cold RIPA buffer containing 50 mM Tris-HCl (pH 7.5), 150 mM NaCl, 1% NP-40, 1 mM EDTA, 1 mM EGTA, 0.1% SDS and 0.5% sodium deoxcycholate. PMSF (1 mM), DTT (1 mM) and Sigma-Aldrich protease inhibitor cocktail (P8340, 1:500) were added immediately before use. The lysates were sonicated using a QSONICA 125W sonicator equipped with 1/8” probe, treated with benzonase and centrifuged as described in the FLAG AP-MS section. For each sample, 60 μL of streptavidin-Sepharose bead slurry (GE Healthcare, Cat 17-5113-01) was pre-washed three times with 1 mL of lysis buffer by pelleting the beads with gentle centrifugation and aspirating off the supernatant before adding the next wash. Biotinylated proteins were captured on pre-washed streptavidin beads for 3 h at 4°C with rotation. The beads were gently pelleted and then washed twice with 1 mL RIPA buffer and three times with 1 mL 50 mM ammonium bicarbonate (pH 8.0). Following the final wash, the beads were pelleted and any excess liquid was aspirated off. Beads were re-suspended in 100 μL of 50 mM ammonium bicarbonate, and 1 μg of trypsin solution was added. The samples were incubated overnight at 37°C with rotation and then an additional 1 μg of trypsin was added, followed by further incubation for 2–4 h. The beads were pelleted and the supernatant was transferred to a fresh tube. The beads were rinsed twice with 100 μL HPLC-grade water, and the wash fraction was combined with the supernatant. The peptide solution was acidified with 50% formic acid to a final concentration of 2% and the samples were placed in a Speedvac to dry. Tryptic peptides were re-suspended in 25 μL 5% formic acid and stored at −80°C until mass spectrometry analysis.

#### Recombinant Domain Pull-Downs for Mass Spectrometry

To a frozen HEK293 cell pellet of ∼2 × 10^7^ cells, 1.5 mL of ice-cold high salt lysis buffer [50 mM HEPES-NaOH (pH 8.0), 500 mM KCl, 2 mM EDTA, 0.1% NP-40, 10% glycerol, 1 mM PMSF, 1 mM dithiothreitol, and Sigma-Aldrich Protease Inhibitor Cocktail (P8340, 1:500)] was added to gently re-suspend the frozen pellet. Samples were subjected to a freeze/thaw cycle on dry ice until completely frozen (5 to 10 min) and then transferred to a 37°C water bath with agitation until only a small amount of ice remained. Samples were sonicated using a QSONICA 125W sonicator equipped with 1/8” probe and treated with benzonase as per the AP-MS protocol. The resulting samples were centrifuged at 14,000 rpm (20,873 × *g*) for 20 min at 4°C, and the supernatants were transferred to fresh 2-mL tubes. Biotinylated recombinant BRD4 fragment [25 μg conjugated to 30 μL of MyOne Streptavidin C1 Dynabeads (65002; Invitrogen, Thermo Fisher Scientific per sample for at least 1 h in PBS) were washed as a pool with lysis buffer. A volume representing the initial 30 μL of beads was subsequently aliquoted for each purification and an equal amount of cell lysates was added to each aliquot. The mixture was incubated for 2 h at 4°C with gentle agitation (nutator) with or without competition with 5 nmol of JQ1 (for recombinant bromodomains only). Beads were pelleted by centrifugation (1000 rpm for 5 s), and tubes were placed on a cold magnetic rack (on ice) to collect the beads on the side of the tubes. The supernatant was removed slowly with a pipette, and the beads were washed once with 1 mL of cold lysis buffer containing 500 mM KCl and twice more with 1 mL of cold lysis buffer containing a reduced salt concentration (100 mM KCl). The beads were then transferred to a fresh 1.7-mL tube using 1 mL of 20 mM Tris-HCl (pH 8.0) and 2 mM CaCl_2_. After the last wash, the samples were quickly centrifuged, and the last drops of liquid were removed with a fine pipette. The samples were re-suspended in 7.5 μL of 20 mM Tris-HCl (pH 8.0) containing 750 ng of trypsin (Sigma-Aldrich, T7575), and the suspension was incubated at 37°C with agitation overnight on an angled rotating wheel (∼15 h). After this first incubation, samples were quickly centrifuged and then magnetized, and the supernatants were transferred to a fresh tube. Another 250 ng of trypsin was added to the digested proteins [in 2.5 μL of 20 mM Tris-HCl (pH 8.0)], and the resulting sample was incubated at 37°C for 3–4 h without agitation. Formic acid was then added to a final concentration of 2% (from 50% stock solution) and the samples were stored at −80°C.

#### Experimental Design for Mass Spectrometry Experiments

For each bait, two biological replicates were processed independently. These were analyzed alongside negative controls in each batch of samples processed. For AP-MS, cell lines expressing a 3×FLAG-GFP tag construct or no bait (i.e., empty cell lines) were used. For BioID, cell lines expressing a BirA^∗^-FLAG-GFP construct, a BirA^∗^-NLS-FLAG construct or no bait (i.e., empty cell line) were used. These control cell lines were grown in parallel to those expressing baits and treated in the same manner (24 hr tetracycline induction, etc.). To minimize carry-over issues during liquid chromatography, extensive washes were performed between each sample (see details for each instrumentation type); and the order of sample acquisition on the mass spectrometer was also reversed for the second biological replicates to avoid systematic bias.

#### Preparation of HPLC Columns for Mass Spectrometry

A spray tip was formed on a fused silica capillary column (0.75 μm ID, 350 μm OD) using a laser puller (program = 4; heat = 280, FIL = 0, VEL = 18, DEL = 200). C_18_ reversed-phase material in MeOH (10–12 cm; Reprosil-Pur 120 C_18_-AQ, 3 μm; Dr.Maisch HPLC GmbH, Germany) was packed in the column with a pressure bomb. The column was then equilibrated in buffer A prior to sample loading.

#### Mass Spectrometry Acquisition Using TripleTOF Mass Spectrometers

Each sample (5 μL) was directly loaded at 400 nL/min onto an equilibrated HPLC column. The peptides were eluted from the column over a 90 min gradient generated by a NanoLC-Ultra 1D plus (Eksigent, Dublin CA) nano-pump and analyzed on a TripleTOF 5600 instrument (AB SCIEX, Concord, Ontario, Canada). The gradient was delivered at 200 nL/min starting from 2% acetonitrile with 0.1% formic acid to 35% acetonitrile with 0.1% formic acid over 90 min followed by a 15 min clean-up at 80% acetonitrile with 0.1% formic acid, and a 15 min equilibration period back to 2% acetonitrile with 0.1% formic acid, for a total of 120 min. To minimize carryover between each sample, the analytical column was washed for 3 h by running an alternating sawtooth gradient from 35% acetonitrile with 0.1% formic acid to 80% acetonitrile with 0.1% formic acid, holding each gradient concentration for 5 min. Analytical column and instrument performance were verified after each sample by loading 30 fmol bovine serum albumin (BSA) tryptic peptide standard (Michrom Bioresources Fremont, CA) with 60 fmol α-casein tryptic digest and running a short 30 min gradient. TOF MS calibration was performed on BSA reference ions before running the next sample to adjust for mass drift and verify peak intensity. The instrument method was set to data dependent acquisition (DDA) mode, which consisted of one 250 ms (ms) MS1 TOF survey scan from 400–1300 Da followed by 20 100 ms MS2 candidate ion scans from 100–2000 Da in high sensitivity mode. Only ions with a charge of 2+ to 4+ that exceeded a threshold of 200 cps were selected for MS2, and former precursors were excluded for 10 s after one occurrence. For the analysis of the JQ1 time course, half of the sample was analyzed by DDA as above, and the other half was analyzed (using the same loading and HPLC gradient conditions) by data independent acquisition (SWATH). In that case, acquisition consisted of one 50 ms MS1 scan followed by 32 × 25 a.m.u. isolation windows covering the mass range of 400–1250 a.m.u. (cycle time of 3.25 s); an overlap of 1 Da between SWATH was preselected. The collision energy for each window was set independently as defined by CE = 0.06 × m/z + 4, where m/z is the center of each window, with a spread of 15 eV performed linearly across the accumulation time.

#### Mass Spectrometry Acquisition Using LTQ-Orbitrap Mass Spectrometers

Each sample (5 μL) was directly loaded at 400 nL/min onto an equilibrated HPLC column. The peptides were eluted from the column by a gradient generated by a NanoLC-Ultra 1D plus (Eksigent, Dublin CA) nano-pump and analyzed on a LTQ-Orbitrap Elite (Thermo Electron) equipped with a nanoelectrospray ion source (Proxeon, Thermo Scientific). The LTQ-Orbitrap Elite instrument under Xcalibur 2.0 was operated in the data dependent mode to automatically switch between MS and up to 10 subsequent MS/MS acquisitions. Buffer A was 99.9% H_2_O, 0.1% formic acid; buffer B was 99.9% ACN, 0.1% formic acid. The HPLC gradient program delivered an acetonitrile gradient over 125 min. For the first 20 min, the flow rate was 400 μL/min with 2% B. The flow rate was then reduced to 200 μL/min and the fraction of solvent B increased in a linear fashion to 35% until 95.5 min. Solvent B was then increased to 80% over 5 min and maintained at that level until 107 min. The mobile phase was then reduced to 2% B until the end of the run (125 min). The parameters for DDA on the mass spectrometer were: 1 centroid MS (mass range 400–2000) followed by MS/MS on the 10 most abundant ions. General parameters were: activation type = CID, isolation width = 1 m/z, normalized collision energy = 35, activation Q = 0.25, activation time = 10 ms. The minimum threshold was 500, repeat count = 1, repeat duration = 30 s, exclusion size list = 500, exclusion duration = 30 s, exclusion mass width (by mass) = low 0.03, high 0.03.

#### Data-Dependent Acquisition MS Analysis

Mass spectrometry data were stored, searched, and analyzed using the ProHits laboratory information management system (LIMS) platform (Liu et al., 2016). Within ProHits, AB SCIEX WIFF files were first converted to an MGF format using WIFF2MGF converter and to an mzML format using ProteoWizard (v3.0.4468) and the AB SCIEX MS Data Converter (v.1.3 beta). Thermo Fisher scientific RAW mass spectrometry files were converted to mzML and mzXML using ProteoWizard (version 3.0.4468 - http://proteowizard.sourceforge.net/). The mzML and mzXML files were then searched using Mascot (version 2.3.02) and Comet (version 2012.02 rev.0). The spectra were searched with the RefSeq database (version 57, January 30th, 2013) acquired from NCBI against a total of 72,482 human and adenovirus sequences supplemented with common contaminants from the Max Planck Institute (http://141.61.102.106:8080/share.cgi?ssid=0f2gfuB) and the Global Proteome Machine (GPM; https://www.thegpm.org/crap/index.html). For TripleTOF 5600 files, the database parameters were set to search for tryptic cleavages, allowing up to two missed cleavage sites per peptide with a mass tolerance of 40 ppm for precursors with charges of +2 to +4 and a tolerance of ± 0.15 amu for fragment ions. For files analyzed on the Orbitrap Elite, charges of +2, +3 and +4 were allowed and the parent mass tolerance was set at 12 ppm while the fragment bin tolerance was set at 0.6 amu. Deamidated asparagine and glutamine and oxidized methionine were allowed as variable modifications. The results from each search engine were analyzed through the Trans-Proteomic Pipeline (version 4.6 OCCUPY rev 3) via the iProphet pipeline (Shteynberg et al., 2011). SAINTexpress version 3.3 (Teo et al., 2014) was used as a statistical tool to calculate the probability value of each potential protein-protein interaction compared to background contaminants using default parameters. Unless otherwise specified, controls were compressed by half, to a minimum of eight, to create “virtual controls” that provide for more stringent background estimation. Two unique peptide ions and a minimum iProphet probability of 0.95 were required for protein identification prior to SAINTexpress.

#### Data Independent Acquisition Analysis with MSPLIT

DIA MS data were analyzed using MSPLIT-DIA (version 1.0; Wang et al., 2015) implemented in ProHits 4.0 (Liu et al., 2016). To generate a sample-specific spectral library for the FLAG AP-MS dataset, peptide-spectrum matches (PSMs) from matched DDA runs (36 runs) were pooled by retaining only the spectrum with the lowest MS-GFDB (Beta version 1.0072 (6/30/2014), Kim et al., 2010) probability for each unique peptide sequence and precursor charge state, and a peptide-level false discovery rate (FDR) of 1% was enforced using a target-decoy strategy. The MS-GFDB parameters were set to search for tryptic cleavages, allowing no missed cleavage sites, 1 C_13_ atom per peptide with a mass tolerance of 50 ppm for precursors with charges of +2 to +4 and a tolerance of ± 50 ppm for fragment ions. Peptide length was limited to 8–30 amino acids. Variable modifications were deamidated asparagine and glutamine and oxidized methionine. The spectra were searched with the NCBI RefSeq database (version 57, January 30th, 2013) against a total of 36,241 human and adenovirus sequences supplemented with common contaminants from the Max Planck Institute (http://141.61.102.106:8080/share.cgi?ssid=0f2gfuB) and the Global Proteome Machine (GPM; https://www.thegpm.org/crap/index.html). This spectral library was further enhanced by incorporating non-redundant PSMs from the previously reported SWATH-Atlas library (Rosenberger et al., 2014) and decoys were appended using the decoy library command built in to MSPLIT, with a fragment mass tolerance of ± 0.05 Da. The spectral library was then used for protein identification by MSPLIT as previously described (Wang et al., 2015) with peptides identified by MSPLIT-DIA passing a 1% FDR subsequently matched to genes using ProHits 4.0 (Liu et al., 2016). The MSPLIT search parameters were as follows: parent mass tolerance of ± 25 Da and fragment mass tolerance of ± 50 ppm. When retention time was available within the spectral library, a cut-off of ± 5 min was applied to spectral matching as previously described (Wang et al., 2015). For the analysis of the endogenous IP-MS data, a separate library was built based on the 32 matched DDA runs only using essentially the same parameters as above except that only oxidized methionine was allowed as a variable modification.

#### MS Data Visualization and Archiving

Functional enrichment analysis was performed using g:Profiler (https://biit.cs.ut.ee/gprofiler/) using the default parameters. Dot plots and heatmaps were generated using ProHits-viz (https://prohits-viz.lunenfeld.ca; Knight et al., 2015), while Venn diagrams were generated in R (https://www.r-project.org/). Interaction networks were generated using Cytoscape (https://cytoscape.org/) with edge thickness based on spectral counts. Individual nodes were manually arranged in physical complexes. All MS files used in this study were deposited at MassIVE (https://massive.ucsd.edu/ProteoSAFe/static/massive.jsp) and the scored interactions associated with quantitative values are available for searching at prohits-web.lunenfeld.ca (project “BET rewiring”). Additional details (including MassIVE accession numbers and FTP download links) can be found in Table S2O.

#### Comparison of the Interaction Proteomics Datasets

To assess which fraction of the FLAG AP-MS dataset could be supported by endogenous IP-MS in either the parental HEK293 cells or in K562 cells, the datasets post-SAINT analysis (SAINT analysis was performed individually on each dataset) were directly compared at a fixed 1% FDR cutoff (excluding the BRDT dataset as BRDT is not expressed in HEK293 or K562 cells). We note that there were specific issues affecting each of the endogenous antibodies. The BRD2 antibody seemed to have a high number of cross-reacting proteins, notably to components of the mTOR amino acid sensing pathway (GATOR2 components MIOS, WDR24, WDR59, and SEH1L were all abundantly detected). The BRD3 antibody consistently yielded lower recovery of the bait and interactors, making quantitative comparisons to the other BETs more challenging. The BRD4 antibody was raised against a portion in the C terminus of the protein responsible to bind to P-TEFb: consistent with this, no P-TEFb was recovered using this antibody. Since it is not directly possible to compare the recovery of interactors with each BET in this set-up (or in our opinion to identify new interaction partners solely on the basis of this endogenous IP-MS analysis), we therefore used the data in aggregate to assess whether they could provide support to the FLAG AP-MS data. Looking at unique proteins that pass the 1% SAINT FDR cutoff when combining the t = 0 and t = 60 min time points for BRD2, BRD3 and BRD4, we recovered 425 proteins in the FLAG AP-MS dataset while 712 and 776 pass the cutoff in the endogenous HEK293 and K652 datasets, respectively. Looking at overlaps, the endogenous datasets provide high-confidence validation for 56.2% (i.e., 239 proteins) of the FLAG AP-MS interactors.

To compare the interactome observed in this study to the one reported by Dawson et al. (Dawson et al., 2011) in the absence of a statistically defined list of interactors, we first queried from their Supplemental Dataset 3 the “enrichment over reference,” and the “SSM used for quantification,” where is SSM is the number of spectrum-to-sequence matches for individual biological replicate that was reported by the authors. Then, the “enrichment over reference” was averaged across biological replicates while the SSM were summed to provide a dataset that more closely resembled to the analysis we have employed in the current manuscript. The compiled results of the proteins associated with BET proteins by both studies are now presented in Table S2B. Looking at the interactors that were enriched by a non-stringent arbitrary cutoff averaged enrichment over reference of ≥ 0.5 (and a minimal SSM of 2), we find evidence for 106 of the 319 interactors reported in our DMSO treated FLAG AP-MS dataset (for BRD2, BRD3 and BRD4; 33%).

#### Validation of Interactions by Immunoblotting

To validate selected protein-protein interactions, FLAG affinity purification was performed from two 150-mm plates of Flp-In T-REx HEK293 expressing a BirA^∗^-NLS-FLAG control or 3×FLAG-BRD2, BRD3, BRD4 or BRDT treated with 500 nM JQ1 for 0, 10, 60 or 240 min as described above. Following the capture and washing of FLAG-tagged complexes, proteins were eluted directly in 50 μL of Laemmli buffer by incubating the samples at 65°C for 10 min. Samples were then centrifuged at ∼1000 × *g* for 15 s and placed on a magnetic rack. The supernatants were transferred to fresh tubes while avoiding the transfer of any beads. Samples were further heated at 95°C for 5 min to fully denature proteins. For immunoblot analysis, 15 μL (1%) of input samples and 5 μL (10%) of AP samples were resolved by SDS-polyacrylamide gel electrophoresis (Bio-Rad Criterion Precast Gels, 4%–12% Bis-Tris, 1.0 mm, from Bio-Rad, CA), transferred to nitrocellulose, and blocked in TBS containing 5 mg/mL non-fat milk and 1% Tween 20 for 1 h at room temperature. Antibodies and the conditions in which they were used can be found in supplementary in the STAR Methods and in Table S1C. Detection on film was performed by chemiluminescence using the LumiGLO reagent (Cell Signaling Technology; #7003; 1:20).

#### Interactome and Kac Literature Overlap Analysis

Custom downloads of all interactions for bait proteins were created using the BioGRID version 3.4.157 released on January 25^th^, 2018 (https://thebiogrid.org/). Bait-prey and prey-bait relationships were both considered in overlap analysis; for BioGRID, only physical interactions were considered, and no other restriction were placed regarding experimental evidence. The complete acetylated lysine database was obtained from the PTMVar dataset (https://www.phosphosite.org/; June 2016 version).

#### Protein Expression and Purification

Plasmids were transformed into competent *E. coli* BL21(DE3)-R3-pRARE2 cells (a phage-resistant derivative of the BL21(DE3) strain), with a pRARE plasmid encoding rare codon tRNAs. Freshly grown colonies were cultured overnight in 2 × lysogeny broth (LB) supplemented with 50 μg/mL kanamycin and 34 μg/mL chloramphenicol at 37°C. One liter of pre-warmed terrific broth (TB) was inoculated with 10 mL of the overnight culture and incubated at 37°C. At an optical density at 600 nm (OD_600_) of 2.5, the culture was cooled to 18°C and expression was induced overnight at 18°C with 0.1 mM isopropyl-β-D-thiogalactopyranoside (IPTG). Cells were then harvested by centrifugation (8700 × *g*, 15 min, 4°C) in a Beckman Coulter Avanti J-20 XP centrifuge, and then re-suspended in lysis buffer (50 mM HEPES, pH 7.5 at 20°C, 500 mM NaCl, 5% glycerol, 1 mM tris(2-carboxyethyl)phosphine (TCEP) and 1:1000 (v/v) Protease Inhibitor Cocktail III (Calbiochem)). Cells were lysed three times at 4°C using a Basic Z Model Cell Disrupter (Constant Systems Ltd, UK) and DNA was removed by precipitation on ice for 30 min with 0.15% (v/v) of polyethyleneimine (PEI). Lysates were cleared by centrifugation (16,000 × *g* for 1 h at 4°C, JA 25.50 rotor, on a Beckman Coulter Avanti J-20 XP centrifuge). Supernatants were applied to nickel-nitrilotiacetic acid agarose columns (Ni-NTA, QIAGEN, 5 mL, equilibrated with 20 mL lysis buffer). The columns were washed once with 30 mL of lysis buffer, then with 20 mL of lysis buffer containing 30 mM Imidazole. Proteins were eluted using a step gradient of imidazole in lysis buffer (50, 100, 150, 2 × 250 mM imidazole in 50 mM HEPES, pH 7.5 at 25°C, 500 mM NaCl and 5% glycerol). All fractions were collected and monitored by SDS-polyacrylamide gel electrophoresis (Bio-Rad Criterion Precast Gels, 4%–12% Bis-Tris, 1.0 mm, from Bio-Rad, CA.). One half of the eluted proteins was treated overnight at 4°C with TEV protease to remove the hexa-histidine tag (for crystallography and other biophysical experiments). The other half of the proteins was kept with the hexa-histidine tag intact for use in SPOT assays. Both tagged and untagged proteins were further purified by size exclusion chromatography on a Superdex 75 16/60 HiLoad gel filtration column (GE Healthcare Life Sciences) on an ÄktaPrime plus system (GE/Amersham Biosciences). Recombinant BRD4 and BRD9 domains eluted as single symmetrical monomeric peaks. Samples were monitored by SDS-polyacrylamide gel electrophoresis and concentrated to 6–10 mg/mL in gel filtration buffer (10 mM HEPES pH 7.5, 500 mM NaCl and 5% glycerol) using Amicon® Ultra (EMD Millipore) concentrators with a 10 MWCO cut-off. Proteins were aliquoted into 100 μL fractions, flash frozen in liquid nitrogen and stored at −80°C until further use. Protein handling was performed on ice or in a cold room.

#### Electro-spray Quadrupole Time of Flight Mass Spectrometry

Purified protein samples were diluted down to 1 mg/mL with 0.1% formic acid and 60 μL was injected on an Agilent 6530 QTOF (Agilent Technologies - Palo Alto, CA) mass spectrometer with a Zorbax 5 μm 300SB-C3 column (Agilent Technologies - Palo Alto, CA) to ascertain the correct intact mass of the proteins (15.084 kDa for cleaved BRD4/BD1, 17.549 kDa for BRD4/BD1 with a hexa-His-tag; 15.036 kDa for the cleaved BRD4/BD2, 17.502 kDa for BRD4/BD2 with a hexa-His-tag; 12.926 kDa for BRD4/ET^mut^ with hexa-His-tag and 28.370 kDa for the cleaved BRD9 N terminus. Raw ion count data were deconvoluted using the Mass Hunter WorkStation software, Qualitative Analysis Vs B.06.00 (Agilent Technologies, Palo Alto, CA). Theoretical molecular masses of wild-type and mutant proteins were calculated using Expasy ProtParam (https://us.expasy.org/tools/protparam.html). The correct intact mass (within 1Da) and purity was confirmed for all recombinant proteins.

#### SPOT Peptide Assays

Cellulose-bound peptide arrays were prepared using standard Fmoc solid phase peptide synthesis on a MultiPep-RSi-Spotter (INTAVIS, Köln, Germany) according to the SPOT synthesis method provided by the manufacturer, as previously described (Picaud and Filippakopoulos, 2015). Peptides were synthesized on amino-functionalized cellulose membranes (Whatman Chromatography paper Grade 1CHR, GE Healthcare Life Sciences #3001-878) and the presence of SPOTed peptides was confirmed by ultraviolet light (UV, λ = 280 nM). The assay was performed using hexa-His-tagged BRD4 recombinant domains (BRD4/BD1, BRD4/BD2, BRD4/ET and BRD4/ET^mut^). Proteins bound to peptides were detected using HPR-conjugated anti-His antibody (Novagene, # 71841) and the Pierce ECL Western Blotting Substrate (Thermo Fisher Scientific, # 32106). Chemiluminescence was detected with an image reader (Fujifilm LAS-4000 ver.2.0), typically using an incremental exposure time of 5 min for a total of 80 min (or until saturation was reached, in the case of very strong signal). The dilution of HPR conjugated anti-His antibody was adapted as a function of the strength of the signal observed (from 1:5000 for weak binders, to 1:50000 for strong binders) to limit the rapid decay of the emission signal during the chemiluminescence detection. Peptide locations on the arrays and their sequences are provided in Table S3.

#### Discovery of Histone-like Kac-X_2_-Kac Motifs Recognized by BET BRDs

We previously established that histone H4 peptides carrying two Kac linked by two residues, preferably with a glycine at the first position (for example, SGRG-**Kac**-**G**G-**Kac**-GLG in the H4 K5ac/K8ac sequence), exhibit strong binding toward BET BRDs, with both Kac binding within the BRD cavity (Filippakopoulos et al., 2012). Furthermore, we hypothesized that flanking sequences contribute to binding specificity on the basis of the differentially electrostatically charged rim regions surrounding the central Kac site (Figure S2A). To further identify potential Kac-XX-Kac binding sites beyond histones, we interrogated all curated protein sequences found in the Uniprot/SwissProt database (April 2017 version) using custom-made scripts in R (https://www.r-project.org/). We found 43,755 unique sequences in 13,660 proteins, encoding the 400 possible combinations of K-XX-K (where each X can be one of the 20 naturally occurring amino-acids). However, only 2,112 of these peptides contain linkers that are found in the four core histones (we defined “histone-like” linkers as the 13 following XX combinations: GG, GS, DG, AA, AP, AV, AQ, AR, SA, VL, LN, TA, TP). We synthesized all peptides from this set (2530, including redundant peptides, e.g., those found in splice variants) onto cellulose SPOT arrays and further added a small set of non-histone-like linked K-XX-K peptides employing a DS linker (213 peptides) as well as a small set of 91 K-GX-K peptides (from a total of 2521 possible unique K-GX-K sequences, cherry-picking sequences found in nuclear proteins, transcription factors, and other signaling proteins). Short peptides (15-mers) containing potential Kac sites within the above sequences were synthesized on cellulose SPOT arrays and screened against the BRD4 BRDs (BD1 and BD2). The results are summarized in Tables S3C and S3D.

#### Discovery of Novel Kac Sequences Recognized by BET BRDs

Discovery of motifs containing Kac sequences was performed AP-MS data for each BET protein, with and without 60 min treatment with the pan-BET BRD inhibitor JQ1, which blocks BRD-initiated Kac dependent interactions. Proteins that were present in the original AP-MS set and were lost (log_2_ fold change ≤ −2) upon 500 nM JQ1 treatment for 60 min were considered as potential Kac-dependent interactors. Annotated Kac sites reported in the PhosphoSitePlus dataset (https://www.phosphosite.org/; June 2016 version) were identified using custom made scripts in R (https://www.r-project.org/). 15-aa sequences around central Kac epitopes arising from this analysis were further analyzed: first the percentage of each amino-acid was calculated for each position, relative to the central Kac, then the relative enrichment (i.e., “positional enrichment”) of each amino acid was calculated at each position, taking into account the total amount of each amino-acid in each set.

From the 124 proteins that were lost from the 3xFLAG-BRD4 AP-MS map following JQ1 treatment (ie those proteins that had no spectral counts following 60 min JQ1 treatment, Table S2E) 89 contained 456 annotated Kac-sites in PhosphoSitePlus (https://www.phosphosite.org/). We identified across these sequences enrichment of His at −3, Phe at −2, Gly at −1, Tyr at +1, Ile or Arg at +2 and Lys at +3. Motifs carrying two acetylated lysines separated by two amino acid linkers (Kac-XX-Kac, X: any amino-acid) were previously found to be recognized by BET BRDs (Filippakopoulos et al., 2012, Morinière et al., 2009), in agreement with the above analysis, though the enrichment was modest. The strong enrichment of Kac-Y motifs in our analysis was further characterized structurally and in solution.

The same analysis yielded 83 proteins (71 of which contain 425 annotated Kac-epitopes) for BRD2, 101 proteins (65 of which contain 342 annotated Kac-epitopes) for BRD3 and 39 proteins (29 of which contain 114 annotated Kac-epitopes) for BRDT. The identified Kac-containing sequences can be found in Tables S3F–S3I.

#### Extra-Terminal Domain Consensus Motif Discovery

Discovery of potential ET-specific motifs was performed on the AP-MS data of full-length 3×FLAG tagged BETs, following the observation that several interacting partners exhibited little to no change in spectral counts (within a log_2_ fold change of ± 2, Table S2E) following treatment with JQ1 (BRD2: 48; BRD3: 146; BRD4: 67; BRDT: 30). Focusing on BRD4, 12 of its interactors were recapitulated in a pull-down employing recombinant BRD4/ET domain as bait, including BRD9 and WHSC1. Identification of linear motifs within BRD9 and WHSC1 was performed as shown in Figure 5 and Figure S4 respectively (see also Tables S3K–S3AB). Interrogation of the full-length sequences of the remaining proteins for motifs similar to those identified and verified for BRD9 (LKLVLKV) and WHSC1 (IKLKI) was performed in R (https://www.r-project.org/) using custom made scripts. First, a minimal combination of both BRD9 and WHSC1 motifs was constructed ([+]Φ(Φ/[+], where Φ is a hydrophobic residue, including Leu, Ile, Met and Val and [+] a positively charged residue including Arg and Lys) to extract the maximum number of potential interaction. This uncovered 135 potential sites within the 12 proteins common between FL-BRD4 and BRD4/ET, which were profiled on a cellulose SPOT array with recombinant hexa-His-tagged BRD4/ET (Tables S3P–S3Q). Medium-strong SPOTs (i.e., with an intensity above 65% compared to multiple hexa-His-controls) were considered potential “hits” and were further profiled with SPOT arrays and single amino acid alanine scanning (i.e., in a given 18-mer, each position, from aa 2 to aa 17, was sequentially mutated to an alanine), and the resulting 17 peptides (1 wild-type and 16 mutated sequences) were profiled against the recombinant hexa-his-tagged BRD4/ET domain (Table S3R). Quantification of alanine-scanned membranes allowed us to define the contribution of each position to binding, with 100% suggesting no change in binding compared to the wild-type sequence and 0% representing a total loss in binding. Interrogation of the remaining 55 proteins that had largely invariant spectral counts following 60 min treatment with JQ1 for similar motifs, identified 344 potential motifs in 51 proteins. SPOT evaluation of these motifs (Table S3S) followed by alanine-scanning of strong “hits” (Tables S3T and S3U) resulted in a number of similar motifs bound to BRD4/ET. Notably, one of the 12 proteins common between FL-BRD4 and BRD4/ET AP-MS datasets (RPS26) did not yield any binding motif(s). However, a sequence scan of full-length RPS26 by SPOT array (Figure S4; Table S3V) identified a peptide region, Residues 31-48) which was recognized by the wild-type but not the mutant BRD4/ET domain, and alanine scanning of this region highlighted contribution to binding from a BRD9-like motif that included a phenylalanine residue (KFVIK motif; Figure S4; Table S3W). Interrogation of the 67 relatively unchanged proteins (following 60 min of JQ1 treatment) for a [+]Φ(Φ/[+]) motif (where Φ now included Phe in addition to Leu, Val, Ile and Met) identified an additional 93 potential binding sites. SPOT evaluation of these sites followed by alanine scanning of very strong hits (i.e., with intensity > 85% compared to control peptides) resulted in similar motifs to those identified for BRD9 and WHSC1 (Tables S3X–S3Z). Manual sequence alignment using all alanine quantifications, allowed the assembly of sequences that were used to construct the five resulting motifs (BRD9-like: *fn*-(Φ/[+])ΦΦ(Φ/[+])-*fc*; WHSC1/L1-like: *fn*-Φ[+]Φ[+]-*fc*; CM1: *fn*-[+]x[+]-*fc*; CM2: *fn*-[+](H)-*fc*; and CM3: *fn*-[+]-*fc*) summarized in Table S3AA. LOGOs presented in Figures 5 and S5 were generated using WebLogo (http://weblogo.berkeley.edu). Identified and verified sequences are summarized in Table S3AB. Please note that at the time of submission of this work, some of the peptide sequences investigated were found to be “outdated” when comparing against the 2018 version of UniProt – these have been annotated in Tables S3P, S3S, S3X, S3AA, and S3AB.

#### Custom Peptide Synthesis

Peptides (wild-type or modified) used in biophysical or crystallographic experiments were synthesized by the TUFTS Core Facility, on a 0.1 mmol scale with one round of HPLC purification. Peptides were re-suspended into water or buffer (50 mM HEPES pH 7.5, 150 mM NaCl) based on their overall charge. All peptide solutions were then further purified using PD MiniTrap G-10 columns (GE Healthcare Life sciences) according to the manufacturer’s instructions, to remove any remaining chemical residuals from the synthesis. Peptide details are summarized in Table S1E.

#### Isothermal Titration Calorimetry

Experiments were performed on an ITC200 titration micro-calorimeter (MicroCal, LLC, GE Healthcare) equipped with a washing module, with a cell volume of 0.2003 mL and a 40 μL micro-syringe. Experiments were performed at 15°C with stirring at 1000 rpm, in ITC buffer (50 mM HEPES pH 7.5 at 25°C, 150 mM NaCl). The micro-syringe was loaded with a solution of peptide sample (1226 - 1433 μM, in ITC buffer) and was carefully inserted into the calorimetric cell which was filled with protein (0.2 mL, 36 - 59 μM) in ITC buffer. Following baseline equilibration an additional delay of 60 s was applied. All titrations were conducted using an initial control injection of 0.3 μL followed by 38 identical injections of 1 μL with a duration of 2 s (per injection) and 120 s intervals between injections. The titration experiments were designed as to ensure complete saturation of the proteins before the final injection. The heat of dilution for the peptides were independent of their concentration and corresponded to the heat observed from the last injection, following saturation of ligand binding, thus facilitating the estimation of the baseline of each titration from the last injection. The collected data were corrected for peptide heats of dilution (measured in separate experiments by titrating the peptides into ITC buffer) and deconvoluted using the MicroCal Origin software to yield enthalpies of binding (**Δ***H*) and binding constants (*K*_B_) as previously described (Filippakopoulos et al., 2012). Thermodynamic parameters were calculated using the basic equation of thermodynamics (**Δ***G* = **Δ***H* - T**Δ***S* = -RTln*K*_B_, where **Δ***G*, **Δ***H* and **Δ***S* are the changes in the free energy, enthalpy, and entropy of binding, respectively). In all cases a single binding site model, supplied with the MicroCal Origin software package was employed. Heat capacities (ΔCp) for H4 (K5ac/K8ac) and SRPK1 (K585acY) peptides were calculated in Origin Pro (v.9.4 OriginLab Corporation) from the slope of linear least-square fitted ΔH/T plots. Thermodynamic parameters are listed in Tables S4A and S4B and peptide sequences are listed in Table S1E.

#### Sedimentation Velocity Analytical Ultracentrifugation

SV experiments were performed on a Beckman Optima XL-I Analytical Ultracentrifuge (Beckman Instruments, Palo Alto, CA) equipped with an AnTi-50 rotor and cells with double sector centerpieces. Protein samples were studied at a concentration of 50-60 μM in 50 mM HEPES pH 7.5 at 25°C, 100 mM NaCl at 4°C, employing a rotor speed of 40,000 rpm. Radial absorbance scans were collected using absorbance optics at 280 nm in continuous scan mode, in 2 min intervals with a redial step size of 0.003 cm. Aliquots (300 μL) were loaded into the sample channels of double channel 12 mm centerpieces, and 310 μL of buffer was loaded into the reference channels. Data were analyzed using the SEDFIT (v.15.01b, Schuck, 2000) software package whereby differential sedimentation coefficient distributions (c(s) distributions) were obtained by direct boundary modeling to Lamm Equation solutions. Sedimentation coefficients (s) were obtained by integration of individual peaks in the calculated c(s) distributions, after fitting of the frictional ratio (f/f_o_), allowing these distributions to be corrected for the effects of diffusion. The software package SEDNTERP (version 1.08) was used to convert the obtained sedimentation coefficient values to equivalent values in water at 20°C, taking into account the solvent density (1.00802 g/mL), viscosity (1.567 × 10^−2^ poise), and partial specific volume (calculated in Table S4C) of each protein construct tested. Translational frictional ratios were calculated from the s_20,w_ values using the following equation:

$$\left(f_{20,w}^{0}/f_{0}\right)=\;\left(M\left(1-\bar{v}\rho_{0}\right)/N_{A}s_{20,w}^{0}\right)/\left(6\pi \eta_{0}\sqrt[{3}]{\left(3M\bar{v}/4\pi N_{A}\right)}\right)$$

where *M* is the molecular weight, $\bar{v}$ is the partial specific volume, *N*_*A*_ is Avogadro’s number and s^o^_w,20_ is the sedimentation coefficient corrected to the standard conditions of density, ρ_0_, and viscosity, η_0_, of water at 20°C, and extrapolated to infinite dilution.

#### ALPHAScreen Assay

The assay was performed as previously described (Filippakopoulos et al., 2010) with minor modifications from the manufacturer’s protocol (PerkinElmer, USA). All reagents were diluted in 50 mM HEPES, 250 mM NaCl, 0.1% BSA, pH 7.4 supplemented with 0.05% CHAPS and allowed to equilibrate to room temperature. A 11-point 1:2 serial dilution of the ligands was prepared over the range of 5 μM – 4.88 nM and 4 μL transferred to a low-volume 384-well plate (ProxiPlateTM-384 Plus, PerkinElmer, USA). BRD4/BD1 protein and the biotinylated SRPK1 peptide: RKLIVAG-Kac-YSKEFFTKKGDLK(Biotin)-OH (TUFTS, USA) were mixed and pre-incubated for 30 min at room temperature, before addition of 8 μL of the protein/peptide mix to the plate. The amount of protein and peptide used was calculated in order to have a final concentration BRD4/BD1 and SRPK1 peptide of 1.6 μM and 0.8 μM respectively in the 20 μL reaction volume. The plate was sealed and incubated at room temperature for another 30 min before the addition of 4 μL of streptavidin-coated donor beads (25 μg/mL) and 4 μL nickel chelate acceptor beads (25 μg/mL) under low light conditions. The plate was foil-sealed to protect the reaction mixture from light, incubated at room temperature for 60 min and read on a PHERAstar FS plate reader (BMG Labtech, Germany) using an AlphaScreen 680 excitation/570 emission filter set. IC_50_ values were calculated in Origin Pro (v.9.4 OriginLab Corporation) after normalization against corresponding DMSO controls and are given as the final concentration of compound in the 20 μL reaction volume.

#### Crystallization

The purified BRD4/BD1 protein buffer was exchanged with 10 mM HEPES pH7.5, 150 mM NaCl and 2% (w/v) glycerol, on an Äkta pure system using a Sephadex 10/300 GL column (GE/Amersham Biosciences). BRD4/BD1 protein (5 - 10 mg/mL) was then incubated on ice for 30 min with 2 - 5 mM final peptide. Peptide/protein mixtures were set up for crystallization using a mosquito crystallization robot (TTP Labtech, Royston UK). Coarse screens were typically setup onto Greiner 3-well plates using three different drop ratios of precipitant to protein per condition (100+50 nL, 75+75 nL and 50+100 nL). Initial hits were optimized using Greiner 1-well plates and scaling up the drop sizes in steps. All crystallizations were performed using the sitting drop vapor diffusion method at 4°C. Crystals of BRD4/BD1 with an E2F1 peptide (H114-PG-**Kac**-GV-**Kac**-SPGEKSRY-E129 – Kac-XX-Kac motif) were grown by mixing 50 nL of the protein (12 mg/mL in 10 mM HEPES pH 7.5, 500 mM NaCl, 5% glycerol) with 100 nL of reservoir solution containing 20.0% PEG3350, 10.0% ethylene glycol and 0.2 M NaCHO. Crystals of BRD4/BD1 with a GATA1 peptide (A309-SG-**Kac**-GK-**Kac**-KR-G318-***Y*** – Kac-XX-Kac motif) were grown by mixing 50 nL of the protein (12 mg/mL in 10 mM HEPES pH 7.5, 500 mM NaCl, 5% glycerol) with 100 nL of reservoir solution containing 30.0% PEG1000 and 0.1 M SPG pH 8.0. Crystals of BRD4/BD1 with an ATRX peptide (H1027-FP-**Kac**-GI-**Kac**-QI-K1036-***Y*** – Kac-XX-Kac motif) were grown by mixing 100 nL of the protein (8 mg/mL in 10 mM HEPES pH 7.5, 500 mM NaCl, 5% glycerol) with 50 nL of reservoir solution containing 20.0% PEG6000, 10.0% ethylene glycol, 0.1 M HEPES pH 7.0 and 0.2 M LiCl. Crystals of BRD4/BD1 with a POLR2A peptide (S772-GA-**Kac**-GS-**Kac**-IN-I781-***Y*** – Kac-XX-Kac motif) were grown by mixing 75 nL of the protein (8 mg/mL in 10 mM HEPES pH 7.5, 500 mM NaCl, 5% glycerol) with an equal volume of reservoir solution containing 20.0% PEG6000, 10.0% ethylene glycol, 0.1 M HEPES pH 7.0 and 0.1 M MgCl_2_. Crystals of BRD4/BD1 with a TOP2A peptide (G1198-KA-**Kac**-GK-**Kac**-TQ-M1207-***Y*** – Kac-XX-Kac motif) were grown by mixing 75 nL of the protein (8 mg/mL in 10 mM HEPES pH 7.5, 500 mM NaCl, 5% glycerol) with an equal volume of reservoir solution containing 20.0% PEG3350, 10.0% ethylene glycol and 0.2 M NaI. Crystals of BRD4/BD1 with an H3 K9ac/K14ac peptide (K4-QTAR-**Kac**-STGG-**Kac**-APRK-Q20-***Y*** – Kac-XXXX-Kac motif) were grown by mixing 50 nL of the protein (10 mg/mL in 10 mM HEPES pH 7.5, 500 mM NaCl, 5% glycerol) with 100 nL of reservoir solution containing 0.20 M LiCl, 0.1 M Tris-HCl pH 8.0, 20.0% PEG 6K and 10.0% ethylene glycol. Crystals of BRD4/BD1 with an H3 K9ac/pS10/K14ac peptide (K4-QTAR-**Kac**-**pS**-TGG-**Kac**-APRK-Q20-***Y*** – Kac-XXXX-Kac motif) were grown by mixing 75 nL of the protein (9 mg/mL in 10 mM HEPES pH 7.5, 500 mM NaCl, 5% glycerol) with 75 nL of reservoir solution containing 0.20 M Na(CH_3_COO), 0.1 M BTProp pH 7.5, 20.0% PEG 3350 and 10.0% ethylene glycol. Crystals of BRD4/BD1 with a BAZ1B peptide (F217-LPH-**Kac**-YDVK-L226 – KacY motif) were grown by mixing 50 nL of the protein (9 mg/mL in 10 mM HEPES pH 7.5, 500 mM NaCl, 5% glycerol) with 100 nL of reservoir solution containing 0.1 M bis-tris-propane pH 8.5, 0.02 M sodium/potassium phosphate, 20.0% PEG3350 and 10.0% ethylene glycol. Crystals of BRD4/BD1 with an SRPK1 peptide (V582-AG-**Kac**-YS-**Kac**-EF-F591-***Y*** – KacY motif) were grown by mixing 50 nL of the protein (5.3 mg/mL in 10 mM HEPES pH 7.5, 500 mM NaCl, 5% glycerol) with 100 nL of reservoir solution containing 0.1 M PCB pH 7.0 and 30.0% PEG 3350. In all cases crystals appeared within several days from sitting drop plates at 4°C.

#### Data Collection and Structure Determination

Prior to data collection, all crystals were transferred to a solution consisting of the precipitation buffer supplemented with ethylene glycol and subsequently flash frozen in liquid nitrogen. Data were collected at Diamond Lightsource on beamline I24 at a wavelength of 0.9686 Å (BRD4/BD1 complexes with H3 K9acK14ac, H3 K9ac/pS10/K14ac and POLR2A K775ac/K778ac), beamline I02 at a wavelength of 0.97949 Å (BRD4/BD1 complexes with SRPK1 K585ac/K588ac, ATRX K1030ac/K1033ac and GATA1 K312ac/K315ac) or beamline I03 at a wavelength of 0.97625 Å (BRD4/BD1 complexes with BAZ1B K221ac, TOP2A K1201ac/K1204ac, E2F1 K117ac/K120ac and SIRT7 K272ac/K275ac). Data processing was carried out using the CCP4 suite (v.6.5 & v.7.0 Winn et al., 2011). Data were integrated with XDS (Kabsch, 2010) and scaled with SCALA (v.3.3.2) or AIMLESS (v.0.7.3, CCP4 v.7). Initial phases were calculated by molecular replacement with PHASER (v.2.5, CCP4 v.7) using the known model of BRD4/BD1 (PDB: 2OSS). Automated model building with ARP/wARP (Perrakis et al., 1999) resulted in >90% complete models. Refinement was performed with REFMAC after several rounds of manual rebuilding with COOT. The quality of the final models was validated with the MOLPROBITY server (http://molprobity.biochem.duke.edu/). Hydrogen atoms were included in late refinement cycles. Data collection and refinement statistics can be found in Table S5.

#### Small Angle X-Ray Scattering

SAXS data were collected at Diamond Light Source beamline B21 using an in-line HPLC connected to a Shodex KW404-4F column in a buffer containing 20 mM HEPES pH 7.5, 150 mM NaCl, 2% glycerol and 0.5 mM TCEP and a flow rate of 0.16 mL/min. Scattering data were collected in continuous mode and initial data reduction was performed in the DAWN software suite (Basham et al., 2015), with background subtraction and averaging of 1D profiles performed using the ScÅtter suite (www.biosis.net). The ATSAS suite (v.2.8, Franke et al., 2017) was used to first calculate distance (pair) distribution functions (p(r)) with GNOM, which were subsequently used as input to DAMMIN for *ab initio* shape determination. The results from 23 separate DAMMIN runs in fast mode were aligned using SUPCOMB and an averaged model was created in DAMAVER, which was used as input to a final round of shape determination using DAMMIN in slow mode. The initial DAMMIN models showed low divergence with mean values of normalized spatial discrepancy (NSD) of 0.714 (BRD2), 0.613 (BRD3), 0.619 (BRD4) and 0.619 (BRDT), and the final models show good agreement with the data with χ^2^ values of 1.16 (BRD2), 1.28 (BRD3), 1.05 (BRD4) and 0.92 (BRDT). Docking of the first and second bromodomains into the bead models was performed manually in PyMOL using PDB: 2OUO (BRD4/BD2) with the constraints of the C terminus of the first bromodomain and the N terminus of the second bromodomain pointing toward the center of the molecule.

#### Confocal Microscopy for Co-localization Using the LacO Array

U-2 OS-LacO cells were transfected with the indicated combinations of plasmids using FuGENE6 according to the manufacturer’s protocol and incubated for 24 h. Transfected cells were plated into an 8-well imaging chamber (Miltenyi Biotec, cat.# 130-098-273). After 24 h, cells were fixed with 4% paraformaldehyde (PFA) in PBS for 10 min at room temperature. Cells were then washed three times with PBS and mounted with mounting medium (90% glycerol and 10% 20 mM TRIS-HCl pH 8.0). All images were obtained using a Zeiss LSM 710 scan-head (Zeiss GmbH, Jena, Germany) coupled to an inverted Zeiss Axio Observer Z1 microscope equipped with a high-numerical-aperture (N. A. 1.40) 63 × oil immersion objective (Zeiss GmbH, Jena, Germany). A 488 nm excitation laser and a 494-555 nm emission filter were used to detect GFP fluorescence. A 594 nm excitation laser and a 598-700 nm emission filter were used to detect mCherry fluorescence.

#### LacO Array High Content Analysis

U-2 OS-LacO cells were transfected with the indicated combinations of plasmids using FuGENE6 according to the manufacturer’s protocol and incubated for 24 h. Transfected cells were plated onto a CellCarrier-96 Black plate (PerkinElmer, cat.# 6005558). After 24 h, cells were treated with 1 μg/mL Hoechst 33342 solution (Thermo Fisher, cat.# H3570) for 20 min and were fixed with 4% PFA in PBS for 10 min at room temperature. Cells were washed three times with PBS and mounted with mounting medium. All images were obtained with an Operetta high-content imaging system (PerkinElmer) using a 40 × objective lens. Hoechst, GFP, and mCherry signals were acquired with a standard filter set originally equipped with the Operetta instrument. At least 100 field images per well were acquired. Data were analyzed with the “find spots” algorithm of Harmony analysis software (version 4.1, PerkinElmer). Numbers of mCherry dots, GFP dots and co-localized dots in the nuclear (Hoechst positive) region were automatically counted without bias. Error bars represent the standard deviation from three independent experiments.

#### Live-Cell Imaging of BET Responses to JQ1 Treatment

U-2 OS cells were seeded onto 8-well LabTek II imaging chambers (ThermoFisher) in complete media with 1 μg/mL tetracycline and grown for 24 h to allow expression of GFP-tagged protein. Before imaging, the media was replaced with 200 μL of phenol red-free DMEM supplemented with 10% FBS, 1 mM sodium pyruvate, and 1 × Glutamax (all Life Technologies). Images stacks were acquired on an imaging system (DeltaVision Elite, GE Healthcare). Cells were imaged at 37°C in 5% CO2 at 60 ×, 1.42NA, with 2 × 2 binning. Image Z stacks of 24 μm were aquired at 2 μm intervals over 2 - 5 min as indicated. 20 s after the start of data acquisition, 100 μL of warm media containing 1.5 μM JQ1 was added to each cell chamber manually for a final concentration of 500 nM. The exposure time was 10 ms at 32% for GFP-tagged bait protein. Z stacks were collected, deconvolved using softWoRx (v5.0, Applied Precision) and displayed as maximum intensity projections (pixel size 0.1075 μm). Images were cropped in ImageJ (National Institutes of Health). For all quantitatively compared images, identical imaging conditions (including exposure times) were used, and maximum intensity projections of Z stacks were analyzed.

#### Immunofluorescence Microscopy of Nucleolar Proteins

HeLa cells were seeded on coverslips at low density in Opti-MEM medium and then transfected with the appropriate siRNA using Lipofectamine RNAiMAX as per the manufacturer protocol, see STAR Methods and Table S1C for details. 48 hr later, cells were fixed with 3.7% paraformaldehyde/PBS and permeabilized in 0.3% Triton X-100 in PBS. Mouse anti-BRD3 antibody (1:100; ab50818, Abcam) and anti-TCOF1 (1:500; HPA038237; Sigma-Aldrich) were used to identify BRD3 and TCOF1, respectively. Proteins were visualized with goat anti-mouse or anti-rabbit coupled to Alexa Fluor 488 or 555 antibodies (1:1,000; A11001, A11008, A21422, A21428; Invitrogen). DNA was detected with DAPI staining. Immunofluorescence was observed by confocal microscopy on a Nikon Eclipse C1si instrument.

#### Quantitation of Ribosomal RNA by Immunofluorescence

U-2 OS cells were seeded into 12-well plate containing coverslips in complete media with varied level of tetracycline (0, 100 or 1000 ng/mL) and grown for 48 h to allow expression of GFP tagged protein. Cells were then treated or not with 500 nM JQ1 and then subsequently fed 5-ethyl uridine (5-EU) for 1 h. Cells were then fixed with 4% paraformaldehyde (PFA) in PBS for 15 min at room temperature and then stained for BRD3 and fibrillarin (see Table S1C for antibody details). Nascent RNA was labeled using the Click-iT RNA Alexa Fluor 594 imaging kit (Catalogue # C10330; Molecular probes, Thermo Fisher Scientific) as per the supplier’s instructions. Cells were imaged as above and ribosomal RNA (rRNA) was defined by generating a mask of the 5-EU signal overlap with fibrillarin. For all quantitatively compared images, identical imaging conditions (including exposure times) were used, and maximum intensity projections of Z stacks were analyzed using custom MATLAB scripts. Cell population intensities were compared across different treatments using the two-tailed Student’s t test.

#### ChIP-qPCR

ChIP was performed as described above from 2 × 15 cm plates of U-2 OS cells at ∼75% confluence following induction with different concentrations of tetracycline and treatment with 500 nM JQ1 for 1 h as indicated. For each sample, 2% of the resulting eluate (1/50 μL) was used for each PCR reaction. qRT-PCR was performed using a 7500 Real-time PCR machine (Applied Biosystems; Thermo Fisher Scientific) using *Power* SYBR Green PCR Master Mix, as per the manufacturer’s instructions. Primers used for qRT-PCR are listed in Table S1C.

#### Chromatin Immunoprecipitation

Flp-In T-REx U-2 OS cells stably expressing GFP tagged BRD3 WT or a (BD1:2)^mut^ construct were grown in two 15 cm plates to ∼75% confluence and induced with 1 μg/mL tetracycline for 48 h. Cells were then washed with PBS and crosslinked with 1% formaldehyde in PBS for 15 min at room temperature before quenching the reaction with 125 mM glycine in PBS for 5 min at room temperature. Cells were then pelleted and frozen at −80°C until ChIP was performed as described previously (Schmidt et al., 2009) using 7 μg of anti-GFP (ab290, Abcam) antibody. Next, ChIP DNA was prepared for Illumina sequencing by blunt-end repair, dA-tailing, and ligating Illumina adaptors using the NEBNext DNA library preparation kit (NEB, #E6040L). The libraries were PCR amplified by 16 cycles using multiplexing index primers (NEB, #E7335L), size selected (200-350 bp, PippinPrep 2% gel, Sage Science) and quantified with 2100 Bioanalyzer (Agilent). Input control DNA (220 ng) extracted from sonicated cell lysates of each sample were processed in parallel. For each library 50 bp reads were sequenced with the HiSeq2500 (Illumina).

#### ChIP-Seq Data Analysis

For GFP-BRD3 ChIP-seq experiments, 50bp single-end reads were obtained. Fastq files of other U-2 OS ChIP-seq datasets were downloaded through ArrayExpress (E-GEOD-44672, Walz et al., 2014). Fastq files of TCOF1 ChIP-seq and the corresponding input in HeLa cells were obtained from GSE89420 (Calo et al., 2018). Raw reads were first trimmed using Trimmomatic (v.0.36) with default parameters. As reads from E-GEOD-44672 are only 33bp in length they were not trimmed. Reads were then aligned to the human genome (hg19) using bwa (v.0.7.8, Li and Durbin, 2009) with default parameters. Uniquely mappable reads and reads that do not map to the ENCODE hg19 blacklist regions (see Key Resources Table) were used for all downstream analyses. ChIP-seq browser tracks were generated using BigWig files that were visualized on the UCSC genome browser.

To analyze protein binding on rDNA regions, reads were mapped to a customized genome where the human rDNA sequences were added as a separate chromosome on to the human genome fasta file. Reads that mapped to rDNA were extended to 150bps and their coverage across rDNA regions were generated with the bedtools genomecov function. R package “Sushi” (v.1.16, Phanstiel et al., 2014) was used to visualize ChIP-seq signals on the rDNA region. The rDNA structure is annotated based off GenBank. Read counts at each position were normalized to total library counts (RPM: reads per million mapped reads) and normalized input read counts were subtracted. A correlation heatmap was generated based on the Spearman correlations between each pairs of samples on the rDNA region with 1bp resolution. Positions were no reads bind to in all samples were excluded.

SRA files of published datasets (BRD4 in mESCs: GSE69140; CHD4 in mESCs: GSE61188; BRD4 in mouse leukemic cells: GSE52279; BRD9 in mouse leukemic cells: GSE79360) were obtained from NCBI’s Gene Expression Omnibus using the SRA toolkit (v.2.9.0) and were mapped to the mouse reference genome (mm9) with bowtie2 (v.2.2.3.4.1) using default parameters.

#### ChIP-Seq Peak Calling

Filtered aligned reads from biological replicates were pooled and used for peak calling. Peak calling was performed using MACS2 (version 2.1.0 20151222) with the parameters “–broad–broad-cutoff 0.01–fe-cutoff 2.”

#### ChIP-Seq Promoter Enrichment

The average ChIP-seq signal of BRD3 WT and BRD3 (BD1:2)^mut^ across all refGene gene bodies were generated using ngs.plot (v.2.6.3). For the visualization of published datasets for BRD4, CHD4 and BRD9, genome ChIP-seq profiles were generated using *bamCoverage* from deepTools v.2.0 and heatmaps were generated with *computeMatrix*/*plotHeatmap* from deepTools v.2.0 or ngs.plot.

#### ChIP-Seq Signal Density Heatmap

Read density of ChIP-seq experiments around peak centers were calculated using seqMINER (v.1.3.3) with reads extended to 150bp. Read counts were averaged for every 50bp for 5kb up/downstream of peak centers. This count matrix was then exported, processed, and visualized in R (https://www.r-project.org/). For each library, read counts were scaled to 10 million. Next, corresponding input reads were subtracted. Peaks were separated into “WT only,” “WT and (BD1:2)^mut^ shared” and “(BD1:2)^mut^ only” as previously described, and were ranked based on the sum of WT read counts. To compare ChIP signals of different factors, Spearman correlation coefficients were calculate based on the sum of normalized read counts around the center ± 5kb) of each WT only peak.

#### Chromatin State Segmentations

Chromatin states were generated using ChromHMM (Ernst and Kellis, 2012) with 10 states included in the model. Merged BRD3 WT and (BD1:2)^mut^ ChIP-seq reads, as well as ChIP-seq reads of H3K27ac, H3K4me3, and H3K4me1 (obtained from GSE44672) were used as inputs.

#### BET Protein Essentiality Analysis in Cancer Cells

Previously reported CERES scores for BRD3, BRD4, PTEN and POLR2A in 342 cancer cell lines (Meyers et al., 2017) were extracted from the Supplemental data and represented as Violin Plots within R (https://www.r-project.org/).

### Quantification and Statistical Analysis

#### Proteomics Quantification and Statistical Analysis

Details of the peptide/protein identification software and interaction proteomics scoring with the statistical tool SAINTexpress version 3.3 (Teo et al., 2014) are provided above.

#### Quantification and Statistical Analysis of Microscopy Data

Quantification of the different types of fluorescence microscopy images was performed with vendor-specific software and/or custom MATLAB scripts and is described in the respective sections. Standard statistical tools (e.g., two-tailed Student’s t test) were used to evaluate the data.

#### ChIP-qPCR Quantitation

ChIP-qPCR fold enrichment was first computed by performing a background (non-specific rabbit antibody) deletion and subsequently computing the fold increase in signal relative to the background signal. Triplicate measurements were taken for each data point and error bars represent the standard error of the mean.

#### ChIP-Seq Peak Overlap

Overlapping of peaks was performed using the bedtools “*intersectBed*” function. A consensus peak set was first generated by merging overlapping peaks with the “merge” function implemented in bedtools. This consensus peak set was then overlapped with peaks called in both the BRD3 WT and BRD3 (BD1:2)^mut^ samples and classified into “WT only,” “WT and (BD1:2)^mut^ shared” and “(BD1:2)^mut^ only.” Significance of overlap (p < 10^−6^) was tested with GAT (Heger et al., 2013) with 10^6^ permutations, using BRD4 peaks (segments) onto CHD4 or BRD9 peaks (annotation) within open chromatin regions determined as DnaseI sites (workspace) obtained from the Encode project (see Key Resources Table).

### Data and Software Availability

#### Data Deposition

Crystal structures have been deposited in the Protein Data Bank (http://www.pdb.org), Small-angle scattering data and models in SASBDB (http://www.sasbdb.org), mass spectrometry data to the ProteomeXchange partner MassIVE (http://massive.ucsd.edu) and ChIP-seq data at ArrayExpress (http://www.ebi.ac.uk/arrayexpress; accession E-MTAB-5670).

The accession numbers for the mass spectrometry data reported in this paper are MassIVE: MSV000081006, MSV000081001, MSV000080981, MSV000080986, MSV000080988, MSV000082857, MSV000082859 (https://massive.ucsd.edu). Additional files include the complete SAINTexpress outputs for each dataset as well as a ‘‘README’’ file that describes the dataset composition and the experimental procedures are associated with each accession number. Accession numbers for the coordinates and structure factors reported in this paper are PDB (http://www.rscb.org): 5NNC; 5NND; 5NNE; 5NNF; 5NNG; 6G0P; 6G0O; 6G0Q; 6G0R; and 6G0S. Small-angle X-Ray scattering data and models reported in this paper are SASD: SASDCT2; SASDCS2; SASDCR2; SASDCU2 (https://www.sasbdb.org). ChIP-seq data reported in this paper were deposited with as E-MTAB-5670 at ArrayExpress (https://www.ebi.ac.uk/arrayexpress). All original source files (SPOT arrays, microscopy images and western blots) have been deposited to Mendeley Data and are available at https://data.mendeley.com/datasets/ under ascensions xtb4mkvf8f/1, jb4jjxsbb7/1 and fzvwgpjx88.1.

### Additional Resources

The scored interactions associated with quantitative values are available for browsing and searching at https://prohits-web.lunenfeld.ca (project “BET rewiring”).

## Supplemental Information

Document S1. Figures S1–S7

Table S1. Clones and Reagents Information, Related to Figures 1, 2, 3, 4, 5, 6, and 7

Table S2. Proteomics Datasets, Related to Figures 1, 2, 5, and 6

Table S3. Peptide SPOT Arrays Profiling Kac Epitopes, Related to Figures 3 and 5, Amino-Acid Positional Enrichment around Kac Epitopes, Related to Figures S3 and S4, and Quantifications for BET ET Interactions, Related to Figures 5, S5, and S6

Table S4. Isothermal Titration Calorimetry (ITC) and Analytical Ultracentrifugation (AUC) Results, Related to Figures 3 and 4 and Figures S3 and S4

Table S5. Data Collection and Refinement Statistics, Related to Figures 3 and 4

Document S2. Article plus Supplemental Information
