# Supplementary material for: Interactome Rewiring Following Pharmacological Targeting of BET Bromodomains
Source: Mol Cell. 2019 Feb 7;73(3):621–638.e17. doi: 10.1016/j.molcel.2018.11.006 (PMC6375729; doi:10.1016/j.molcel.2018.11.006)
Supplement: Document S1. Figures S1–S7 [file mmc1.pdf]

Molecular Cell, Volume *73*

Supplemental Information

Interactome Rewiring Following

Pharmacological Targeting of BET Bromodomains

Jean-Philippe Lambert, Sarah Picaud, Takao Fujisawa, Huayun Hou, Pavel Savitsky, Liis Uusküla-Reimand, Gagan D. Gupta, Hala Abdouni, Zhen-Yuan Lin, Monika Tucholska, James D.R. Knight, Beatriz Gonzalez-Badillo, Nicole St-Denis, Joseph A. Newman, Manuel Stucki, Laurence Pelletier, Nuno Bandeira, Michael D. Wilson, Panagis Filippakopoulos, and Anne-Claude Gingras

Supplemental Information

Interactome rewiring following pharmacological targeting of BET bromodomains

Jean-Philippe Lambert, Sarah Picaud, Takao Fujisawa, Huayun Hou, Pavel Savitsky, Liis Uusküla-Reimand, Gagan D. Gupta, Hala Abdouni, Zhen-Yuan Lin, Monika Tucholska, James D.R. Knight, Beatriz Gonzalez-Badillo, Nicole St-Denis, Joseph A. Newman, Manuel Stucki, Laurence Pelletier, Nuno Bandeira, Michael D. Wilson, Panagis Filippakopoulos, Anne-Claude Gingras

Supplemental Figure S1 (supporting Figures 1 & 2)

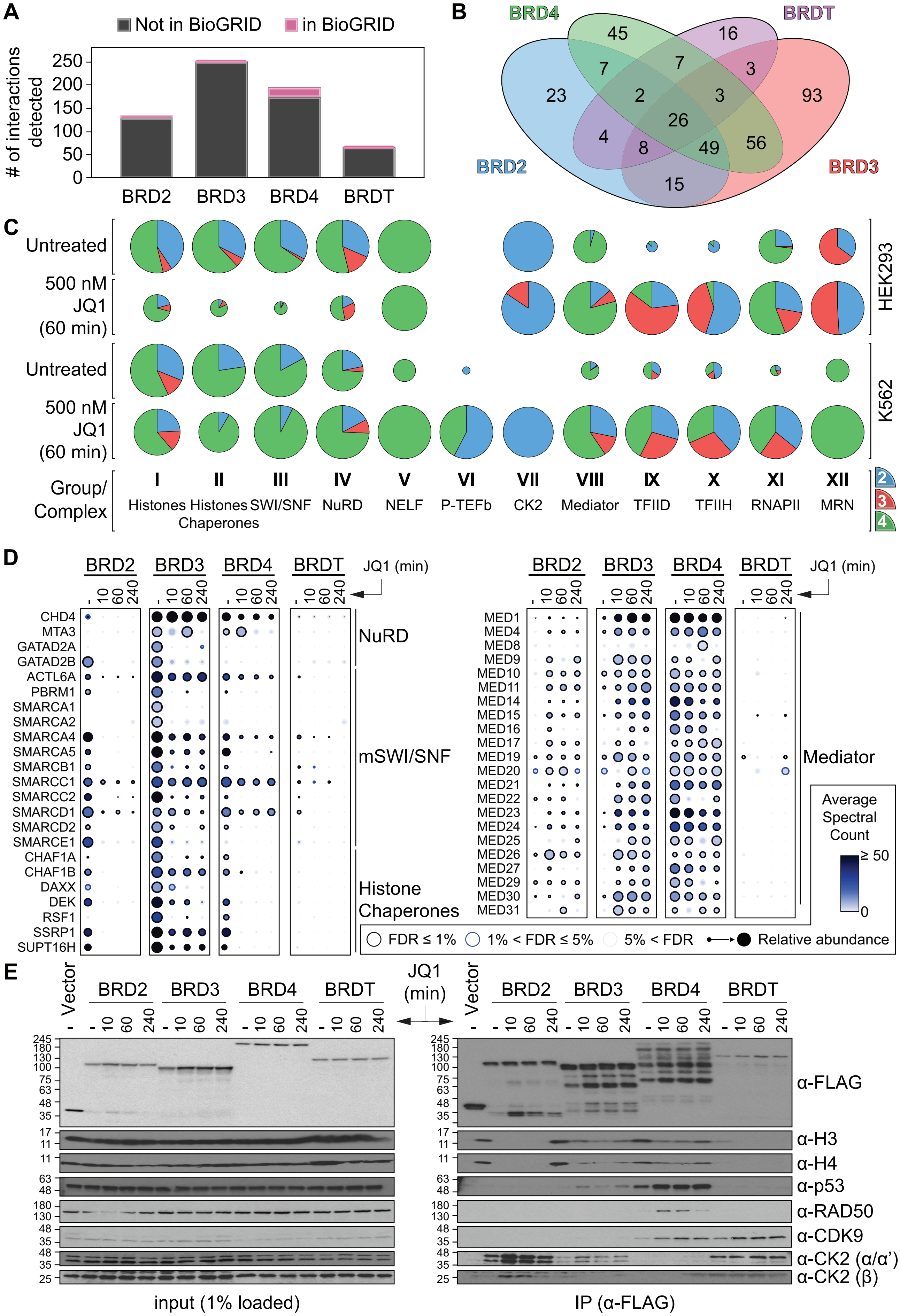

Supplemental Figure S1 (supporting Figures 1 & 2) – *BET interactome survey using affinity purification coupled to mass spectrometry (AP-MS) and its modulation following JQ1 treatment* (**A**) Bar graph showing the number of significant interaction partners detected for each BET protein using AP-MS with a SAINTexpress FDR ≤ 1%. Interaction partners previously found in BioGRID (version 3.4.157, released on January 25th, 2018) are shown in pink while novel partners are in grey. (**B**) Venn diagram showing the overlap between significant interaction partners (SAINTexpress FDR ≤ 1%) for each BET protein by AP-MS. (**C**) Endogenous IP-MS validation of the BET interactome in HEK293 (top) and K562 (bottom) cells. Relative spectral count contributions of individual BET proteins to selected protein complexes or protein families, shown in pie charts. The relative abundances of selected protein complexes before and after JQ1 treatment are represented by the radius of the pie chart. BET proteins are color-coded as annotated in the inset. (**D**) Dot plot of selected interaction partners associated with BRD2, BRD3, BRD4 or BRDT after 500 nM JQ1 treatment for 0, 10, 60 or 240 min. Inner circle colour intensity represents the average spectral counts, the circle size represents the relative prey abundance across all samples shown and the circle outer edge corresponds to the SAINT FDR. Selected protein complexes or functional classes are indicated (See **Figure 2C** for other examples). (**E**) Validation of a subset of protein-protein interactions depicted in **Figure 2C** by immunoprecipitation coupled to immunoblotting. Vector (BirA*-NLS-FLAG) is a cell line expressing a nuclear-localized FLAG-BirA* construct, used here as a negative control. BET proteins (BRD2/3/4/T) are expressed as 3xFLAG-constructs. See STAR methods and **Table S1C** for antibody details and **Tables S2** for AP-MS details.

Supplemental Figure S2 (supporting Figure 3)

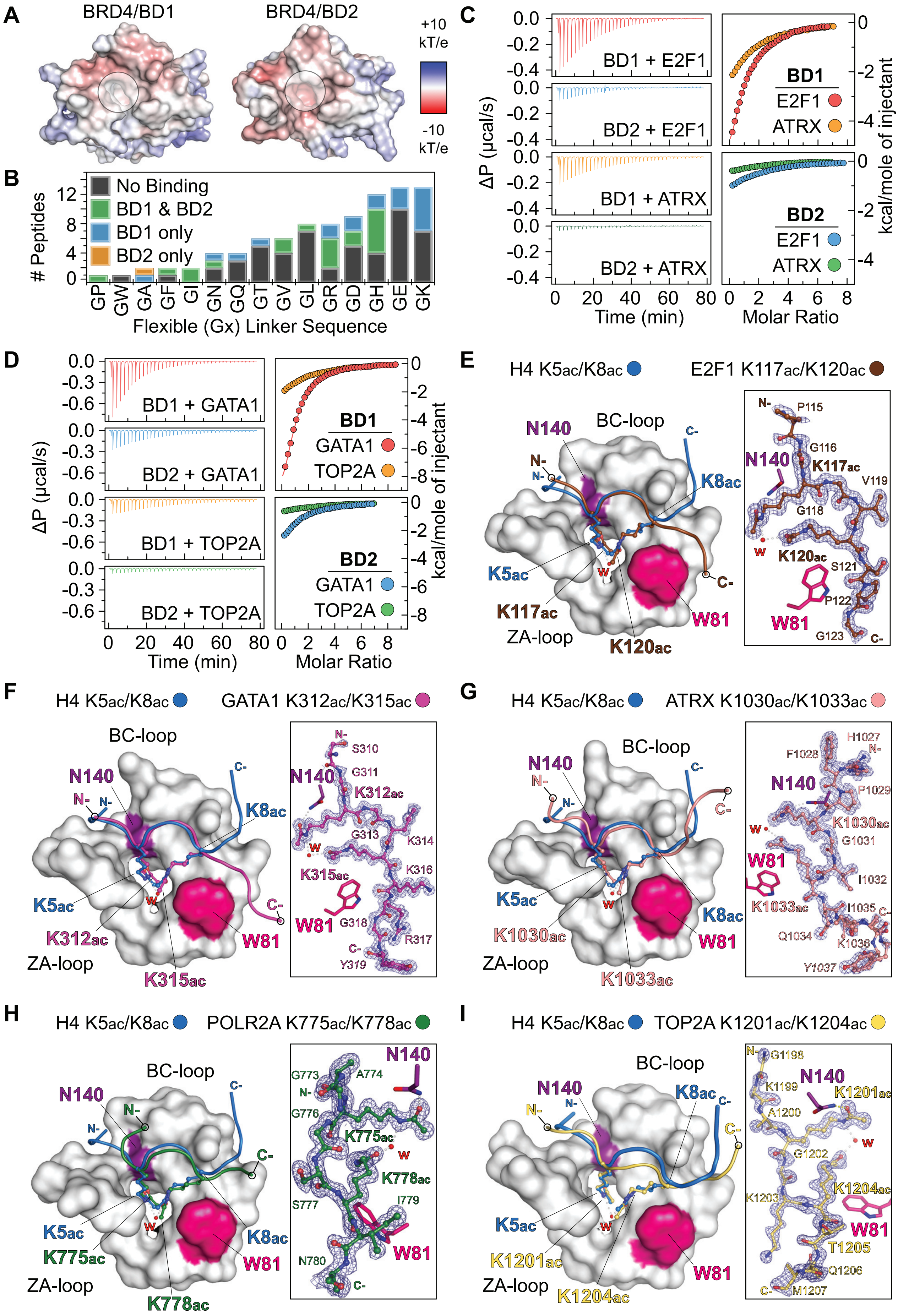

Supplemental Figure S2 (supporting Figure 3) – *BET BRDs recognize acetyl lysines in non-histones proteins with a histone-like structural template.* (**A**) Surface map of the first and second bromodomains of BRD4 showing the electrostatic potential. (**B**) Summary of the SPOT array results for BRD4/BD1 and BD2 binding to Kac-GX-Kac sequences, carrying a flexible glycine linker at X1, presented in **Table S3C** & **S3D**. (**C**) In solution evaluation of BRD4/BD1 and BRD4/BD2 binding to E2F1 and ATRX Kac-XX-Kac peptides by isothermal titration calorimetry (ITC). Raw injection heats for titrations of peptides into a solution of BRD4/BD1 or BRD4/BD2 are shown in the left panels. The right panels show the normalized binding enthalpies corrected for the heat of peptide dilution as a function of binding site saturation (symbols as indicated in the figure). Solid lines represent a nonlinear least squares fit using a single-site binding model. Titrations were performed in 50 mM HEPES pH 7.5 (at 25°C), 150 mM NaCl and 15°C while stirring at 1,000 rpm. (E2F1: K_D = 45.8 μM and 34.8 μM for BD1 and BD2 respectively. ATRX: K_D = 87.7 μM and 25.31 μM for BD1 and BD2 respectively). (**D**) ITC evaluation of BRD4/BD1 and BRD4/BD2 binding GATA1 and TOP2A Kac-XX-Kac peptides. Experiments were conducted and are presented as in (C). GATA1: K_D = 24.6 μM and 62.9 μM for BD1 and BD2 respectively. TOP2A: K_D = 19.5 μM and 63.3 μM for BD1 and BD2 respectively. (**E**) Binding detail of an E2F1 Kac-XX-Kac peptide (K117ac/K120ac, shown in brown) with BRD4/BD1 and comparison to an H4 K5ac/K8ac peptide (shown in blue). The surface of BRD4/BD1 is shown in grey with the conserved asparagine (N140) highlighted in purple and the bulky tryptophan (W81) that caps the cavity highlighted in magenta. The peptide inserts in the cavity with K117ac directly engaging N140, while K120ac engages via a water-mediated bridge to K117, stabilizing the interaction. This is the same structural template observed in the case of the H4 K5ac/K8ac peptide. The inset shows the high resolution 2FcFo map of the peptide contoured at 2σ and highlights the topology with respect to N140 and W81. (**F**) Binding details of a GATA1 Kac-XX-Kac peptide (K312ac/K315ac, shown in magenta) with BRD4/BD1 and comparison to an H4 K5ac/K8ac peptide (shown in blue). Binding details are presented as in (E). (**G**) Binding details of an ATRX Kac-XX-Kac peptide (K1030ac/K1033ac, shown in salmon) with BRD4/BD1 and comparison to an H4 K5ac/K8ac peptide (shown in blue). Binding details are presented as in (E). (**H**) Binding details of a POLR2A Kac-XX-Kac peptide (K1201ac/K1204ac, shown in green) with BRD4/BD1 and comparison to an H4 K5ac/K8ac peptide (shown in blue). Binding details are presented as in (E). (**I**) Binding details of a TOP2A Kac-XX-Kac peptide (K1201ac/K1204ac, shown in yellow) with BRD4/BD1 and comparison to an H4 K5ac/K8ac peptide (shown in blue). Binding details are presented as in (D).

Supplemental Figure S3 (supporting Figure 4)

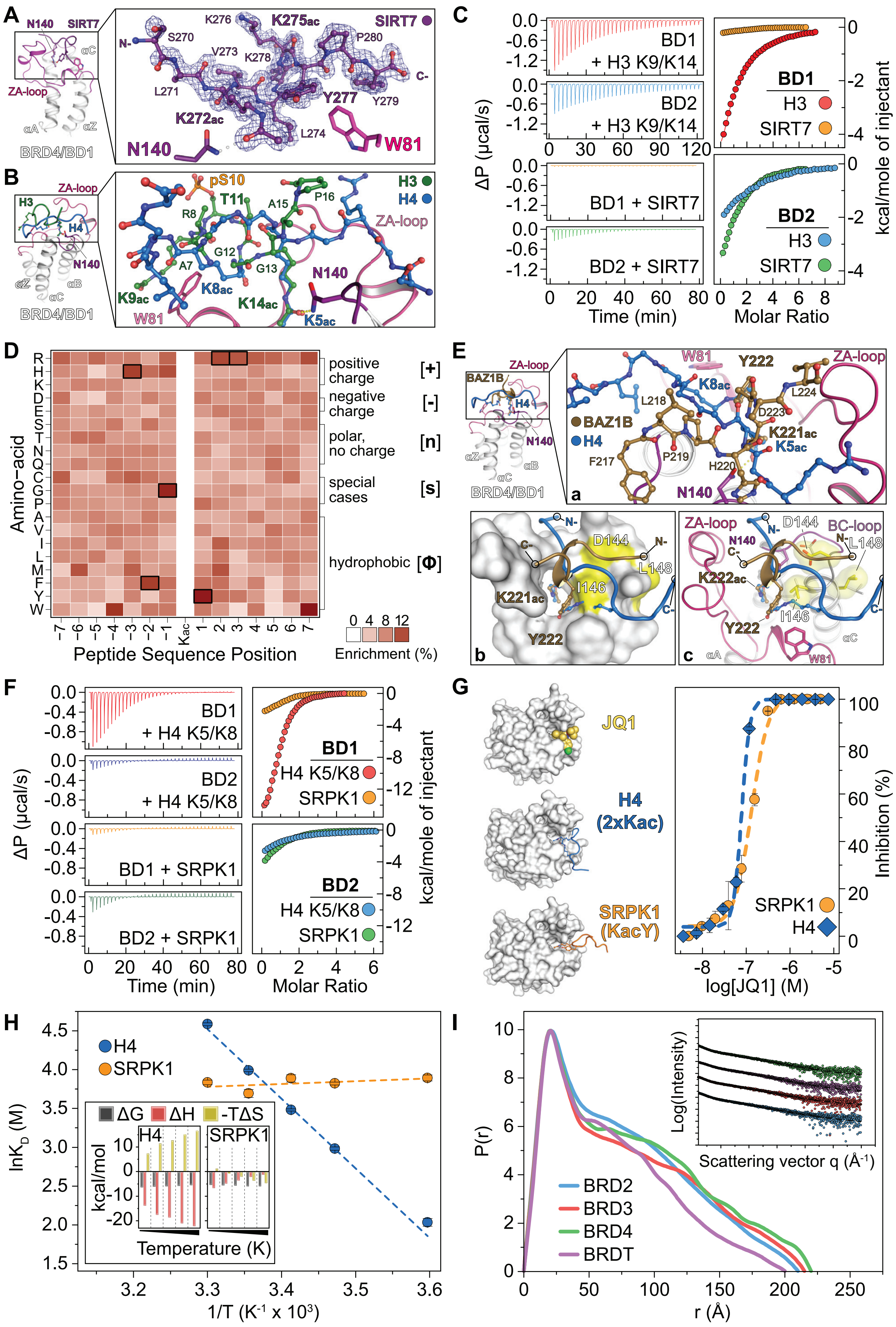

Supplemental Figure S3 (supporting Figure 4) – *BET BRDs can recognize acetyl lysines using different modes of binding* (**A**) Binding detail of a SIRT7 Kac-XX-Kac peptide (K272ac/K275ac, shown in purple) with BRD4/BD1. While K272ac initiates a contact to N140, Y277 stabilizes the interaction via direct hydrogen bonding to K272 and K275ac does not insert within the cavity. The peptide 2FcFo high resolution map contoured at 2σ is shown. (**B**) Comparison of acetylated H3 and H4 binding onto BRD4/BD1. Both peptides insert an acetylated lysine in the BRD Kac recognition cavity between W81 and N140. While in H4 a second Kac sterically fills the cavity (K8ac), the H3 peptide adopts an inverted conformation, with the C-terminus close to N140 and the N-terminus close to W81, K9ac packing next to W81 and K14ac inserting into the cavity (resembling K5ac in H4 from PDB: 3UVW). The peptide backbone (A7 through G13) adopts a helical turn substituting the K8ac insertion into the cavity, while positioning S10 and T11 in a way that allows binding upon phosphorylation of S10 but not of T11. (**C**) In solution evaluation of BRD4/BD1 and BRD4/BD2 binding to a histone H3 peptide acetylated at K9 and K14 and a SIRT7 peptide acetylated at K272ac and K275ac by isothermal titration calorimetry (ITC). Raw injection heats for titrations of peptides into a solution of BRD4/BD1 or BRD4/BD2 are shown in the left panels. The right panels show the normalized binding enthalpies corrected for the heat of peptide dilution as a function of binding site saturation (symbols as indicated in the figure). Solid lines represent a nonlinear least squares fit using a single-site binding model. All ITC titrations were performed in 50 mM HEPES pH 7.5 at 25°C, 150 mM NaCl and 15°C while stirring at 1,000 rpm. (**D**) Relative enrichment of amino acids surrounding a central annotated Kac site found in Kac-dependent (and JQ1-sensitive) BRD4 interactions extracted from the AP-MS data. The boxed positions represent the highest enrichment for a particular row compared to every other position in the same row. Lysine enrichment at +3 is in agreement with our previously determined Kac-XX-Kac binding motif for BETs (Filippakopoulos et al., 2012), though here it does not seem to drive the interactions. Enrichment of tyrosine at +1 (Kac-Y motif) represents sequences found in histone H1 as well as other proteins (such as BAZ1B). (**E**) Comparison of BRD4/BD1 complex with an acetylated BAZ1B K221ac peptide to one with H4 K5ac/K8ac (PDB: 3UVW). The BAZ1B peptide defines a vector perpendicular to the H4 backbone, while initiating contact with N140 via K221ac (shown in a). Side-chain rotations of single residues (D144, I146, L148 highlighted in yellow in panel b) on the surface of BRD4/BD1 result in remodelling of the exposed BRD4/BD1 surface, allowing insertion of a 3_{10} helix from BAZ1B into the Kac cavity, with Y222 mimicking K8ac. Panel c shows a cartoon representation of the side-chain rotations of D144, I146 and L148 (in yellow as sticks with translucent surfaces) compared to the H4 complex (residues shown as white sticks). (**F**) ITC validation of SRPK1 K585ac/K588ac (carrying a Kac-Y motif) binding to BRD4 BRDs and comparison to a histone H4 K5ac/K8ac peptide. Data are represented as in (C) and titrations were performed in 100 mM Na_3PO_4 pH 8.0, 150 mM NaCl at 15°C while stirring at 1,000 rpm. Both peptides show the same affinity for BRD4/BD1 (K_D = 9.9 μM) while BRD4/BD2 shows weaker binding (K_D = 113.6 μM and 29.7 μM for H4 and SRPK1 respectively). (**G**) JQ1 competition for the Kac binding cavity of BRD4/BD1. The complexes of BRD4/BD1 with JQ1 (yellow, top; PDB: 3MXF), H4 (carrying 2xKac sites, K5ac/K8ac, blue, middle; PDB: 3UVW) and SRPK1 (carrying a KacY motif, orange, bottom) are shown on the left. ALPHA-Screen™ competition assay demonstrating competitive displacement of an H4 K5ac/K8ac (blue) peptide and an SRPK1 KacY peptide (orange). JQ1 inhibits the BRD4/BD1 interaction with H4 and SRPK1 with IC_{50} values of 78.5 and 132 nM respectively. (**H**) Thermodynamic analysis of BRD4/BD1 interactions with H4 and SRPK1 peptides. The van't Hoff plot of the dissociation constant (K_D) as a function of reverse temperature (T^{-1}) shows the characteristic pattern of endothermic (H4) and exothermic (SRPK1) reactions. The dotted lines represent a least square linear fit. The inset shows thermodynamic parameters extracted from fitted ITC data in. While both interactions are characterized by negative free energy changes, increasing temperature results in more negative enthalpies offset by more positive entropic contributions (H4, left) and decreasing enthalpies assisted by decreasing entropic contributions (SRPK1, right). (**I**) Small angle X-Ray diffraction of tandem BET BRDs. The pairwise distance distribution function (P(r)) exhibits the characteristic shape of an elongated particle with a long flexible linker. The inset shows experimental scattering patterns (log(I) y-translated) from tandem BET BRDs. The dotted black line shows the fit computed from the best representative randomly generated model.

Supplemental Figure S4 (supporting Figure 5)

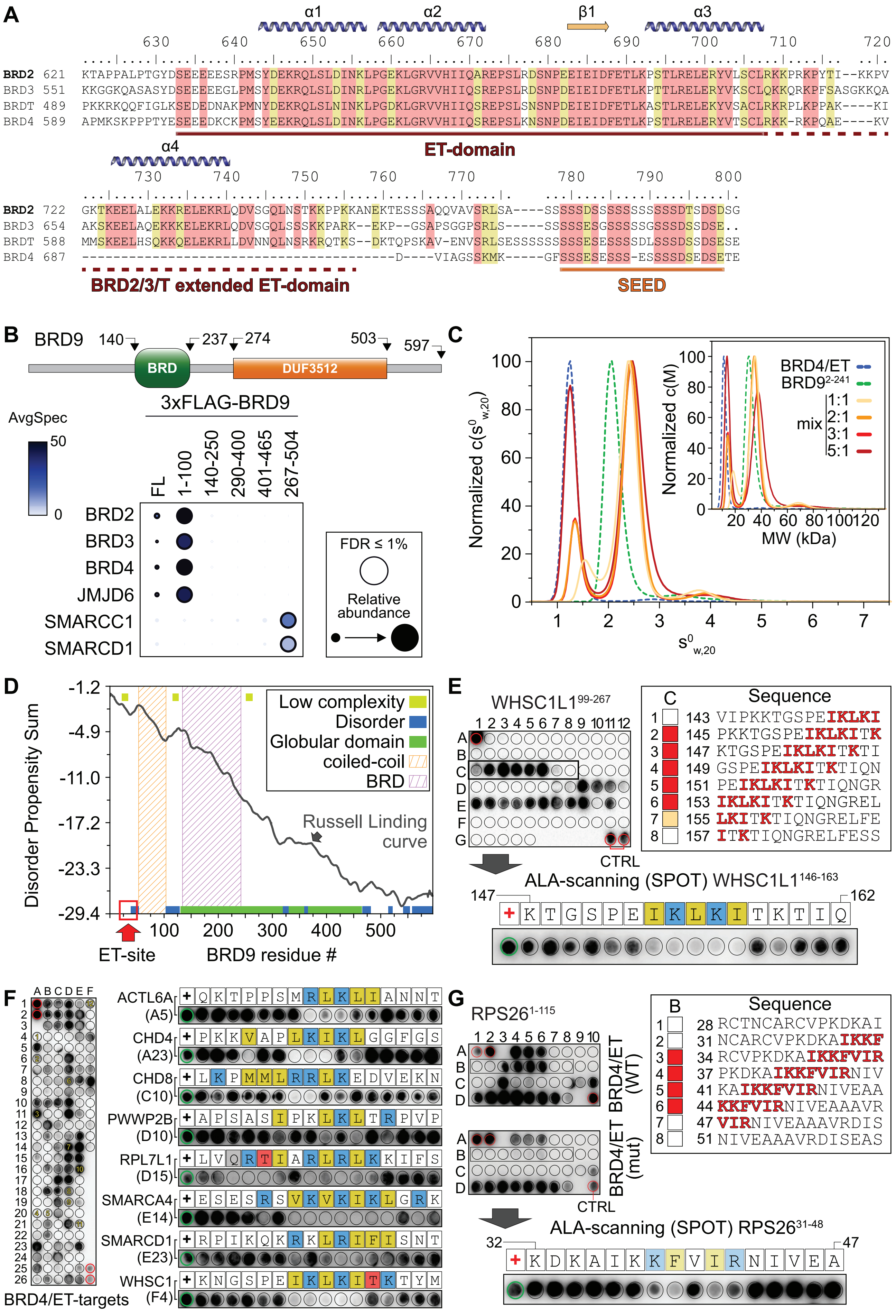

Supplemental Figure S4 (supporting Figure 5) – *The extra-terminal (ET) domain binds to specific short linear motifs*. (**A**) Sequence alignment of the BET ET domains. Secondary structure prediction identified a helix (α4) following the three helices found on the previously described ET domain (Lin et al., 2008) which is not present in BRD4. All BET ET domains are followed by a serine-glutamate-aspartate (SEED) rich region. (**B**) Modular architecture of BRD9 with domain boundaries indicated (*top*). Recovery of endogenous BETs and selected BRD9 interaction partners in AP-MS analysis of 3xFLAG-tagged BRD9 deletion constructs expressed in HEK293 cells (see **Table S2G** for a complete list of high-confidence interactors and the legend inset for details). (**C**) In-solution characterization of BRD4/ET and N-terminal BRD9 (residues 2–241) by interaction sedimentation velocity (SV) analytical ultracentrifugation (AUC). Sedimentation velocity plots of the normalized differential sedimentation coefficient distribution (c(s)), versus the apparent sedimentation coefficient corrected to water at 20°C, ($s_{20,w}$) are shown in the main panel, together with the normalized differential molecular weight distribution (c(M)) versus the system mass (M, shown in the inset). Both BRD4/ET and $BRD9^{2\text{-}241}$ sediment as monomers in solution, and associate as demonstrated by mixing different molar ratios of BRD4/ET with $BRD9^{2\text{-}241}$, suggesting weak association (higher molar ratio of ET shifts the equilibrium towards the complex). Experiments were conducted in 50 mM HEPES, pH 7.5, 100 mM NaCl at 4°C. (**D**) BRD9 disorder prediction using GlobPlot (http://globplot.embl.de/). The ET interaction motif is located on the N-terminal disordered portion of the protein. (**E**) Mapping of the previously reported $WHSC1L1^{99\text{-}267}$ interaction with BRD4/ET (Shen et al., 2015) using a peptide SPOT array (15 aa window, 2 aa overlap), identified a motif in the 146–163 region which was further profiled by alanine-scanning using SPOT arrays (bottom) to reveal residues important for the $WHSC1L1^{99\text{-}267}$-BRD4/ET interaction, defining the "WHSC1L1-like" ET-interaction motif. Sensitive positions are highlighted (yellow, hydrophobic; blue, positively charged). (**F**) SPOT peptide array spanning BRD9-like and WHSC1/L1-like motifs identified in the 12 proteins shown in **Figure 5A**. Representative peptides were further subjected to alanine-scanning to determine the specific contribution from each amino acid position to peptide binding (right panel). Sensitive positions are highlighted (yellow: hydrophobic; blue: positively charged; red: negatively charged). (**G**) Mapping of the RPS26 interaction with BRD4/ET using a peptide SPOT array (15 aa window, 3 aa overlap), identified a motif in the 31-48 region which was only recognized by $BRD4/ET^{WT}$ and not $BRD4/ET^{mut}$. This region was further profiled by alanine-scanning using SPOT arrays (bottom) to reveal residues important for the RPS26 -BRD4/ET interaction, which included a Phe residue (instead of Leu/Ile/Val/Met) within a "BRD9-like" motif. Sensitive positions are highlighted as in (E).

Supplemental Figure S5 (supporting Figure 5)

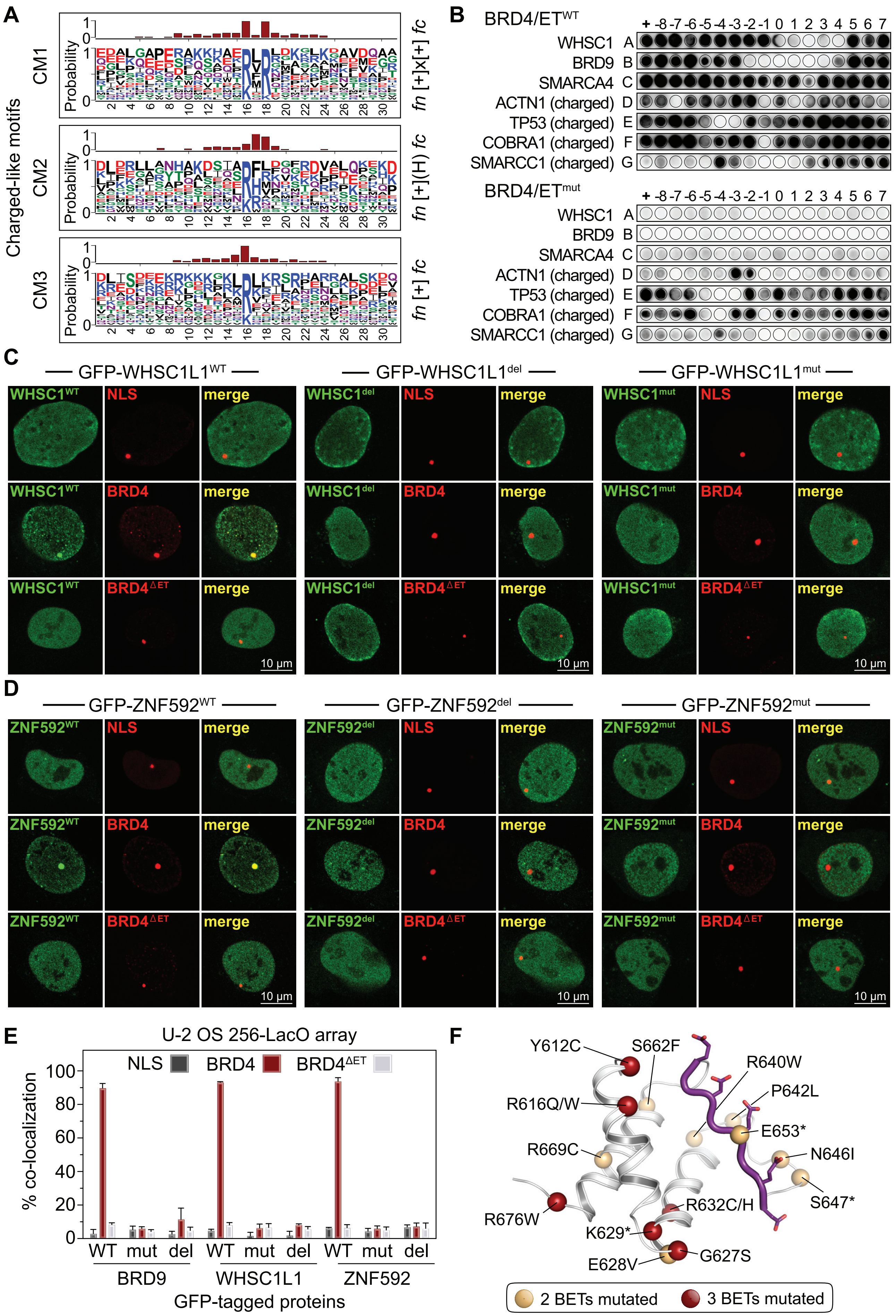

Supplemental Figure S5 (supporting Figure 5) - *Specific short linear motifs for extra-terminal (ET) domain binding are necessary for interactions in cells* (**A**) Potential non-specific ET-interacting motifs enriched from AP-MS BRD4 interacting partners identified in SPOT arrays and verified with alanine scanning. The three charged-like motifs (CM1: *fn*- [+]x[+]-*fc*; CM2: *fn*- [+](H)-*fc*; and CM3: *fn*-[+]-*fc* , where Φ is one of M,L,V,I,F and [+] is K or R) identified (shown in LOGO format) exhibit high dependency on the charged residues (shown in the relative contribution of each residue to binding, as determined in alanine scanning experiments and depicted as red bars at the top of each graph). (**B**) Verification of ET-binding motifs employing SPOT arrays and alanine scanning. Interactions revealed with the recombinant BRD4/ET domain (WT, top membrane and 4K mutant, lower membrane) demonstrate the non-specific character of the charged motifs (*CM1, CM2 and CM3* shown in A), in contrast to the specific BRD9-like and WHSC1/WHSC1L1-like motifs. (**C**) Validation of specific interactions between the BRD4/ET domain and the WHSC1L1 SLiM in cells employing a LacO/LacR chromatin immobilization assay. U-2 OS cells with a stably integrated 256 LacO array, were transfected with FL-mCherry-BRD4 (WT or ΔET) and FL-GFP-WHSC1L1 (WT or mutant). BRD4 localizes on the LacO array (red dots) together with FL-GFP- WHSC1L1^{WT} (green dots, left); overlap is shown in the merge panel (yellow dots). Deletion of the entire ET domain (AA 589–676) results in loss of the interaction (left, lower panel), as does deletion of the ET-motif within WHSC1L1 (GFP-WHSC1L1^{del}, lacking amino acids 143–161, middle panels), or mutation of this motif (K154A/L155A/K156A, GFP- WHSC1L1^{mut}, right panels). (**D**) LacO/LacR validation of ET-specific interactions in cells between BRD4 and ZNF592. U-2 OS cells with a stably integrated 256 LacO array were transfected with FL-mCherry-BRD4 (WT or ΔET) and FL-GFP-ZNF592 (WT or mutant). BRD4 localizes on the LacO array (red dots) together with GFP-ZNF592^{WT} (green dots, left). Deletion of the ET domain (amino acids 589–676) results in loss of the interaction (left, lower panel), as does deletion of the ET-motif within ZNF592 (GFP- ZNF592^{del}, lacking amino acids 364–379 middle panels), or mutation of this motif (K374A/V375A/R376A, GFP-ZNF592^{mut}, right panels). (**E**) High content analysis (Operetta 96-well plate format) confirmed these observations in the case of full-length GFP-tagged BRD9, WHSC1L1 and ZNF592 (WT or mutant proteins with an ET-motif deletion or mutation as in (D) following co-localization with mCherry- and LacR-tagged BRD4 (WT or ΔET). In each experiment 50-300 cells were monitored. (**F**) Patient mutations from the Cancer Genome Atlas (TCGA; https://cancergenome.nih.gov) mapped onto the NMR structure of the BRD4/ET domain (PDB ID: 2N3K). Residues found mutated in at least 2 (beige spheres) or 3 (red spheres) BET proteins are shown.

Supplemental Figure S6 (supporting Figure 6)

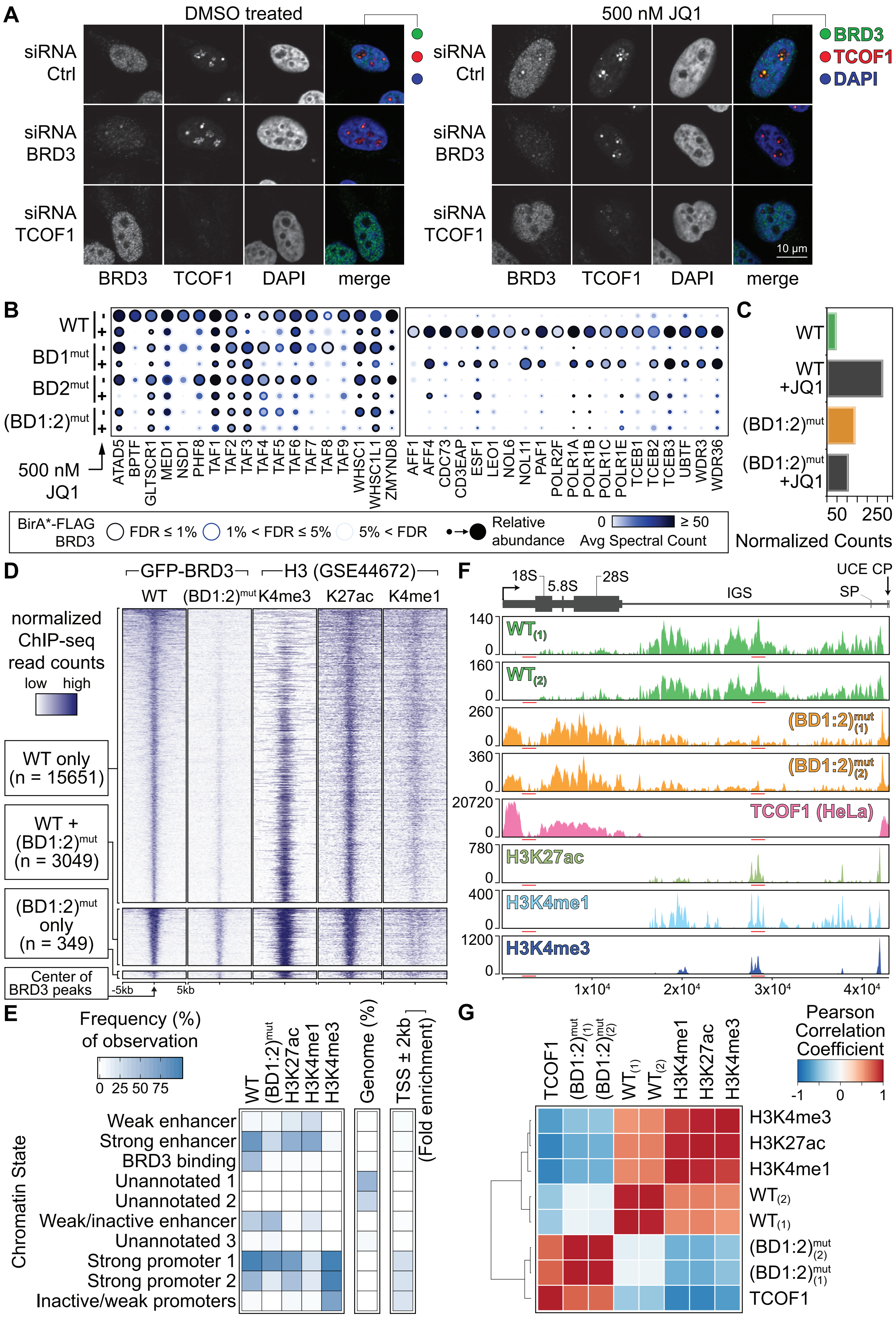

Supplemental Figure S6 (supporting Figure 6) - *BRD inhibition impacts the cellular localization of BET proteins and interactions with proteins and DNA.* (**A**) HeLa cells transfected for 48 h with indicated siRNAs (see **Table S1C** for all siRNA details) were treated with DMSO or 500 nM JQ1 for 1 h prior to fixing and staining with antibodies targeting BRD3 and TCOF1 and with DAPI. (**B**) Dot plot of selected interaction partners associated with BRD3 proteins (WT, $BD1^{mut}$, $BD2^{mut}$ or $(BD1:2)^{mut}$ detected by BioID with and without 500 nM JQ1 treatment for 24 h (see legend inset). See **Table S2M** for complete results. (**C**) Bar graph displaying the background-subtracted spectral counts for TCOF1 as a BioID hit for the indicated constructs and conditions. (**D**) Heatmap showing the normalized ChIP-seq signal intensity of merged replicates of BRD3-WT and BRD3 $(BD1:2)^{mut}$. ChIP-seq data from U-2 OS cells for H3K4me3, H3K27Ac and H3K4me1 as previously reported (arrayExpress ID E-GEOD-44672, (Walz et al., 2014)). After intersecting ChIP-seq peaks, BRD3 binding sites were classified as “WT only”, “WT shared with $(BD1:2)^{mut}$” and “$(BD1:2)^{mut}$”. Within each category, binding sites were rank-ordered based on the intensity of BRD-WT ChIP-seq signal. ChIP-seq signal at +/- 5 kb around the centres of BRD3 binding sites was averaged in 50 bp bins. Colour intensity represents the normalized ChIP-seq read counts (read counts are normalized to 10 million per library). (**E**) A heatmap showing the emission parameters where each row represents a different state, the first five columns (left to right) represent different factors. States are annotated on the graph. For the first five columns, the darker the color, the higher the probability of observing the factor in the corresponding state (observation frequency (%)). The second last columns (“Genome”) represents the percentage of genome each state overlaps. The last column (TSS +/- 2 kb) represents the fold enrichment of each state for regions 2 kb +/- from TSSs. The same color scale applies to the whole heatmap. (**F**) Genome browser view showing the ChIP-seq signal of *BRD3, $(BD1:2)^{mut}$, TCOF1*, H3K27ac (active promoters and enhancers), H3K4me1 (enhancers) and H3K4me3 (promoters) at the rDNA locus. The structure of the rDNA locus is shown on the top (as per **Figure 6D**). X axis: relative genomic location on the rDNA loci; y axis: normalized counts (normalized to 10 million reads for each library). (**G**) Correlation heatmap of signal of different factors on the rDNA locus. Colour intensity presents Pearson correlation coefficient. Hierarchical clustering was performed based on the pair-wise correlation coefficients.

Supplemental Figure S7 (supporting Figure 7)

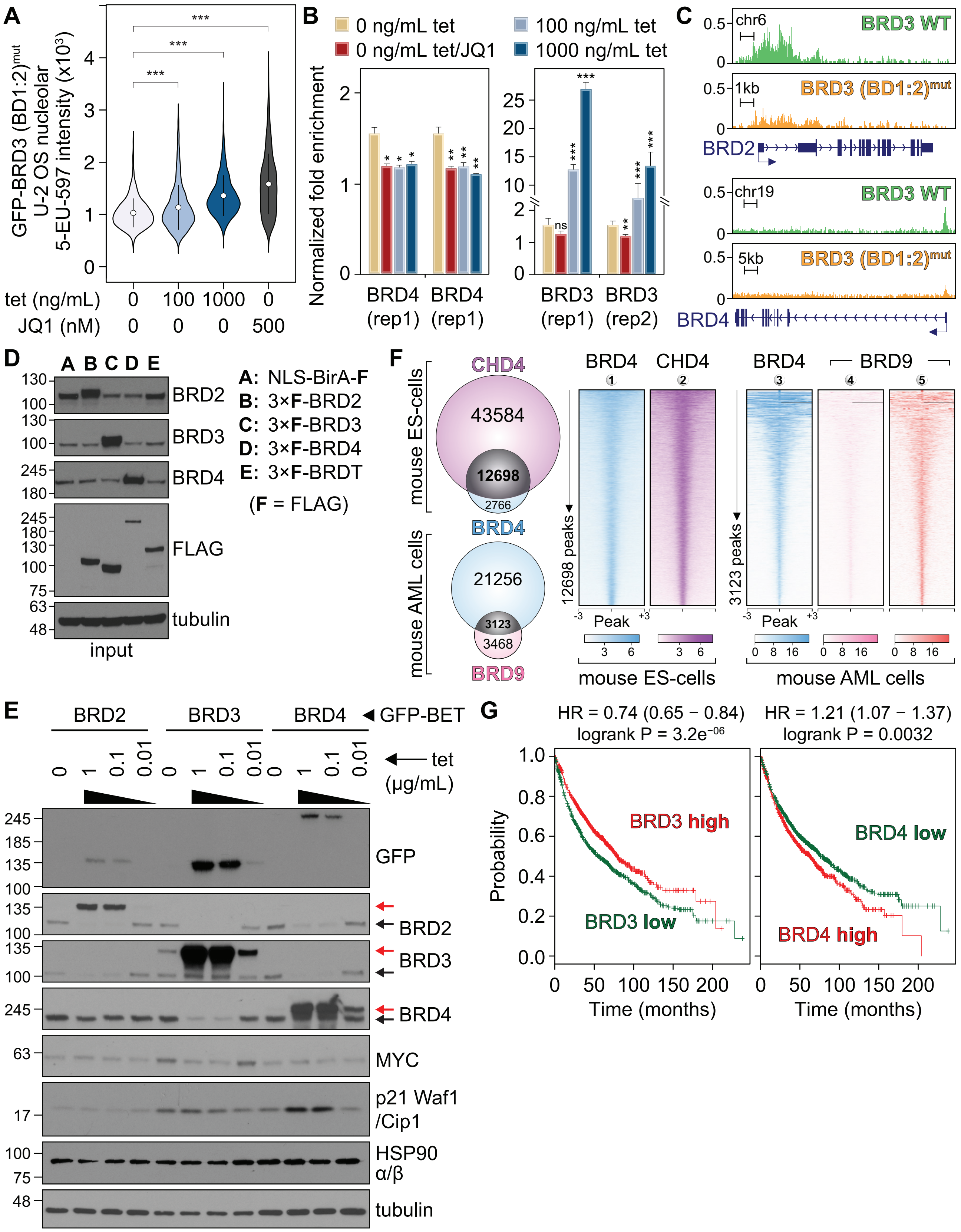

Supplemental Figure S7 (supporting Figure 7) - *BRD3 impacts rRNA production and cell proliferation.* (**A**) Quantitative immunofluorescence of U-2 OS cells treated with the indicted concentrations of tetracycline (to induce BRD3 (BD1:2)^{mut} expression) or JQ1 was performed as per **Figure 7B**. The 5-EU signal overlapping with the fibrillarin signal (i.e. nucleolar RNA) was quantified in > 400 cells for each experimental condition. *P* values were calculated using the two-tailed Student *t*-test statistic and are represented so that $P < 0.001$, "***". (**B**) ChIP-qPCR of endogenous BRD4 (left) and GFP-BRD3 (right) to the MYC promoter in GFP-BRD3 U-2 OS cells treated with increasing amounts of tetracycline or 500 nM JQ1. The x-axis represents the normalized fold enrichment over the signal obtained from rabbit isotype IgG control purifications. Data represent the mean ± SEM for each of the two biological replicates analysed across three technical replicates. *P* values were calculated using Student's *t*-test. ***, $P < 0.001$; **, $P < 0.01$; *, $P < 0.05$; ns, not significant. (**C**) Close-up view of gene tracks showing BRD3 (green track) and BRD3 (BD1:2)^{mut} (orange track) occupancy across the *BRD2* and *BRD4* gene loci. The y-axis represents the number of reads aligned to each genomic position. (**D**) Protein levels as detected by immunoblot in Flp-In T-REx HEK293 cells expressing 3×FLAG-tagged BRD2, BRD3, BRD4 or an NLS-BirA*-FLAG control induced with 1000 ng/mL tetracycline for 24 h (see STAR methods and **Table S1C**). (**E**) Protein levels as detected by immunoblot in Flp-In T-REx U-2 OS cells expressing GFP-tagged BETs induced with indicated concentrations of tetracycline for 48 h. Black arrows indicate endogenous BET while red arrows indicate GFP-tagged BETs. (**F**) Venn diagrams showing overlap of BRD4 and CHD4 ChIP-seq peaks in mouse embryonic stem cells (top) or BRD4 and BRD9 in mouse AML cells (bottom). Signal densities are shown on the right panels, for enrichment of CHD4 peak density around ±3 kb of BRD4 identified peaks in mESCs, as well as enrichment of BRD4, BRD9 and FLAG-BRD9 signal densities ±3 kb around BRD4 identified peaks in mouse AML cells. The signal scale is shown in the inset. Published data were used from GEO sets: **1**: GSE69140; **2**:GSE61188; **3**: GSE52279; **4**,**5**: GSE79360 as indicated in the inset. (**G**) Kaplan–Meier survival curves of 1928 patients with lung cancer from cohort studies CAARRAY, GSE14814, GSE19188, GSE29013, GSE30219, GSE31210, GSE3141, GSE31908, GSE37745, GSE43580, GSE4573, GSE50081, GSE8894 and TCGA were generated using KM plotter (http://kmplot.com/) for BRD3 (left) and BRD4 (right).

Supplemental References

Filippakopoulos, P., Picaud, S., Mangos, M., Keates, T., Lambert, J.P., Barsyte-Lovejoy, D., Felletar, I., Volkmer, R., Muller, S., Pawson, T., *et al.* (2012). Histone recognition and large-scale structural analysis of the human bromodomain family. Cell *149*, 214-231.

Lin, Y.J., Umehara, T., Inoue, M., Saito, K., Kigawa, T., Jang, M.K., Ozato, K., Yokoyama, S., Padmanabhan, B., and Guntert, P. (2008). Solution structure of the extraterminal domain of the bromodomain-containing protein BRD4. Protein Sci *17*, 2174-2179.

Shen, C., Ipsaro, J.J., Shi, J., Milazzo, J.P., Wang, E., Roe, J.S., Suzuki, Y., Pappin, D.J., Joshua-Tor, L., and Vakoc, C.R. (2015). NSD3-Short Is an Adaptor Protein that Couples BRD4 to the CHD8 Chromatin Remodeler. Mol Cell *60*, 847-859.

Walz, S., Lorenzin, F., Morton, J., Wiese, K.E., von Eyss, B., Herold, S., Rycak, L., Dumay-Odelot, H., Karim, S., Bartkuhn, M., *et al.* (2014). Activation and repression by oncogenic MYC shape tumour-specific gene expression profiles. Nature *511*, 483-487.
